# Synthesis, Antifungal Activities, Molecular Docking and Molecular Dynamic Studies of Novel Quinoxaline-Triazole Compounds

**DOI:** 10.1021/acsomega.3c02797

**Published:** 2023-06-26

**Authors:** Derya Osmaniye, Nurnehir Baltacı Bozkurt, Serkan Levent, Gamze Benli Yardımcı, Begüm Nurpelin Sağlık, Yusuf Ozkay, Zafer Asım Kaplancıklı

**Affiliations:** † Department of Pharmaceutical Chemistry, Faculty of Pharmacy, Anadolu University, 26470 Eskişehir, Turkey; ‡ Central Research Laboratory (MERLAB), Faculty of Pharmacy, Anadolu University, 26470 Eskişehir, Turkey; § Department of Pharmaceutical Microbiology, Faculty of Pharmacy, Afyonkarahisar Health Sciences University, 03030 Afyonkarahisar, Turkey

## Abstract

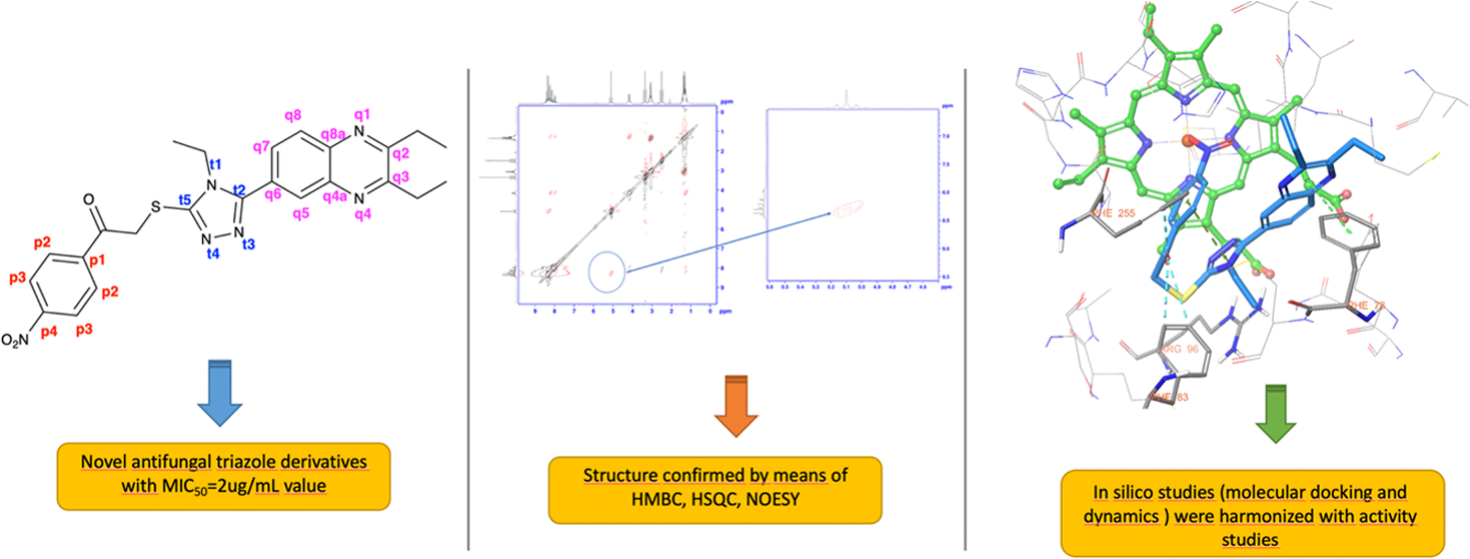

Uncontrolled use of antifungal drugs affects the development of resistance to existing drugs. Azole antifungals constitute a large part of antifungal therapy. Therefore, there is a need for new azole antifungals. Within the scope of this study, 17 new triazole derivative compounds were synthesized. Structure determinations were clarified by spectroscopic analysis methods (^1^H-NMR, ^13^C-NMR, HRMS). In addition, structure matching was completed using two-dimensional NMR techniques, HSQC, HMBC and NOESY. The antifungal effects of the compounds were evaluated on *Candida* strains by means of *in vitro* method. Compound **5d** showed activity against *Candida glabrata* with a MIC_90_ = 2 μg/mL. Compound **5d** showed activity against *Candida krusei* with a MIC_90_ = 2 μg/mL. This activity value, which is higher than fluconazole, is promising. In addition, the biofilm inhibition percentages of the compounds were calculated. Molecular docking and molecular dynamics simulations performed with compound **5d** are in harmony with activity studies.

## 1 Introduction

Invasive fungal infections (IFIs), especially in the last three decades, pose a serious global threat. Especially immunocompromised patient groups (those living with HIV-AIDS, cancer, organ transplant recipients and autoimmune patients) face this threat.^1^ It is estimated that the number of invasive fungal infections associated deaths is as high as 1.5 to 2 million worldwide each year.^2^ As these patients need to use this drug, it highlights the need to synthesize new classes of antimicrobial agents, particularly structurally diverse molecules with unique mechanism of action, high potency, less toxicity, and no or fewer side effects.^3^ In fact, fungal infections are easier to treat than bacterial and other infections. But unfortunately, resistance to drugs complicates the treatment process.^4^ Studies have shown that ergosterol biosynthesis is crucial for the survival of fungi, and many antifungal drug targets have been found based on these studies.^5^

As antifungal agents, azoles act through inhibition of fungal lanosterol 14-α-demethylases (CYP51), an enzyme required to catalyze the oxidative removal of 14-α-demethylases in sterol biosynthesis by fungi.^6^ Fluconazole is used in the treatment of fungal infections as a drug with high oral bioavailability and therapeutic index. To overcome some of the shortcomings when using these first generation agents, second generation analogues such as voriconazole, ketoconazole, albaconazole and posaconazole have been developed.^7^

Candida species are commensal organisms that can lead to infection ranging from minor to severe candidemia and invasive candidiasis.^8^ The most crucial and harmful quality of Candida species is their capacity to create biofilms, which makes finding effective treatments and battling disease more difficult.^9^ Biofilms are habitats in which microbial organisms are coated in a matrix of extracellular polymeric molecules (EPS). The biofilm manner of life is influenced by the matrix’s composition, characteristics, and dynamics.^10^ The protective shielding effect of biofilm is countered by the slow diffusion of therapeutic antifungals into its interior, which hinders the eradication of illness. Candida pathogenicity is mostly attributable to the release of biofilm development, and adherence to polymeric medical devices and/or host cells.^9^

Triazole is a quintuple aromatic ring system containing three nitrogen atoms. Many antifungal drugs are in their structure. It contributes to the activity by the bonds it forms with the HEM in the active site of the 14-α-demethylases enzyme. When the literature is examined, it has been proven by many studies that this ring has antifungal activity.^11−19^ There are literature studies showing the antifungal activities of not only 1,3,4-triazoles but also 1,2,4-triazoles and 1,2,3 triazoles.^20−24^

Molecular hybridization is one of the most frequently used methods to develop new compounds. Effective molecules can be reached with the synergistic effect created using two pharmacophore groups or two rings with known activity. In this study, besides the triazole ring, all the compounds also carry the quinoxaline ring system in common. It was thought that the quinoxaline ring would provide stability in the active site of the enzyme as an aromatic bulky group.

## 2 Results and Discussion

### 2.1 Chemistry

**Scheme 1:**
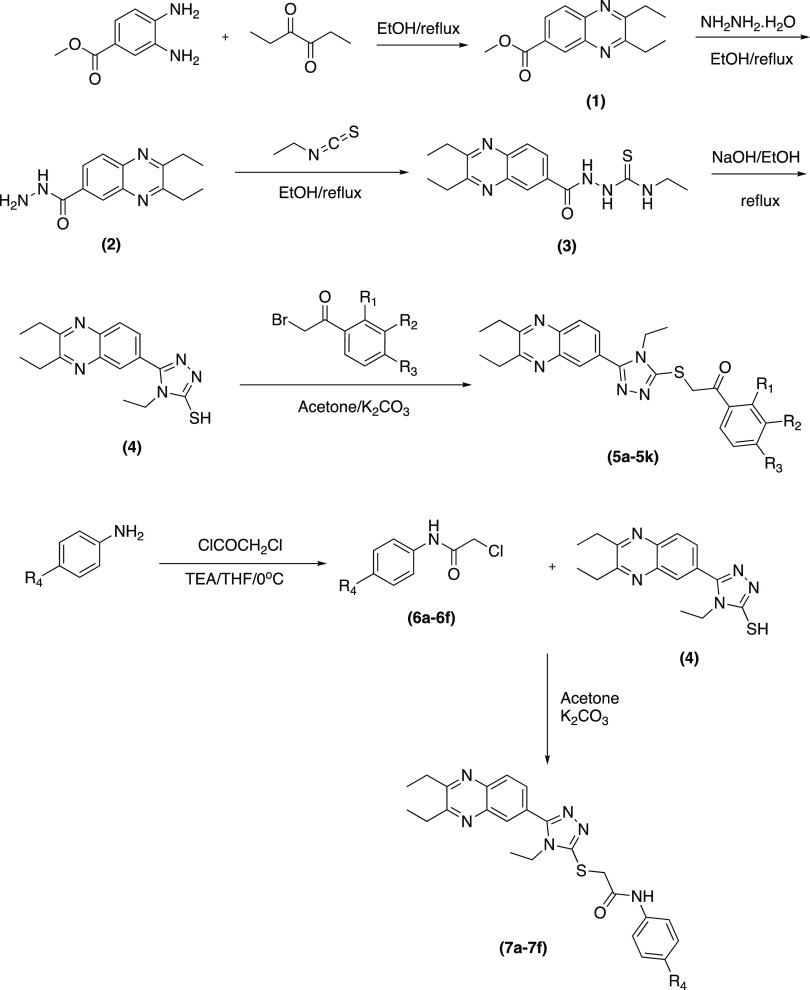
Synthesis Pathway for Obtained Compounds (**5a**–**5k** and **7a**–**7f**)

The synthesis scheme is presented in Scheme 1. A seven-step synthesis procedure was followed to obtain the target compounds. First, the methyl 3,4-diaminobenzoate and hexane-3,4-dione were refluxed for obtained methyl 2,3-diethylquinoxaline-6-carboxylate (compound **1**). Quinoxaline ring closure reaction lasted for 5 h. Second, compound **1** was converted to hydrazide derivative (compound **2**) by means of reflux system. Third, the thiourea derivative (compound **3**) was obtained using ethyl isothiocyanate. Then, triazole ring (compound **4**) was closed by using thiourea (compound **3**) in basic medium. The one part of target compounds (**5a**–**5l**) was obtained by reaction between triazole derivate (compound **4**) and appropriate 2-bromoacetophenone. Then, the anilines were acetylated for obtained compounds **6a**–**6f**. The other one part of target compounds (**7a**–**7f**) was obtained by reaction between triazole derivate (compound **4**) and obtained acetylated anilines (**6a**–**6f**).

**Figure 1:**
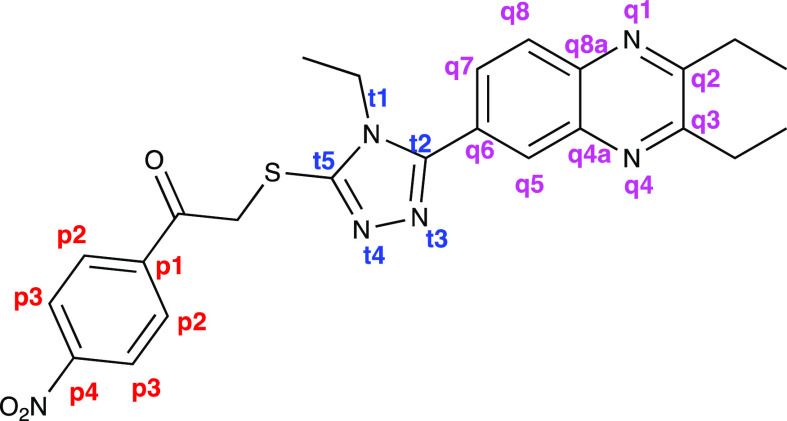
Molecular structure of compound **5d**.

The structures of the compounds obtained were established by spectroscopic methods, namely ^1^H-NMR, ^13^C-NMR, and HRMS (Table 1 and Supplementary Data). Thanks to the 2D NMR analyzes performed on the active compound (**5d**) (Figure 1), all hydrogen and carbons on the compound were matched. HSQC, HMBC, and NOESY were used as 2D NMR analysis technique.

**Table 1:** Molecular Structure of Obtained Compounds (**5a**–**5k** and **7a**–**7f**)

compounds	R_1_	R_2_	R_3_	R_4_
**5a**	-H	-H	-CH_3_	
**5b**	-H	-H	-OCH_3_	
**5c**	-H	-H	-CN	
**5d**	-H	-H	-NO_2_	
**5e**	-H	-H	-F	
**5f**	-H	-H	-Cl	
**5g**	-H	-H	-Br	
**5h**	-CH_3_	-H	-CH_3_	
**5i**	-F	-H	-F	
**5j**	-Cl	-H	-Cl	
**5k**	-H	-Cl	-Cl	
**7a**				-H
**7b**				-CH_3_
**7c**				-OCH_3_
**7d**				-F
**7e**				-Cl
**7f**				-CF_3_

**Figure 2:**
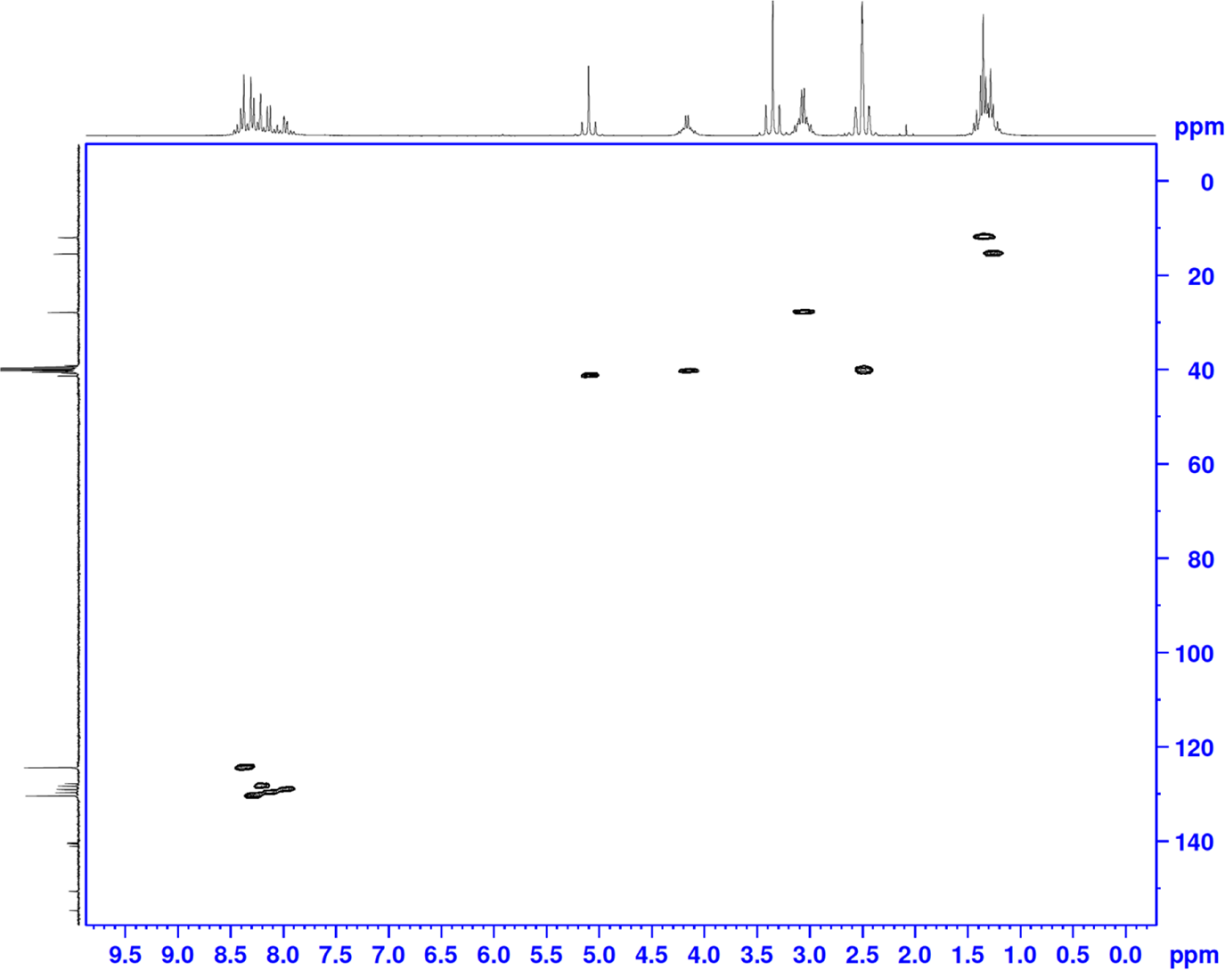
HSQC analysis of compound **5d**.

**Figure 3:**
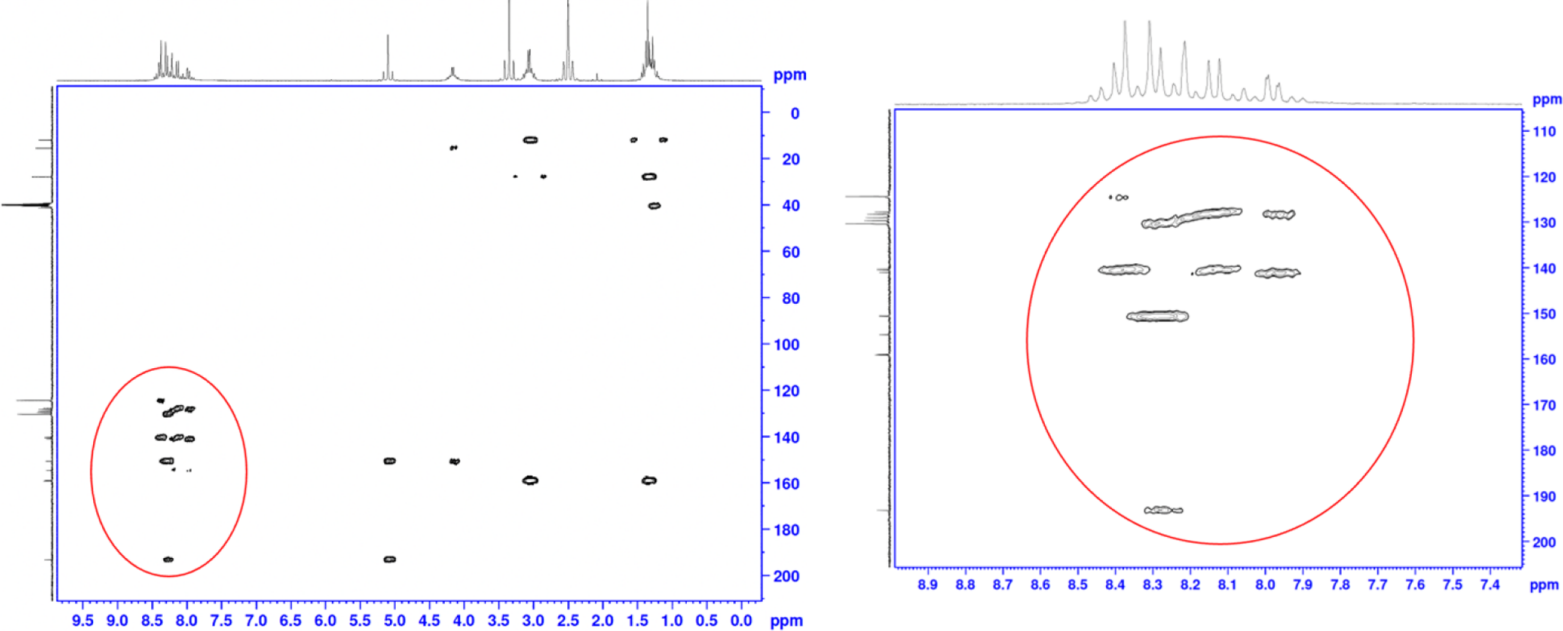
HMBC analysis of compound **5d**.

**Figure 4:**
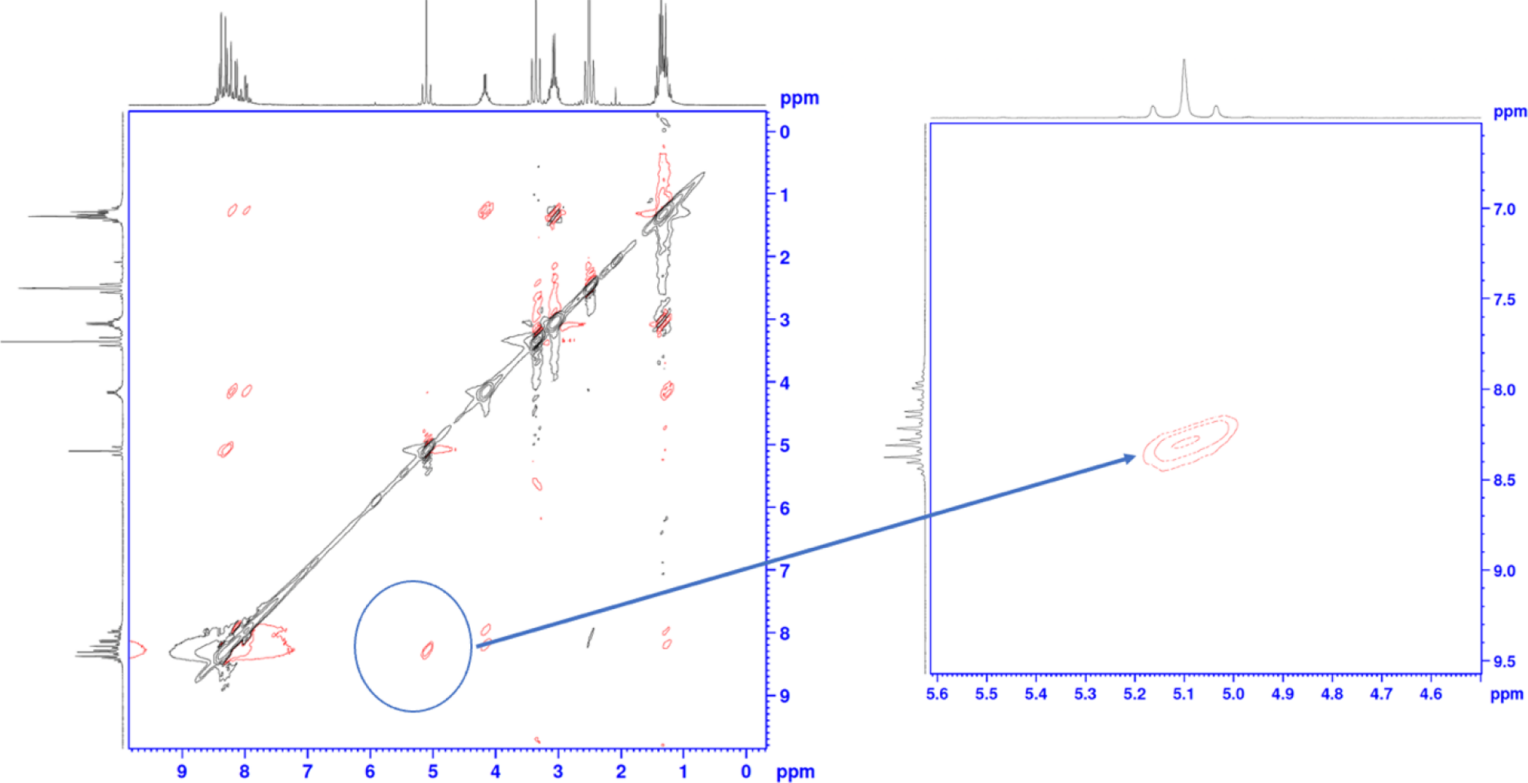
NOESY analysis of compound **5d**.

Using the HSQC technique, hydrogen NMR and carbon NMR are compared. And in this way, the interactions of hydrogen with the carbon to which it is directly bonded can be detected. Using this technique, it was determined that the carbons at the value of 124.4 ppm were the carbons in the p3 position. The carbon that comes in at 130.4 ppm belongs to the p2 carbons (Figure 2). Using the NOESY technique, where the 130.4 carbon is p2, it was determined by the interaction of the hydrogens of this carbon with the methylene group hydrogens (Figure 3). Again, using the HSQC technique, the value of 128.4 ppm is q5; the value of 129.0 is q8; and the value of 129.7 was determined to be q7. With the HMBC technique, the value of 127.7 is q4a; q8a of 140.3; p4 of 140.5; the value of 141.0 is q6; t5 of the value 150.5; p1 of the value 150.6; It was determined that the t2 value of 154.6 and the values of 158.9 and 159.1 belong to the q2 and q3 positions (Figure 4).

### 2.2 Anticandidal Activity

#### 2.2.1 Minimum Inhibitory Concentration Values

The MIC values of the compounds against *Candida* spp. range from 2 to 128 μg/mL, as shown in Table 2. Microbial growth was unaffected by the solvents (DMSO and distilled water) utilized to prepare the chemical stock solutions. Among the compounds used in the study, the strongest MIC effect was seen in the **5d** component for four *Candida* stains. Compound **5d** was outperformed fluconazole as a control for the *Candida krusei* strain in terms of antifungal activity. The obtained results are presented in Table 2.

**Table 2:** Anticandidal Activity of Synthesized Compounds (**5a**–**5k** and **7a**–**7f**) and Standard Drug (Fluconazole) (MIC_90_: μg/mL)

comp.	*C. albicans* ATCC 10231	*C. glabrata* ATCC 2951	*C. tropicalis* ATCC 750	*C. krusei* ATCC 34135
**5a**	64	64	64	64
**5b**	64	64	64	64
**5c**	128	128	128	128
**5d**	4	2	4	2
**5e**	64	128	64	128
**5f**	64	64	64	64
**5g**	64	64	128	128
**5h**	64	64	64	64
**5i**	64	128	64	128
**5j**	32	64	64	128
**5k**	32	64	128	128
**7a**	64	64	64	64
**7b**	64	128	64	128
**7c**	32	64	64	128
**7d**	32	128	32	64
**7e**	64	64	64	64
**7f**	64	128	64	128
fluconazole	0.5	2	4	16

Compound **5d** showed activity against *Candida albicans* with a value of MIC_90_ = 4 μg/mL. Fluconazole, the reference drug against the same *Candida* species, showed activity with a MIC_90_ = 0.5 μg/mL. Compound **5d** showed activity against *Candida glabrata* with a value of MIC_90_ = 2 μg/mL. Fluconazole, the reference drug against the same *Candida* species, showed activity with a value of MIC_90_ = 2 μg/mL. In other words, it showed the same activity as fluconazole against *C. glabrata*. Compound **5d** showed activity against *Candida tropicalis* with a value of MIC_90_ = 4 μg/mL. Fluconazole, the reference drug against the same *Candida* species, showed activity with MIC_90_ = 4 μg/mL. Compound **5d** showed activity against *C. krusei* with a value of MIC_90_ = 2 μg/mL. Fluconazole, the reference drug against the same *Candida* species, showed activity with a MIC_90_ = 16 μg/mL. That is, compound **5d** showed 8 times more activity compared to the reference drug.

When the structure of the compounds is examined, the compound contains an amide group in the **7a**–**7f** structure. Compounds **5a**–**5j** contain a ketone group. This difference did not make a significant difference in terms of activity. When the structure of the **5d** (active compound) is examined, the nitro substituent in the fourth position is remarkable. The inclusion of this substituent in the structure significantly increased the activity. The interaction of this group with the enzyme active site was investigated by *in silico* methods and presented in Sections 2.3 and 2.4.

#### 2.2.2 Antibiofilm Activity against Candida Species

Structured microbial populations that are connected to a surface and enclosed in an extracellular matrix that they have formed themselves are known as biofilms. A crucial aspect of infection is that *Candida* spp. can attach to and create biofilms on biological surfaces and implanted medical devices. Compared to planktonic cells, microbial biofilms are considerably more resistant to several antimicrobial treatments because they serve as protective reservoirs for the bacteria.^25^ In the present study, we evaluated the ability of the synthesized compounds to reduce the formation of fungal mature biofilms.

*Candida* isolates were exposed to three different dilutions (2 × MIC, MIC, and 1/2 × MIC) of each the compounds to assess its antibiofilm activity using the crystal violet method. The highest biofilm inhibition rates were determined at 2 × MIC values for all compounds. The compound **5d** with the lowest MIC value showed the highest biofilm inhibition rate on *C. tropicalis* at 70%–77%. Biofilm percent inhibitions results are presented in Table 3. In our study, it was found that as the MIC value decreased, the percentage of mature biofilm inhibition also decreased. When the mature biofilm inhibition percentages of the *Candida* strains of the synthesized compounds were compared, it was determined that they showed the lowest biofilm inhibition effect on *C. glabrata.* The outcomes demonstrated a dose-dependent relationship between the compounds and antibiofilm activity.

**Table 3:** Biofilm Percent Inhibitions Results of Synthesized Compounds (**5a-5 K** and **7a-7f**) and Standart Drug (Fluconazole)

	*C. albicans* ATCC 10231			*C. glabrata* ATCC 2951			*C. tropicalis* ATCC 750			*C. krusei* ATCC 34135		
comp.	2 × MIC	MIC	1/2 × MIC	2 × MIC	MIC	1/2 × MIC	2 × MIC	MIC	1/2 × MIC	2 × MIC	MIC	1/2 × MIC
**5a**	66	61	47	30	29	19	71	74	64	79	73	69
**5b**	58	52	30	41	30	21	72	70	66	75	66	64
**5c**	59	55	40	33	29	25	70	71	67	77	69	67
**5d**	61	50	36	33	26	14	72	70	68	77	74	64
**5e**	63	43	29	33	27	22	77	74	70	74	70	66
**5f**	56	36	25	29	25	15	73	72	67	77	71	65
**5g**	61	48	36	36	31	17	72	71	67	72	64	62
**5h**	61	50	36	33	26	14	72	70	68	77	74	64
**5i**	63	45	39	38	34	22	73	73	71	71	65	61
**5j**	61	41	28	37	29	22	70	70	71	77	79	78
**5k**	57	46	38	40	22	25	72	68	67	75	72	68
**7a**	50	51	28	27	26	21	70	68	70	73	70	68
**7b**	49	43	41	25	29	26	69	64	64	70	68	63
**7c**	43	41	38	44	37	29	71	66	61	77	79	77
**7d**	46	40	33	37	38	26	71	69	69	69	66	61
**7e**	50	45	30	43	31	20	71	68	67	65	63	61
**7f**	59	44	30	45	39	38	69	68	67	73	69	66
fluconazole	67	64	62	84	80	79	77	76	70	78	76	60

### 2.3 Molecular Docking

For docking studies, it was carried out with the most active derivative obtained because of *in vitro* activity. Docking studies were performed both against the 14-α-demethylases (CYP51) (1EA1).^26^ Docking studies were carried out using compound **5d** with anticandidal activity values below MIC_90_ = 2 μg/ml.

**Figure 5:**
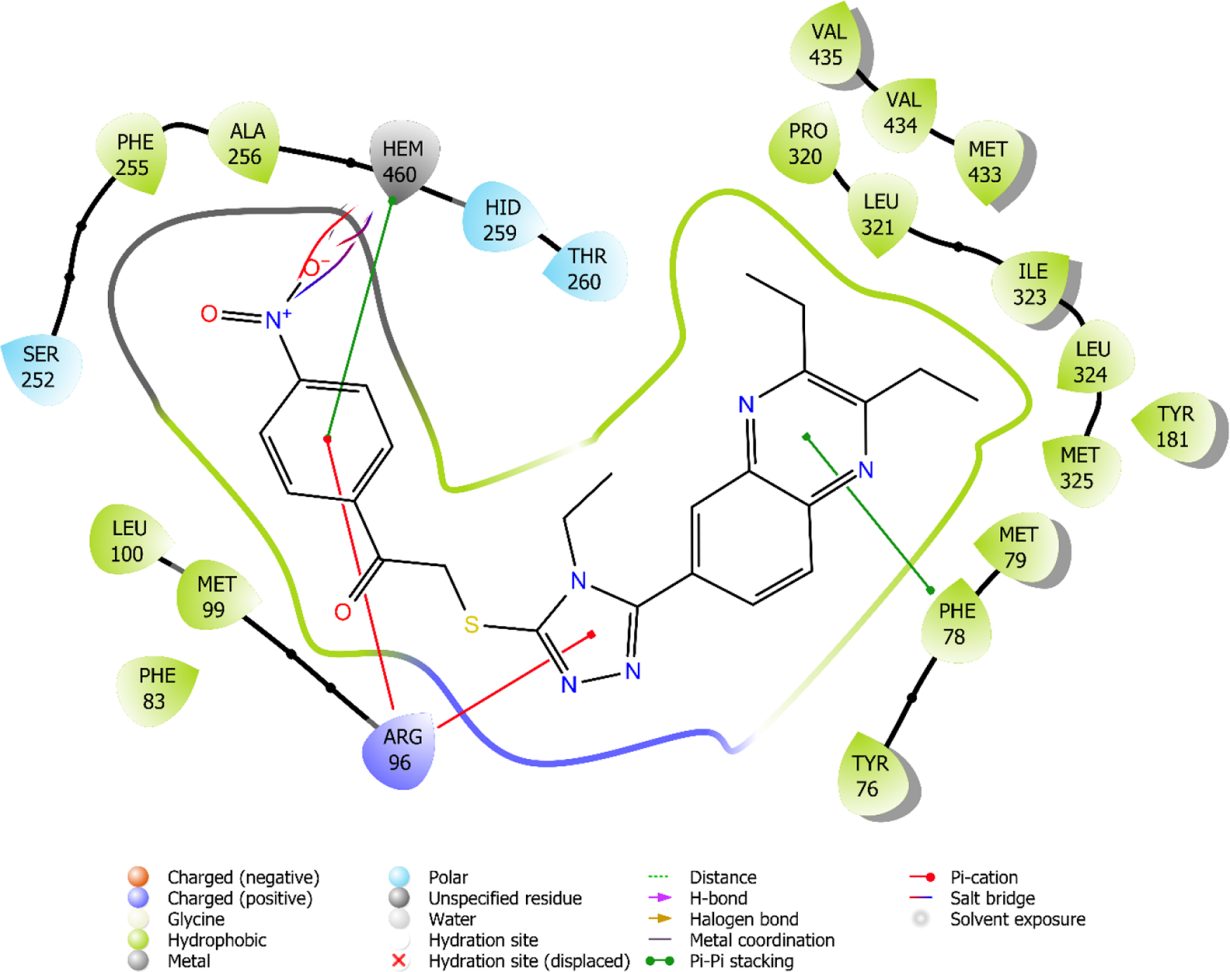
2D interaction of compound **5d** at binding region (PDB ID: 1EA1).

When Figure 5 is examined, two-dimensional interactions of compound **5d** in the enzyme active site are seen. Here, it is seen that the NO_2_ group makes a cation−π bond in addition to the triple salt bridge with HEM460 in the enzyme active site. This interaction clearly revealed the contribution of the NO_2_ group to the activity. In addition, the phenyl ring also establishes a π–π interaction with HEM460. The triazole ring and the phenyl ring formed a cation−π bond with the amino group of Arg96. Finally, the quinoxaline ring formed a π–π interaction with the phenyl group of Phe78.

**Figure 6:**
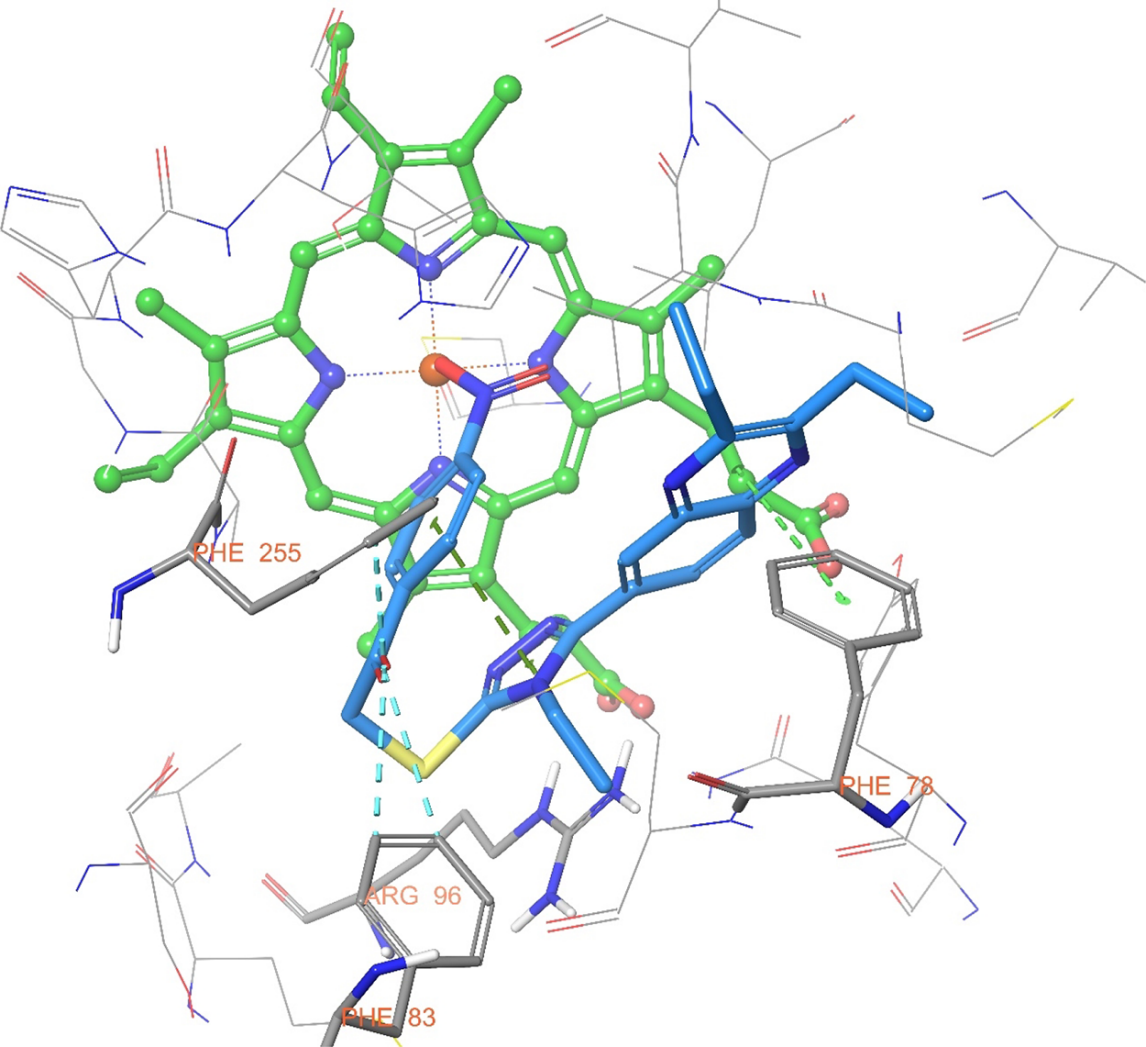
3D interaction of compound **5d** at binding region (PDB ID: 1EA1).

When Figure 6 is examined, the three-dimensional interaction of compound **5d** with the enzyme active site will be seen. In addition to the interactions displayed with the two-dimensional pose, aromatic hydrogen bonds are seen in this pose. The carbonyl group of compound **5d** formed three aromatic hydrogen bonds with the phenyl groups of Phe255 and Phe83.

### 2.4 Molecular Dynamics Simulation

Docking studies do not give a clear answer about the dynamic behavior and stability of proteins and protein-ligand complexes. For this purpose, the MD simulation method is often used. In the present study, to estimate the stability of the docking complex made between the promising molecule (**5d**) and 14-α-demethylases (CYP51) (1EA1), were taken into consideration for the 100 ns MD simulation study in an explicit hydration environment.

**Figure 7:**
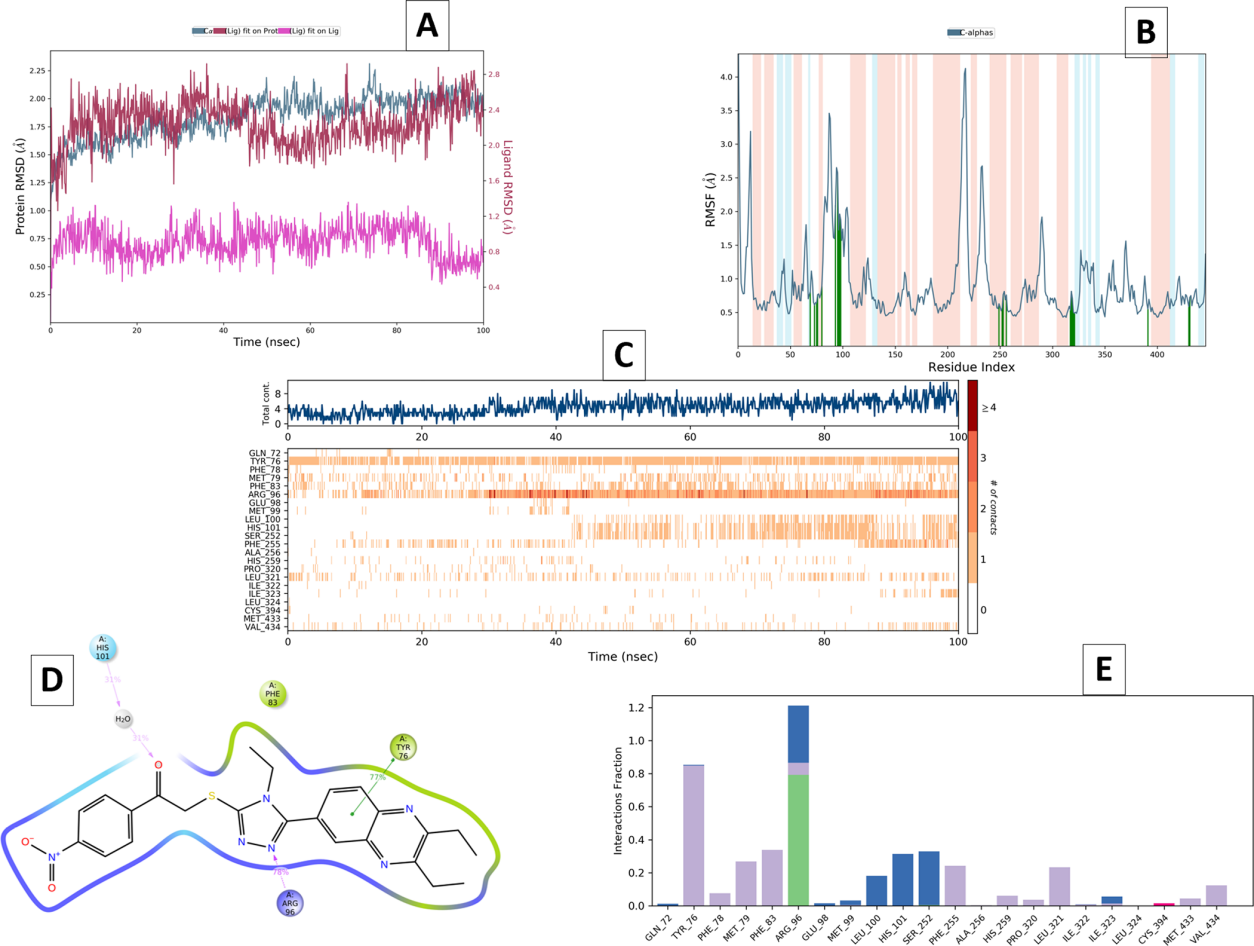
MD simulation analysis of compound **5d** in complex 14-α-demethylases enzyme (PDB ID: 1EA1). (A) RMSD (Protein RMSD is shown in gray while RMSD of compound **5**d are shown in red). (B) Protein RMSF. (C) Amino acid interaction histogram. (D) 2D interaction diagram and (E) protein–ligand contact analysis of MD trajectory.

In Figure 7, the results are for the compound **5d**-14-α-demethylases enzyme complex. The RMSD and RMSF parameters are used to measure the stability of the model created during the simulation period. Figure 7a presents the RMSD parameter plot. In the RMSD graph we obtained, it ranged from 1.25 to 2.25 Å and was fixed here. Since these values are between 1–3 Å, it would be correct to say that our complex maintains its stability. It is seen that the stability shows a slight fluctuation at 40 ns. However, after a short time after this fluctuation, it becomes stable again. Re-establishment of stability may be due to bonds with Leu100, His101 and Ser252. A similar fluctuation is observed at 682 ns. But this lasts for such a short time that it is negligible. It would be correct to evaluate the reason for the fluctuation here as the breaking of the bonds made with the iron of HEM460. The iron in the middle of HEM460 forms either a salt bridge with a nitro group, a cation−π bond with a triazole, or a cation−π bond with a phenyl ring. None of these bonds are present at 682 ns. However, as of 683 ns, these bonds continue, and stability is provided. The stability established in 880 ns continues until the end of its dynamic studies. This stabilization is thought to be the result of hydrophobic interactions with Phe255 (Figure 7c).

It is known that individual amino acids play a very important role in the stability of the protein-ligand complex during the MD simulation study. The RMSF parameter is important for stability and is used to interpret the conformational changes of individual residues (Figure 7b). α-Helix regions are red in the RMSF plot; β-banded regions are represented by a blue background; the white background represents the loop region. The contribution of contacting residues between each protein chain and ligand is indicated by vertical green lines on the *x* axis of the plot.^27−29^

As per RMSF plot, compound **5d** contacted 21 amino acids of 14-α-demethylases protein, namely, Gln72 (0.81 Å), Ala75 (0.65 Å), Phe78 (0.64 Å), Met79 (0.77 Å), Phe83 (0.89 Å), Arg96 (2.09 Å), Glu98 (2.58 Å), Met99 (1.73 Å), His101 (1.88 Å), Ser252 (0.63 Å), Phe255 (0.57 Å), Ala256 (0.77 Å), His259 (0.76 Å), Pro320 (0.54 Å), Leu321 (0.81 Å), Ile322 (0.71 Å), Ile323 (0.51 Å), Leu324 (0.49 Å), Cys394 (0.55 Å), Met433 (0.59 Å), Val434 (0.72 Å).

By watching the MD simulation video, aromatic hydrogen bonds were determined for 100 ns seconds. Accordingly, the aromatic hydrogen bonds formed can be listed as follows. Between the carbonyl group of compound **5d** and the phenyl group of Phe83; between NO_2_ group of compound **5d** and imidazole group of Hid259; between the phenyl group of compound **5d** and the Ala256 carbonyl; between the phenyl group of compound **5d** and the Phe255 carbonyl; between the phenyl group of compound **5d** and the carbonyl group of Hid259 and between the carbonyl group of compound **5d** and the phenyl group of Phe255.

## 3 Conclusions

Among the antifungal drug groups, azole group antifungals have a very important place. This group of drugs is frequently used in the clinic. However, resistance to the drug, especially in long-term treatments, keeps the need for new azole antifungals constantly. Azole antifungals inhibit 14-α-demethylases enzyme and inhibit ergosterol synthesis necessary for fungal cell wall construction. The azole group heterocyclic ring is essential for its interaction with hemoglobin in the enzyme active site. This is exactly the purpose of the synthesis of new triazole derivatives within the scope of this study. In this study, 17 new triazole derivatives were synthesized. Structure determinations of the compounds were made using ^1^H-NMR, ^13^C-NMR and HRMS techniques. In addition, structure determination was validated for compound **5d** using the 2D-NMR technique. In addition to the antifungal activities of the compounds, the percent biofilm inhibition values were tested on *Candida* strains by *in vitro* methods. As a result of activity studies, compound **5d** exhibited a similar antifungal potential with fluconazole. It was even 8 times more active on *C. krusei* than fluconazole. This promising result has moved the study forward. The obtained *in silico* studies also confirm the activity studies. Molecular docking and then molecular dynamics studies were carried out as *in silico* studies. Dynamic studies showed that the interaction with hemoglobin iron, which is necessary for activity, is carried out from three points of our molecule. These structures are nitro substituent, triazole ring and phenyl ring. Especially the nitro group and the triazole ring showed a significant interaction rate. Therefore, the importance of both the triazole ring and the nitro substituent for the activity is clear. The bulky quinoxaline ring showed a continuous and strong interaction with the Tyr76 amino acid for 100 ns. Although this interaction may not be of primary importance for activity, it is thought to play an important role in maintaining the stability of the compound. In future studies, it is planned to develop the compound by keeping these structures constant.

## 4 Experimental Section

### 4.1 Chemistry

Purchase processes and analysis methods of all commercial preparations were carried out as previously reported.^29^

#### 4.1.1 Synthesis of Methyl 2,3-Diethylquinoxaline-6-carboxylate (**1**)

Methyl 3,4-diaminobenzoate (6 g, 0.036 mol) and hexane-3,4-dione (4.104 g, 0.036 mol) were refluxed in EtOH (30 mL). At the end of the reaction, the precipitated product was filtered, washed with cooled EtOH, dried, and crystallized from ethanol.

#### 4.1.2 Synthesis of 2,3-Diethylquinoxaline-6-carbohydrazide (**2**)

Methyl 2,3-diethylquinoxaline-6-carboxylate (7.9056 g, 0.032 mol) was dissolved in absolute EtOH. Then the excess of hydrazine hydrate dissolved in EtOH (absolute) at different vessel. The hydrazine hydrate solution was added in reaction medium as portions. After the add process is complete, the reaction mixture was refluxed at 2 h. At the end of the reaction, the precipitated product was filtered, washed with cooled EtOH, dried, and crystallized from ethanol.

#### 4.1.3 Synthesis of 2-(2,3-Diethylquinoxaline-6-carbonyl)-*N*-ethylhydrazine-1-carbothioamide (**3**)

2,3-Diethylquinoxaline-6-carbohydrazide (6.6368 g, 0.027 mol) and isothiocyanatoethane (2.3528 g, 0.027 mol) were dissolved in absolute EtOH. Then, the reaction mixture was refluxed at 5 h. At the end of the reaction, the precipitated product was filtered, washed with cooled EtOH, dried, and crystallized from ethanol.

#### 4.1.4 Synthesis of 5-(2,3-Diethylquinoxalin-6-yl)-4-ethyl-4*H*-1,2,4-triazole-3-thiol (**4**)

2-(2,3-Diethylquinoxaline-6-carbonyl)-*N*-ethylhydrazine-1-carbothioamide (7.1591 g, 0.022 mol) was dissolved in absolute EtOH in presence of NaOH. Then, the reaction mixture was refluxed at 5 h. At the end of the reaction, the reaction mixture was poured in iced water. The pH of the reaction content was adjusted to pH = 2. For this adjustment, 20% HCl was used as the acidic agent. Then, the precipitated product was filtered, washed with distilled water, dried, and crystallized from ethanol.

#### 4.1.5 Synthesis of the Target Compounds (**5a–5k**)

5-(2,3-Diethylquinoxalin-6-yl)-4-ethyl-4*H*-1,2,4-triazole-3-thiol (0.3 g, 0.001 mol) and appropriate 2-brmoacetophenone (0.002 mol) were stirred in acetone (20 mL) in presence of potassium carbonate for 8 h. At the end of the reaction, the acetone was evaporated. The residue product washed with distilled water, dried, and crystallized from ethanol.

##### 4.1.5.1 2-((5-(2,3-Diethylquinoxalin-6-yl)-4-ethyl-4*H*-1,2,4-triazol-3-yl)thio)-1-(*p*-tolyl)ethan-1-one (**5a**)

Yield: 88%, cream. M.P.: 161.3–162.7 °C. IR (cm ^–1^ band): 2976 (C-H), 1668 (C=O), 817. ^1^H-NMR (300 MHz, DMSO-*d*_6_): δ = 1.27 (3H, t, *J* = 7.2 Hz, -CH_3_), 1.33–1.38 (6H, m, -CH_3_), 2.40 (3H, s, -CH_3_), 3.02–3.10 (4H, m, -CH_2_-), 4.15 (2H, q, *J* = 7.3 Hz, -CH_2_-), 4.99 (2H, s, -CH_2_-), 7.37 (2H, d, *J* = 7.9 Hz, 1,4-disubstituted benzene), 7.94 (2H, d, *J* = 8.1 Hz, 1,4-disubstituted benzene), 7.99 (1H, d, *J* = 1.9 Hz, quinoxalin), 8.14 (1H, d, *J* = 8.6 Hz, quinoxalin), 8.21 (1H, d, *J* = 1.8 Hz, quinoxalin). ^13^C-NMR (75 MHz, DMSO-*d*_6_): δ =11.96, 12.01, 15.48, 21.70, 27.86, 40.34, 41.23, 127.81, 128.22, 129.01, 129.04, 129.65, 129.85, 133.25, 140.32, 141.02, 144.81, 150.80, 154.52, 158.95, 159.11, 193.22. HRMS (*m*/*z*): [M + H]^+^ calcd for C_25_H_27_N_5_OS: 446.2009; found: 446.1991.

##### 4.1.5.2 2-((5-(2,3-Diethylquinoxalin-6-yl)-4-ethyl-4*H*-1,2,4-triazol-3-yl)thio)-1-(4-methoxyphenyl)ethan-1-one (**5b**)

Yield: 90%, cream. M.P.: 142.9–144.0 °C. IR (cm ^–1^ band): 2972 (C–H), 1660 (C=O), 817. ^1^H-NMR (300 MHz, DMSO-*d*_6_): δ = 1.27 (3H, t, *J* = 7.2 Hz, -CH_3_), 1.33–1.39 (6H, m, -CH_3_), 3.03–3.11 (4H, m, -CH_2_-), 3.87 (3H, s, -OCH_3_), 4.16 (2H, q, *J* = 7.1 Hz, -CH_2_-), 4.97 (2H, s, -CH_2_-), 7.09 (2H, d, *J* = 8.9 Hz, 1,4-disubstituted benzene), 7.98 (1H, dd, *J_1_* = 1.9 Hz, *J_2_* = 8.6 Hz, quinoxalin), 8.03 (2H, d, *J* = 8.9 Hz, 1,4-disubstituted benzene), 8.14 (1H, d, *J* = 8.6 Hz, quinoxalin), 8.21 (1H, d, *J* = 1.8 Hz, quinoxalin). ^13^C-NMR (75 MHz, DMSO-*d*_6_): δ =11.95, 12.01, 27.85, 40.35, 56.14, 114.52, 127.82, 128.20, 128.59, 128.99, 129.65, 131.35, 140.32, 141.02, 150.88, 154.50, 158.95, 159.10, 164.06, 192.03. HRMS (*m*/*z*): [M + H]^+^ calcd for C_25_H_27_N_5_O_2_S: 462.1958; found: 462.1944.

##### 4.1.5.3 4-(2-((5-(2,3-Diethylquinoxalin-6-yl)-4-ethyl-4H-1,2,4-triazol-3-yl)thio)acetyl)benzonitrile (**5c**)

Yield: 85%, beige. M.P.: 153.1–154.6 °C. IR (cm ^–1^ band): 2981 (C–H), 2227 (-CN), 1680 (C=O), 829. ^1^H-NMR (300 MHz, DMSO-*d*_6_): δ = 1.27 (3H, t, *J* = 7.2 Hz, -CH_3_), 1.33–1.38 (6H, m, -CH_3_), 3.03–3.11 (4H, m, -CH_2_-), 4.16 (2H, q, *J* = 7.1 Hz, -CH_2_-), 5.06 (2H, s, -CH_2_-), 7.98 (1H, dd, *J*_1_ = 1.9 Hz, *J*_2_ = 8.6 Hz, quinoxalin), 8.06 (2H, d, *J* = 8.6 Hz, 1,4-disubstituted benzene), 8.14 (1H, d, *J* = 8.6 Hz, quinoxalin), 8.19–8.22 (3H, m, quinoxalin+1,4-disubstituted benzene). ^13^C-NMR (75 MHz, DMSO-*d*_6_): δ =11.97, 12.03, 15.45, 27.86, 40.40, 41.14, 116.04, 118.58, 127.76, 128.26, 129.00, 129.55, 129.67, 133.35, 139.08, 140.33, 141.04, 150.53, 154.61, 158.98, 159.15, 193.37. HRMS (*m*/*z*): [M + H]^+^ calcd for C_25_H_24_N_6_OS: 457.1805; found: 457.1800.

##### 4.1.5.4 2-((5-(2,3-Diethylquinoxalin-6-yl)-4-ethyl-4*H*-1,2,4-triazol-3-yl)thio)-1-(4-nitrophenyl)ethan-1-one (**5d**)

Yield: 79%, brown. M.P.: 144.0–145.9 °C. IR (cm ^–1^ band): 2970 (C–H), 1681 (C=O), 1527 (NO_2_), 1342 (NO_2_), 854.^1^H-NMR (300 MHz, DMSO-*d*_6_): δ = 1.28 (3H, t, *J* = 7.2 Hz, -CH_3_), 1.33–1.38 (6H, m, -CH_3_), 3.03–3.10 (4H, m, -CH_2_-), 4.16 (2H, q, *J* = 7.5 Hz, -CH_2_-), 5.09 (2H, s, -CH_2_-), 7.98 (1H, dd, *J_1_* = 1.9 Hz, *J_2_* = 8.7 Hz, quinoxalin), 8.13 (1H, d, *J* = 8.7 Hz, quinoxalin), 8.21 (1H, d, *J* = 1.8 Hz, quinoxalin), 8.29 (2H, d, *J* = 8.9 Hz, 1,4-disubstituted benzene), 8.38 (2H, d, *J* = 8.9 Hz, 1,4-disubstituted benzene). ^13^C-NMR (75 MHz, DMSO-*d*_6_): δ =11.96, 12.02, 15.46, 27.85, 41.32, 124.39, 127.74, 128.27, 129.00, 129.67, 130.38, 140.32, 140.51, 141.03, 150.52, 150.62, 154.63, 158.98, 159.14, 193.20. HRMS (*m*/*z*): [M + H]^+^ calcd for C_24_H_24_N_6_O_3_S: 477.1703; found: 477.1691.

##### 4.1.5.5 2-((5-(2,3-Diethylquinoxalin-6-yl)-4-ethyl-4*H*-1,2,4-triazol-3-yl)thio)-1-(4-fluorophenyl)ethan-1-one (**5e**)

Yield: 77%, cream. M.P.: 150.4–151.8 °C. IR (cm ^–1^ band): 2978 (C–H), 1670 (C=O), 835. ^1^H-NMR (300 MHz, DMSO-*d*_6_): δ = 1.28 (3H, t, *J* = 7.2 Hz, -CH_3_), 1.33–1.38 (6H, m, -CH_3_), 3.03–3.11 (4H, m, -CH_2_-), 4.16 (2H, q, *J* = 7.1 Hz, -CH_2_-), 5.02 (2H, s, -CH_2_-), 7.37–7.44 (2H, m, 1,4-disubstituted benzene), 7.98 (1H, dd, *J*_1_ = 1.9 Hz, *J*_2_ = 8.6 Hz, quinoxalin), 8.12–8.17 (3H, m, quinoxalin + 1,4-disubstituted benzene), 8.22 (1H, d, *J* = 1.8 Hz, quinoxalin). ^13^C-NMR (75 MHz, DMSO-*d*_6_): δ =11.96, 12.02, 15.47, 27.85, 40.37, 41.11, 116.39 (d, *J* = 21.7 Hz), 127.79, 128.24, 129.00, 129.66, 132.02 (d, *J* = 9.4 Hz), 132.53, 140.32, 141.03, 150.73, 154.55, 158.97, 159.12, 165.79 (d, *J* = 250.8 Hz), 192.37. HRMS (*m*/*z*): [M + H]^+^ calcd for C_24_H_24_N_5_OFS: 450.1758; found: 450.1742.

##### 4.1.5.6 1-(4-Chlorophenyl)-2-((5-(2,3-diethylquinoxalin-6-yl)-4-ethyl-4*H*-1,2,4-triazol-3-yl)thio)ethan-1-one (**5f**)

Yield: 80%, cream. M.P.: 184.5–185.9 °C. IR (cm ^–1^ band): 2978 (C–H), 1670 (C=O), 827. ^1^H-NMR (300 MHz, DMSO-*d*_6_): δ = 1.27 (3H, t, *J* = 7.2 Hz, -CH_3_), 1.33–1.38 (6H, m, -CH_3_), 3.03–3.10 (4H, m, -CH_2_-), 4.16 (2H, q, *J* = 7.1 Hz, -CH_2_-), 5.02 (2H, s, -CH_2_-), 7.65 (2H, d, *J* = 8.6 Hz, 1,4-disubstituted benzene), 7.98 (1H, dd, *J_1_* = 1.9 Hz, *J_2_* = 8.6 Hz, quinoxalin), 8.07 (2H, d, *J* = 8.6 Hz, 1,4-disubstituted benzene), 8.14 (1H, d, *J* = 8.6 Hz, quinoxalin), 8.22 (1H, d, *J* = 1.8 Hz, quinoxalin). ^13^C-NMR (75 MHz, DMSO-*d*_6_): δ =11.97, 12.02, 15.47, 27.87, 40.24, 41.09, 128.22, 128.26, 129.01, 129.44, 129.67, 130.86, 130.87, 140.33, 141.10, 146.69, 150.66, 154.65, 156.11, 159.18, 192.87. HRMS (*m*/*z*): [M + H]^+^ calcd for C_24_H_24_N_5_OSCl: 466.1463; found: 466.1458.

##### 4.1.5.7 1-(4-Bromophenyl)-2-((5-(2,3-diethylquinoxalin-6-yl)-4-ethyl-4*H*-1,2,4-triazol-3-yl)thio)ethan-1-one (**5g**)

Yield: 81%, pink. M.P.: 132.3–134.1 °C. IR (cm ^–1^ band): 2974 (C–H), 1670 (C=O), 823. ^1^H-NMR (300 MHz, DMSO-*d*_6_): δ = 1.27 (3H, t, *J* = 7.2 Hz, -CH_3_), 1.33–1.38 (6H, m, -CH_3_), 3.03–3.10 (4H, m, -CH_2_-), 4.16 (2H, q, *J* = 7.0 Hz, -CH_2_-), 5.03 (2H, s, -CH_2_-), 7.79 (2H, d, *J* = 8.6 Hz, 1,4-disubstituted benzene), 7.98–8.01 (3H, m, quinoxalin + 1,4-disubstituted benzene), 8.14 (1H, d, *J* = 8.6 Hz, quinoxalin), 8.22 (1H, d, *J* = 1.8 Hz, quinoxalin). ^13^C-NMR (75 MHz, DMSO-*d*_6_): δ =11.08, 12.01, 15.43, 27.91, 39.09, 40.28, 41.14, 128.43, 128.98, 129.89, 130.91, 131.23, 131.96, 132.40, 133.55, 134.83, 140.28, 141.12, 150.85, 154.53, 159.26, 192.85. HRMS (*m*/*z*): [M + H]^+^ calcd for C_24_H_24_N_5_OSBr: 510.0958; found: 510.0952.

##### 4.1.5.8 2-((5-(2,3-Diethylquinoxalin-6-yl)-4-ethyl-4*H*-1,2,4-triazol-3-yl)thio)-1-(2,4-dimethylphenyl)ethan-1-one (**5h**)

Yield: 87%, orange. M.P.: 122.5–123.8 °C. IR (cm ^–1^ band): 2976 (C–H), 1674 (C=O), 839. ^1^H-NMR (300 MHz, DMSO-*d*_6_): δ = 1.26 (3H, t, *J* = 7.2 Hz, -CH_3_), 1.33–1.38 (6H, m, -CH_3_), 2.33 (3H, s, -CH3), 2.39 (3H, s, -CH3), 3.03–3.10 (4H, m, -CH_2_-), 4.12 (2H, q, *J* = 7.0 Hz, -CH_2_-), 4.89 (2H, s, -CH_2_-), 7.15–7.18 (2H, m, 1,2,4-trisubstituted benzene), 7.86 (1H, d, *J* = 7.8 Hz, 1,2,4-trisubstituted benzene), 7.98 (1H, dd, *J_1_* = 1.9 Hz, *J_2_* = 8.5 Hz, quinoxalin), 8.14 (1H, d, *J* = 8.7 Hz, quinoxalin), 8.20 (1H, d, *J* = 1.9 Hz, quinoxalin). ^13^C-NMR (75 MHz, DMSO-*d*_6_): δ = 11.97, 12.02, 15.44, 21.40, 27.85, 43.05, 126.89, 127.82, 128.21, 129.01, 129.66, 130.23, 132.95, 133.54, 138.67, 140.33, 141.02, 142.71, 150.86, 154.50, 158.97, 159.13, 196.35. HRMS (*m*/*z*): [M + H]^+^ calcd for C_26_H_29_N_5_OS: 460.2166; found: 460.2159.

##### 4.1.5.9 2-((5-(2,3-Diethylquinoxalin-6-yl)-4-ethyl-4*H*-1,2,4-triazol-3-yl)thio)-1-(2,4-difluorophenyl)ethan-1-one (**5i**)

Yield: 77%, beige. M.P.: 130.3–131.7 °C. IR (cm ^–1^ band): 2976 (C–H), 1678 (C=O), 821. ^1^H-NMR (300 MHz, DMSO-*d*_6_): δ = 1.27 (3H, t, *J* = 7.2 Hz, -CH_3_), 1.33–1.38 (6H, m, -CH_3_), 3.03–3.11 (4H, m, -CH_2_-), 4.15 (2H, q, *J* = 6.8 Hz, -CH_2_-), 4.91 (2H, d, J = 2.5 Hz, -CH_2_-), 7.26–7.32 (1H, m, 1,2,4-trisubstituted benzene), 7.47–7.54 (1H, m, 1,2,4-trisubstituted benzene), 7.96–8.00 (1H, m, quinoxalin), 8.02–8.07 (1H, m, 1,2,4-trisubstituted benzene), 8.14 (1H, d, *J* = 8.6 Hz, quinoxalin), 8.22 (1H, d, *J* = 1.7 Hz, quinoxalin). ^13^C-NMR (75 MHz, DMSO-*d*_6_): δ =11.97, 12.03, 15.43, 27.85, 40.35, 44.27 (d, *J* = 7.8 Hz), 105.82 (t, *J* = 25.9 Hz), 113.07 (d, *J* = 22.3 Hz), 122.02 (d, *J* = 40.1 Hz), 127.77, 128.27, 129.03, 129.66, 133.43, 140.32, 141.03, 150.66, 154.58, 159.05 (d, *J* = 11.9 Hz), 165.79 (d, *J* = 251.8 Hz), 165.97 (d, *J* = 250.3 Hz), 190.30. HRMS (*m*/*z*): [M + H]^+^ calcd for C_24_H_23_N_5_OF_2_S: 468.1664; found: 468.1651.

##### 4.1.5.10 1-(2,4-Dichlorophenyl)-2-((5-(2,3-diethylquinoxalin-6-yl)-4-ethyl-4*H*-1,2,4-triazol-3-yl)thio)ethan-1-one (**5j**)

Yield: 79%, orange. M.P.: 65.9–66.8 °C. IR (cm ^–1^ band): 2976 (C–H), 1685 (C=O), 813. ^1^H-NMR (300 MHz, DMSO-*d*_6_): δ = 1.24 (3H, t, *J* = 7.3 Hz, -CH_3_), 1.31–1.37 (6H, m, -CH_3_), 3.03–3.08 (4H, m, -CH_2_-), 4.11 (2H, q, *J* = 7.2 Hz, -CH_2_-), 4.87 (2H, s, -CH_2_-), 7.60 (1H, dd, *J_1_* = 3.5 Hz, *J_2_* = 8.4 Hz, 1,2,4-trisubstituted benzene), 7.76 (1H, d, *J* = 1.9 Hz, 1,2,4-trisubstituted benzene), 7.91 (1H, d, *J* = 8.4 Hz, 1,2,4-trisubstituted benzene), 7.97 (1H, dd, *J_1_* = 1.9 Hz, *J_2_* = 8.6 Hz, quinoxalin), 8.12 (1H, d, *J* = 8.6 Hz, quinoxalin), 8.20 (1H, d, *J* = 1.8 Hz, quinoxalin). ^13^C-NMR (75 MHz, DMSO-*d*_6_): δ =11.97, 12.01, 15.39, 27.85, 27.87, 40.34, 43.05, 127.71, 128.07, 128.27, 128.99, 129.66, 130.57, 132.01, 132.09, 135.80, 137.28, 140.32, 141.04, 150.44, 154.62, 158.97, 159.14, 194.94. HRMS (*m*/*z*): [M + H]^+^ calcd for C_24_H_23_N_5_OSCl_2_: 500.1073; found: 500.1057.

##### 4.1.5.11 1-(3,4-Dichlorophenyl)-2-((5-(2,3-diethylquinoxalin-6-yl)-4-ethyl-4H-1,2,4-triazol-3-yl)thio)ethan-1-one (**5k**)

Yield: 83%, orange. M.P.: 75.0–76.9 °C. IR (cm ^–1^ band): 2978 (C–H), 1670 (C=O), 835. ^1^H-NMR (300 MHz, DMSO-*d*_6_): δ = 1.27 (3H, t, *J* = 7.3 Hz, -CH_3_), 1.33–1.38 (6H, m, -CH_3_), 3.03–3.11 (4H, m, -CH_2_-), 4.16 (2H, q, *J* = 7.4 Hz, -CH_2_-), 5.02 (2H, s, -CH_2_-), 7.86 (1H, d, *J* = 8.4 Hz, 1,3,4-trisubstituted benzene), 7.96–8.02 (3H, m, quinoxalin+1,3,4-trisubstituted benzene), 8.14 (1H, d, *J* = 8.6 Hz, quinoxalin), 8.22 (1H, d, *J* = 1.9 Hz, quinoxalin), 8.28 (1H, d, *J* = 1.9 Hz, 1,3,4-trisubstituted benzene). ^13^C-NMR (75 MHz, DMSO-*d*_6_): δ = 11.98, 12.02, 15.47, 27.86, 40.39, 40.99, 127.77, 128.25, 128.29, 128.99, 129.67, 130.89, 130.92, 131.68, 132.38, 136.01, 137.03, 140.33, 141.04, 150.49, 154.61, 159.16, 192.25. HRMS (*m*/*z*): [M + H]^+^ calcd for C_24_H_23_N_5_OSCl_2_: 500.1073; found: 500.1059.

#### 4.1.6 Synthesis of Acetylated Anilines (**6a**–**6f**)

Aniline derivatives (0.009 mol) was dissolved in THF. Triethylamine was used as catalyst. The reaction mixture located in iced bath. The chloroacetyl chloride mixture in THF was added in reaction mixture as dropwise. At the end of the reaction, the THF was evaporated. The residue product washed with distilled water, dried, and crystallized from ethanol.

#### 4.1.7 Synthesis of Target Compounds (**7a**–**7f**)

5-(2,3-Diethylquinoxalin-6-yl)-4-ethyl-4*H*-1,2,4-triazole-3-thiol (0.3 g, 0.001 mol) and appropriate acetylated anilines (**6a**–**6f**) (0.002 mol) were stirred in acetone (20 mL) in presence of potassium carbonate for 8 h. At the end of the reaction, the acetone was evaporated. The residue product washed with distilled water, dried, and crystallized from ethanol.

##### 4.1.7.1 2-((5-(2,3-Diethylquinoxalin-6-yl)-4-ethyl-4*H*-1,2,4-triazol-3-yl)thio)-*N*-phenylacetamide (**7a**)

Yield: 81%, white. M.P.: 124.2–125.5 °C. IR (cm ^–1^ band): 3481 (N–H), 2981 (C–H), 1681 (C=O), 758, 694. ^1^H-NMR (300 MHz, DMSO-*d*_6_): δ = 1.27 (3H, t, *J* = 7.2 Hz, -CH_3_), 1.33–1.38 (6H, m, -CH_3_), 3.03–3.10 (4H, m, -CH_2_-), 4.16 (2H, q, *J* = 7.2 Hz, -CH_2_-), 4.24 (2H, s, -CH_2_-), 7.04–7.09 (1H, m, monosubstituted benzene), 7.29–7.35 (2H, m, monosubstituted benzene), 7.57–7.60 (2H, m, monosubstituted benzene), 7.98 (1H, dd, *J_1_* = 1.9 Hz, *J_2_* = 8.6 Hz, quinoxalin), 8.14 (1H, d, *J* = 8.6 Hz, quinoxalin), 8.22 (1H, d, *J* = 1.8 Hz, quinoxalin), 10.38 (1H, s, -NH). ^13^C-NMR (75 MHz, DMSO-*d*_6_): δ =11.97, 12.01, 15.49, 27.85, 27.87, 40.35, 119.56, 124.03, 127.80, 128.28, 129.02, 129.31, 129.65, 139.26, 140.32, 141.03, 150.92, 154.62, 158.97, 159.14, 166.10. HRMS (*m*/*z*): [M + H]^+^ calcd for C_24_H_26_N_6_OS: 447.1962; found: 447.1952.

##### 4.1.7.2 2-((5-(2,3-Diethylquinoxalin-6-yl)-4-ethyl-4*H*-1,2,4-triazol-3-yl)thio)-*N*-(*p*-tolyl)acetamide (**7b**)

Yield: 86%, cream. M.P.: 92.9–94.1 °C. IR (cm ^–1^ band): 3257 (N-H), 2987 (C–H), 1687 (C=O), 850. ^1^H-NMR (300 MHz, DMSO-*d*_6_): δ = 1.26 (3H, t, *J* = 7.2 Hz, -CH_3_), 1.33–1.38 (6H, m, -CH_3_), 2.25 (3H, s, -CH_3_), 3.03–3.10 (4H, m, -CH_2_-), 4.15 (2H, q, *J* = 6.9 Hz, -CH_2_-), 4.22 (2H, s, -CH_2_-), 7.12 (2H, d, *J* = 8.3 Hz, 1,4-disubstituted benzene), 7.47 (2H, d, *J* = 8.5 Hz, 1,4-disubstituted benzene), 7.98 (1H, dd, *J_1_* = 1.9 Hz, *J_2_* = 8.6 Hz, quinoxalin), 8.14 (1H, d, *J* = 8.5 Hz, quinoxalin), 8.21 (1H, d, *J* = 1.9 Hz, quinoxalin), 10.29 (1H, s, -NH). ^13^C-NMR (75 MHz, DMSO-*d*_6_): δ =11.97, 12.02, 15.48, 27.85, 27.88, 37.89, 119.47, 126.67, 126.72, 127.75, 128.28, 129.03, 129.66, 140.31, 141.03, 142.88, 150.83, 154.66, 158.99, 159.16, 166.87. HRMS (*m*/*z*): [M + H]^+^ calcd for C_25_H_28_N_6_OS: 461.2118; found: 461.2105.

##### 4.1.7.3 2-((5-(2,3-Diethylquinoxalin-6-yl)-4-ethyl-4*H*-1,2,4-triazol-3-yl)thio)-*N*-(4-methoxyphenyl)acetamide (**7c**)

Yield: 75%, white. M.P.: 149.3–151.1 °C. IR (cm ^–1^ band): 3496 (N–H), 2976 (C–H), 1678 (C=O), 840. ^1^H-NMR (300 MHz, DMSO-*d*_6_): δ = 1.26 (3H, t, *J* = 7.2 Hz, -CH_3_), 1.33–1.38 (6H, m, -CH_3_), 3.03–3.10 (4H, m, -CH_2_-), 3.71 (3H, s, -OCH3), 4.14–4.16 (2H, m, -CH_2_-), 4.21 (2H, s, -CH_2_-), 6.89 (2H, d, *J* = 9.1 Hz, 1,4-disubstituted benzene), 7.49 (2H, d, *J* = 9.1 Hz, 1,4-disubstituted benzene), 7.98 (1H, dd, *J*_1_ = 1.9 Hz, *J*_2_ = 8.6 Hz, quinoxalin), 8.14 (1H, d, *J* = 8.6 Hz, quinoxalin), 8.21 (1H, d, *J* = 1.8 Hz, quinoxalin), 10.24 (1H, s, -NH). ^13^C-NMR (75 MHz, DMSO-*d*_6_): δ =11.96, 12.01, 15.50, 27.87, 37.94, 40.33, 55.63, 114.39, 121.09, 127.80, 128.27, 129.03, 129.65, 132.39, 140.31, 141.03, 150.94, 154.61, 155.83, 158.98, 159.14, 165.55. HRMS (*m*/*z*): [M + H]^+^ calcd for C_25_H_28_N_6_O2S: 477.2067; found: 477.2066.

##### 4.1.7.4 2-((5-(2,3-Diethylquinoxalin-6-yl)-4-ethyl-4*H*-1,2,4-triazol-3-yl)thio)-*N*-(4-fluorophenyl)acetamide (**7d**)

Yield: 74%, white. M.P.: 118.1–119.7 °C. IR (cm ^–1^ band): 3485 (N–H), 2985 (C–H), 1681 (C=O), 902. ^1^H-NMR (300 MHz, DMSO-*d*_6_): δ = 1.26 (3H, t, *J* = 7.2 Hz, -CH_3_), 1.33–1.38 (6H, m, -CH_3_), 3.03–3.11 (4H, m, -CH_2_-), 4.16 (2H, q, *J* = 7.1 Hz, -CH_2_-), 4.30 (2H, s, -CH_2_-), 7.15–7.18 (2H, m, 1,4-disubstituted benzene), 7.23–7.28 (1H, m, 1,4-disubstituted benzene), 7.89–7.93 (1H, m, 1,4-disubstituted benzene), 7.99 (1H, dd, *J*_1_ = 1.9 Hz, *J*_2_ = 8.6 Hz, quinoxalin), 8.15 (1H, d, *J* = 8.6 Hz, quinoxalin), 8.22 (1H, d, *J* = 1.9 Hz, quinoxalin), 10.21 (1H, s, -NH). ^13^C-NMR (75 MHz, DMSO-*d*_6_): δ =11.96, 12.01, 15.50, 27.85, 37.56, 40.22, 115.89, 124.12, 124.94, 125.97, 127.80, 128.28, 129.03, 129.67, 140.32, 141.04, 150.89, 154.63, 158.99, 159.16, 166.79. HRMS (*m*/*z*): [M + H]^+^ calcd for C_24_H_25_N_6_OFS: 465.1867; found: 465.1860.

##### 4.1.7.5 *N*-(4-Chlorophenyl)-2-((5-(2,3-diethylquinoxalin-6-yl)-4-ethyl-4*H*-1,2,4-triazol-3-yl)thio)acetamide (**7e**)

Yield: 81%, white. M.P.: 177.4–179.1 °C. IR (cm ^–1^ band): 3506 (N–H), 2978 (C–H), 1680 (C=O), 827. ^1^H-NMR (300 MHz, DMSO-*d*_6_): δ = 1.26 (3H, t, *J* = 7.2 Hz, -CH_3_), 1.33–1.38 (6H, m, -CH_3_), 3.03–3.10 (4H, m, -CH_2_-), 4.15 (2H, q, *J* = 7.3 Hz, -CH_2_-), 4.24 (2H, s, -CH_2_-), 7.38 (2H, d, *J* = 8.9 Hz, 1,4-disubstituted benzene), 7.62 (2H, d, *J* = 8.9 Hz, 1,4-disubstituted benzene), 7.98 (1H, dd, *J*_1_ = 1.9 Hz, *J*_2_ = 8.6 Hz, quinoxalin), 8.14 (1H, d, *J* = 8.6 Hz, quinoxalin), 8.21 (1H, d, *J* = 1.9 Hz, quinoxalin), 10.53 (1H, s, -NH). ^13^C-NMR (75 MHz, DMSO-*d*_6_): δ =11.97, 12.02, 15.48, 27.85, 37.89, 40.36, 121.09, 127.55, 127.77, 128.29, 129.03, 129.25, 129.66, 138.24, 140.31, 141.03, 150.86, 154.64, 158.98, 159.15, 166.31. HRMS (*m*/*z*): [M + H]^+^ calcd for C_24_H_25_N_6_OSCl: 481.1572; found: 481.1565.

##### 4.1.7.6 2-((5-(2,3-Diethylquinoxalin-6-yl)-4-ethyl-4H-1,2,4-triazol-3-yl)thio)-*N*-(4-(trifluoromethyl)phenyl)acetamide (**7f**)

Yield: 85%, white. M.P.: 175.5–176.7 °C. IR (cm ^–1^ band): 3500 (N–H), 2978 (C–H), 1681 (C=O), 823. ^1^H-NMR (300 MHz, DMSO-*d*_6_): δ = 1.27 (3H, t, *J* = 7.2 Hz, -CH_3_), 1.33–1.38 (6H, m, -CH_3_), 3.03–3.10 (4H, m, -CH_2_-), 4.15 (2H, q, *J* = 7.1 Hz, -CH_2_-), 4.29 (2H, s, -CH_2_-), 7.69 (2H, d, *J* = 8.8 Hz, 1,4-disubstituted benzene), 7.80 (2H, d, *J* = 8.4 Hz, 1,4-disubstituted benzene), 7.98 (1H, dd, *J*_1_ = 1.9 Hz, *J*_2_ = 8.6 Hz, quinoxalin), 8.13 (1H, d, *J* = 8.7 Hz, quinoxalin), 8.21 (1H, d, *J* = 1.6 Hz, quinoxalin), 10.76 (1H, s, -NH). ^13^C-NMR (75 MHz, DMSO-*d*_6_): δ =11.97, 12.02, 15.49, 20.89, 27.87, 37.99, 40.49, 119.54, 127.79, 128.26, 128.30, 129.03, 129.62, 129.68, 132.96, 136.77, 140.31, 141.03, 150.93, 154.61, 158.98, 159.15, 165.83. HRMS (*m*/*z*): [M + H]^+^ calcd for C_25_H_25_N_6_OF_3_S: 515.1835; found: 515.1815.

### 4.2 Activity Studies

#### 4.2.1 Strains and Chemicals

*Candida* strains used in this study are reference strains, respectively *C. albicans* ATCC 10231, *C. glabrata* MYA 2951, *C. tropicalis* ATCC 750, *C. krusei* ATCC 34135. All strains were stored as 15% (v/v) glycerol stocks at −80 °C. The frozen stocks were subcultured on Sabouraud dextrose agar (SDA, Oxoid, Basingstoke, UK) and incubated at 37 °C for 24 h. Fresh cultures were made in RPMI-1640 (HiMedia, India) and incubated at 37 C for 24 h. Drug solutions were prepared by dissolving fluconazole (Abcr, Germany) in sterile distilled water with a final concentration of 128–0.125 μg/mL and by dissolving the synthesized compounds in dimethyl sulfoxide (DMSO, Sigma, USA) with a final concentration of 1024–1 μg/mL.

#### 4.2.2 Determination of the Minimum Inhibitory Concentration

The antifungal activity of the synthesized compounds was examined using 96-well microtiter plates and the broth microdilution method from Clinical and Laboratory Standards Institute (CLSI) document M27-A3. Briefly, the inoculum size was adjusted to 0.5–2.5 × 10^3^*Candida* cells/mL using RPMI1640 media (pH: 7.0) supplemented with 2% glucose. The microplates were incubated at 37 °C for 24–48 h. The first well with total growth inhibition was referred to as the minimum inhibitory concentration (MIC).^8^

#### 4.2.3 Effects on Mature Biofilm Formation

Antibiofilm activity of the synthesized compounds were tested. To form mature biofilm formation, sterile 96-well flat-bottomed microplate was seeded with standard inoculum (0.5 McFarland) of *Candida* reference strains (100 μL each well), followed by incubation for 24 h at 37 °C. After the incubation, unattached cells were removed by washing the microplates three times in phosphate-buffered saline (PBS). Based on their capacity to produce biofilms, synthesized compounds were tested at sub-inhibitory concentrations of 1/2 × MIC, MIC, and 2 × MIC. The compounds were added to each well and incubated for 24 h at 37 °C. Incubated overnight, the microplates were washed three times with PBS and 100 μL of 0.1% crystal violet was added to each well. The microplates were washed three times with PBS after 5 min at room temperature. Then, 150 μL of 0.04 N HCl-isopropanol and 50 μL of 0.25% sodium dodecyl sulfate (SDS) were added to each well. A microplate reader was used to read the absorbance values at 590 nm. Untreated biofilm served as the growth control. The values of the experimental group and growth control were used to determine the percentage of inhibition. Biofilm inhibition rate = (OD control – OD sample/OD control) × 100.^30^

### 4.3 Molecular Docking

X-ray crystal structures of the human 14-α-demethylases (PDB ID: 1EA1)^26^ were retrieved from the Protein Data Bank server (www.pdb.org, accessed 01 May 2021). Docking procedures were performed using standard procedure and same program interfaces as previously reported by our team.^29,31−33^

### 4.4 Molecular Dynamics Simulation

Molecular dynamics (MD) simulations, which are considered an important computational tool to evaluate the time-dependent stability of a ligand at an active site for a drug-receptor complex, were performed for compound **5d** within the scope of this study.^34^ Dynamic procedures were performed using same program interfaces as previously reported by our team.^35−43^

## Supporting Information Available

**Figure S1.** Compound **5a** IR spectrum**. Figure S2.** Compound **5a**^1^H-NMR spectrum. **Figure S3.** Compound **5a**^13^C-NMR spectrum. **Figure S4.** Compound **5a** HRMS report. **Figure S5.** Compound **5b** IR spectrum**. Figure S6.** Compound **5b**^1^H-NMR spectrum. **Figure S7.** Compound **5b**^13^C-NMR spectrum. **Figure S8.** Compound **5b** HRMS report. **Figure S9.** Compound **5c** IR spectrum**. Figure S10.** Compound **5c**^1^H-NMR spectrum. **Figure S11.** Compound **5c**^13^C-NMR spectrum. **Figure S12.** Compound **5c** HRMS report. **Figure S13.** Compound **5d** IR spectrum**. Figure S14.** Compound **5d**^1^H-NMR spectrum. **Figure S15.** Compound **5d**^13^C-NMR spectrum. **Figure S16.** Compound **5d** HRMS report. **Figure S17.** Compound **5e** IR spectrum**. Figure S18.** Compound **5e**^1^H-NMR spectrum. **Figure S19.** Compound **5e**^13^C-NMR spectrum. **Figure S20.** Compound **5e** HRMS report. **Figure S21.** Compound **5f** IR spectrum**. Figure S22.** Compound **5f**^1^H-NMR spectrum. **Figure S23.** Compound **5f**^13^C-NMR spectrum. **Figure S24.** Compound **5f** HRMS report. **Figure S25.** Compound **5 g** IR spectrum**. Figure S26.** Compound **5 g**^1^H-NMR spectrum. **Figure S27.** Compound **5 g**^13^C-NMR spectrum. **Figure S28.** Compound **5 g** HRMS report. **Figure S29.** Compound **5 h** IR spectrum**. Figure S30.** Compound **5 h**^1^H-NMR spectrum. **Figure S31.** Compound **5 h**^13^C-NMR spectrum. **Figure S32.** Compound **5 h** HRMS report. **Figure S33.** Compound **5i** IR spectrum**. Figure S34.** Compound **5i**^1^H-NMR spectrum. **Figure S35.** Compound **5i**^13^C-NMR spectrum. **Figure S36.** Compound **5i** HRMS report. **Figure S37.** Compound **5j** IR spectrum**. Figure S38.** Compound **5j**^1^H-NMR spectrum. **Figure S39.** Compound **5j**^13^C-NMR spectrum. **Figure S40.** Compound **5j** HRMS report. **Figure S41.** Compound **5 k** IR spectrum**. Figure S42.** Compound **5 k**^1^H-NMR spectrum. **Figure S43.** Compound **5 k**^13^C-NMR spectrum. **Figure S44.** Compound **5 k** HRMS report. **Figure S45.** Compound **7a** IR spectrum**. Figure S46.** Compound **7a**^1^H-NMR spectrum. **Figure S47.** Compound **7a**^13^C-NMR spectrum. **Figure S48.** Compound **7a** HRMS report. **Figure S49.** Compound **7b** IR spectrum**. Figure S50.** Compound **7b**^1^H-NMR spectrum. **Figure S51.** Compound **7b**^13^C-NMR spectrum. **Figure S52.** Compound **7b** HRMS report. **Figure S53.** Compound **7c** IR spectrum**. Figure S54.** Compound **7c**^1^H-NMR spectrum. **Figure S55.** Compound **7c**^13^C-NMR spectrum. **Figure S56.** Compound **7c** HRMS report. **Figure S57.** Compound **7d** IR spectrum**. Figure S58.** Compound **7d**^1^H-NMR spectrum. **Figure S59.** Compound **7d**^13^C-NMR spectrum. **Figure S60.** Compound **7d** HRMS report. **Figure S61.** Compound **7e** IR spectrum**. Figure S62.** Compound **7e**^1^H-NMR spectrum. **Figure S63.** Compound **7e**^13^C-NMR spectrum. **Figure S64.** Compound **7e** HRMS report. **Figure S65.** Compound **7f** IR spectrum**. Figure S66.** Compound **7f**^1^H-NMR spectrum. **Figure S67.** Compound **7f**^13^C-NMR spectrum. **Figure S68.** Compound **7f** HRMS report.

## References

[ref1] Ni T.; Chi X.; Xie F.; Li L.; Wu H.; Hao Y.; Wang X.; Zhang D.; Jiang Y. Design, synthesis, and evaluation of novel tetrazoles featuring isoxazole moiety as highly selective antifungal agents. Eur. J. Med. Chem. 2023, 246, 115007 10.1016/j.ejmech.2022.115007. 36502579

[ref2] Xie F.; Hao Y.; Bao J.; Liu J.; Liu Y.; Wang R.; Chi X.; Chai X.; Wang T.; Yu S.; Jin Y.; Yan L.; Zhang D.; Ni T. Design, synthesis, and in vitro evaluation of novel antifungal triazoles containing substituted 1, 2, 3-triazole-methoxyl side chains. Bioorg. Chem. 2022, 129, 106216 10.1016/j.bioorg.2022.106216. 36283177

[ref3] Chahal M.; Kaushik C. P.; Luxmi R.; Kumar D.; Kumar A. Synthesis, antimicrobial, and antioxidant activities of disubstituted 1, 2, 3-triazoles with amide-hydroxyl functionality. Med. Chem. Res. 2023, 32, 85–98. 10.1007/s00044-022-02993-w.

[ref4] Faazil S.; Malik M. S.; Ahmed S. A.; Alsantali R. I.; Yedla P.; Alsharif M. A.; Shaikh I. N.; Kamal A. Novel linezolid-based oxazolidinones as potent anticandidiasis and antitubercular agents. Bioorg. Chem. 2022, 126, 105869 10.1016/j.bioorg.2022.105869. 35598571

[ref5] Guo M.-b.; Yan Z.-z.; Wang X.; Xu H.; Guo C.; Hou Z.; Gong P. Design, synthesis and antifungal activities of novel triazole derivatives with selenium-containing hydrophobic side chains. Bioorg. Med. Chem. Lett. 2022, 78, 129044 10.1016/j.bmcl.2022.129044. 36336315

[ref6] Ni T.; Ding Z.; Xie F.; Hao Y.; Bao J.; Zhang J.; Yu S.; Jiang Y.; Zhang D. Design, synthesis, and in vitro and in vivo antifungal activity of novel triazoles containing phenylethynyl pyrazole side chains. Molecules 2022, 27, 3370 10.3390/molecules27113370. 35684308 PMC9182106

[ref7] Yin W.; Liu L.; Jiang H.; Wu T.; Cui H.; Zhang Y.; Gao Z.; Sun Y.; Qin Q.; Zhao L.; Su X.; Zhao D.; Cheng M. Design, synthesis, and evaluation of novel 3-thiophene derivatives as potent fungistatic and fungicidal reagents based on a conformational restriction strategy. Eur. J. Med. Chem. 2022, 233, 114195 10.1016/j.ejmech.2022.114195. 35255313

[ref8] Aydin M.; Ozturk A.; Duran T.; Ozmen U. O.; Sumlu E.; Ayan E. B.; Korucu E. N. In vitro antifungal and antibiofilm activities of novel sulfonyl hydrazone derivatives against Candida spp. J. Med. Mycol. 2023, 33, 101327 10.1016/j.mycmed.2022.101327. 36272382

[ref9] Sadanandan B.; Ashrit P.; Nataraj L. K.; Shetty K.; Jogalekar A. P.; Vaniyamparambath V.; Hemanth B. High throughput comparative assessment of biofilm formation of Candida glabrata on polystyrene material. Korean J. Chem. Eng. 2022, 39, 1277–1286. 10.1007/s11814-021-1054-3.

[ref10] Flemming H. C.; Van Hullebusch E. D.; Neu T. R.; Nielsen P. H.; Seviour T.; Stoodley P.; Wingender J.; Wuertz S. The biofilm matrix: Multitasking in a shared space. Nat. Rev. Microbiol. 2023, 70–86. 10.1038/s41579-022-00791-0. 36127518

[ref11] El-Hazek R. M.; Elkenawy N. M.; Zaher N. H.; El-Gazzar M. G. Green synthesis of novel antifungal 1, 2, 4-triazoles effective against γ-irradiated Candida parapsilosis. Arch. Pharm. 2022, 355, 2100287 10.1002/ardp.202100287. 34708424

[ref12] Huang M.; Duan W. G.; Lin G. S.; Li B. Y. Synthesis, antifungal activity, 3D-QSAR, and molecular docking study of novel menthol-derived 1, 2, 4-triazole-thioether compounds. Molecules 2021, 26, 6948 10.3390/molecules26226948. 34834038 PMC8618492

[ref13] Zhu T.; Chen X.; Li C.; Tu J.; Liu N.; Xu D.; Sheng C. Lanosterol 14α-demethylase (CYP51)/histone deacetylase (HDAC) dual inhibitors for treatment of Candida tropicalis and Cryptococcus neoformans infections. Eur. J. Med. Chem. 2021, 221, 113524 10.1016/j.ejmech.2021.113524. 33992927

[ref14] Amin N. H.; El-Saadi M. T.; Ibrahim A. A.; Abdel-Rahman H. M. Design, synthesis and mechanistic study of new 1, 2, 4-triazole derivatives as antimicrobial agents. Bioorg. Chem. 2021, 111, 104841 10.1016/j.bioorg.2021.104841. 33798851

[ref15] Bitla S.; Gayatri A. A.; Puchakayala M. R.; Bhukya V. K.; Vannada J.; Dhanavath R.; Kuthati B.; Kothula D.; Sagurthi S. R.; Atcha K. R. Design and synthesis, biological evaluation of bis-(1, 2, 3-and 1, 2, 4)-triazole derivatives as potential antimicrobial and antifungal agents. Bioorg. Med. Chem. Lett. 2021, 41, 128004 10.1016/j.bmcl.2021.128004. 33811989

[ref16] Nesaragi A. R.; Kamble R. R.; Bayannavar P. K.; Shaikh S. K. J.; Hoolageri S. R.; Kodasi B.; Joshi S. D.; Kumbar V. M. Microwave assisted regioselective synthesis of quinoline appended triazoles as potent anti-tubercular and antifungal agents via copper (I) catalyzed cycloaddition. Bioorg. Med. Chem. Lett. 2021, 41, 127984 10.1016/j.bmcl.2021.127984. 33766768

[ref17] Qi L.; Li M. C.; Bai J. C.; Ren Y. H.; Ma H. X. In vitro antifungal activities, molecular docking, and DFT studies of 4-amine-3-hydrazino-5-mercapto-1, 2, 4-triazole derivatives. Bioorg. Med. Chem. Lett. 2021, 40, 127902 10.1016/j.bmcl.2021.127902. 33684439

[ref18] Al-Wabli R. I.; Alsulami M. A.; Bukhari S. I.; Moubayed N. M.; Al-Mutairi M. S.; Attia M. I. Design, synthesis, and antimicrobial activity of certain new indole-1, 2, 4 triazole conjugates. Molecules 2021, 26, 2292 10.3390/molecules26082292. 33920952 PMC8071222

[ref19] Gondru R.; Kanugala S.; Raj S.; Kumar C. G.; Pasupuleti M.; Banothu J.; Bavantula R. 1, 2, 3-triazole-thiazole hybrids: Synthesis, in vitro antimicrobial activity and antibiofilm studies. Bioorg. Med. Chem. Lett. 2021, 33, 127746 10.1016/j.bmcl.2020.127746. 33333162

[ref20] Hassan M. Z.; Alsayari A.; Asiri Y. I.; Bin Muhsinah A. 1, 2, 4-Triazole-3-Thiones: Greener, One-Pot, Ionic Liquid Mediated Synthesis and Antifungal Activity. Polycyclic Aromat. Compd. 2023, 43, 167–175. 10.1080/10406638.2021.2009887.

[ref21] Sadeghian S.; Emami L.; Mojaddami A.; khabnadideh S.; Faghih Z.; Zomorodian K.; Rashidi M.; Rezaei Z. 1, 2, 4-Triazole derivatives as novel and potent antifungal agents: Design, synthesis and biological evaluation. J. Mol. Struct. 2023, 1271, 134039 10.1016/j.molstruc.2022.134039.

[ref22] Ahuja R.; Sidhu A.; Bala A.; Arora D.; Sharma P. Structure based approach for twin-enzyme targeted benzimidazolyl-1, 2, 4-triazole molecular hybrids as antifungal agents. Arabian J. Chem. 2020, 13, 5832–5848. 10.1016/j.arabjc.2020.04.020.

[ref23] Suárez-García J.; Cano-Herrera M. A.; María-Gaviria M.; Osorio-Echeverri V. M.; Mendieta-Zerón H.; Arias-Olivares D.; Benavides-Melo J.; García-Sánchez L. C.; García-Ortíz J.; Becerra-Buitrago A.; Valero-Rojas J.; Rodríguez-González M. A.; García-Eleno M. A.; Cuevas-Yañez E. Synthesis, characterization, in-vitro biological evaluation and theoretical studies of 1, 2, 3-triazoles derived from triclosan as difenoconazole analogues. J. Mol. Struct. 2023, 1280, 135053 10.1016/j.molstruc.2023.135053.

[ref24] Wani M. Y.; Alghamidi M. S. S.; Srivastava V.; Ahmad A.; Aqlan F. M.; Al-Bogami A. S. Click synthesis of pyrrolidine-based 1, 2, 3-triazole derivatives as antifungal agents causing cell cycle arrest and apoptosis in Candida auris. Bioorg. Chem. 2023, 136, 106562 10.1016/j.bioorg.2023.106562. 37119782

[ref25] Peixoto L. R.; Rosalen P. L.; Ferreira G. L. S.; Freires I. A.; de Carvalho F. G.; Castellano L. R.; de Castro R. D. Antifungal activity, mode of action and anti-biofilm effects of Laurus nobilis Linnaeus essential oil against Candida spp. Arch. Oral Biol. 2017, 73, 179–185. 10.1016/j.archoralbio.2016.10.013. 27771586

[ref26] Podust L. M.; Poulos T. L.; Waterman M. R. Crystal structure of cytochrome P450 14α-sterol demethylase (CYP51) from Mycobacterium tuberculosis in complex with azole inhibitors. Proc. Natl. Acad. Sci. 2001, 98, 3068–3073. 10.1073/pnas.061562898. 11248033 PMC30608

[ref27] Zrieq R.; Ahmad I.; Snoussi M.; Noumi E.; Iriti M.; Algahtani F. D.; Patel H.; Saeed M.; Tasleem M.; Sulaiman S.; Aouadi K.; Kadri A. Tomatidine and patchouli alcohol as inhibitors of SARS-CoV-2 enzymes (3CLpro, PLpro and NSP15) by molecular docking and molecular dynamics simulations. Int. J. Mol. Sci. 2021, 22, 10693 10.3390/ijms221910693. 34639036 PMC8509278

[ref28] Ahmad I.; Kumar D.; Patel H. Computational investigation of phytochemicals from Withania somnifera (Indian ginseng/ashwagandha) as plausible inhibitors of GluN2B-containing NMDA receptors. J. Biomol. Struct. Dyn. 2022, 40, 7991–8003. 10.1080/07391102.2021.1905553. 33970806

[ref29] Osmaniye D.; Karaca Ş.; Kurban B.; Baysal M.; Ahmad I.; Patel H.; Özkay Y.; Kaplancıklı Z. A. Design, synthesis, molecular docking and molecular dynamics studies of novel triazolothiadiazine derivatives containing furan or thiophene rings as anticancer agents. Bioorg. Chem. 2022, 122, 105709 10.1016/j.bioorg.2022.105709. 35255344

[ref30] Ozturk A.; Abdulmajed O.; Aydin M. Investigation of antifungal, antibiofilm and anti-filamentation activities of biocides against Candida isolates. Ann. Med. Res. 2020, 27, 2041–2046. 10.5455/annalsmedres.2020.05.478.

[ref31] Maestro, 10.6; Schrödinger, LLC: New York, NY, USA, 2020**.**

[ref32] Schrödinger LigPrep, version 3.8; Schrödinger, LLC: New York, NY, USA, 2020.

[ref33] Schrödinger Glide, version 7.1; Schrödinger, LLC: New York, NY, USA, 2020.

[ref34] Liu X.; Shi D.; Zhou S.; Liu H.; Liu H.; Yao X. Molecular dynamics simulations and novel drug discovery. Expert Opin. Drug Discovery 2018, 13, 23–37. 10.1080/17460441.2018.1403419. 29139324

[ref35] M.D.I. Tools Schrödinger, LLC: New York, NY, 2020, Schrödinger Release 2018–3: Prime, (2018).

[ref36] Sureshkumar B.; Mary Y. S.; Resmi K.; Suma S.; Armaković S.; Armaković S. J.; Van Alsenoy C.; Narayana B.; Sobhana D. Spectroscopic characterization of hydroxyquinoline derivatives with bromine and iodine atoms and theoretical investigation by DFT calculations, MD simulations and molecular docking studies. J. Mol. Struct. 2018, 1167, 95–106. 10.1016/j.molstruc.2018.04.077.

[ref37] Schrödinger Release 1: Desmond molecular dynamics system, version 3.7; DE Shaw Research, New York, NY, Maestro-Desmond Interoperability Tools, version, 3, 2014.

[ref38] Humphreys D. D.; Friesner R. A.; Berne B. J. A multiple-time-step molecular dynamics algorithm for macromolecules. J. Phys. Chem. 1994, 98, 6885–6892. 10.1021/j100078a035.

[ref39] Hoover W. G. Canonical dynamics: Equilibrium phase-space distributions. Phys. Rev. A 1985, 31, 1695 10.1103/PhysRevA.31.1695. 9895674

[ref40] Martyna G. J.; Tobias D. J.; Klein M. L. Constant pressure molecular dynamics algorithms. J. Chem. Phys. 1994, 101, 4177–4189. 10.1063/1.467468.

[ref41] Essmann U.; Perera L.; Berkowitz M. L.; Darden T.; Lee H.; Pedersen L. G. A smooth particle mesh Ewald method. J. Chem. Phys. 1995, 103, 8577–8593. 10.1063/1.470117.

[ref42] Silveira F. F.; de Souza J. O.; Hoelz L. V.; Campos V. R.; Jabor V. A.; Aguiar A. C.; Nonato M. C.; Albuquerque M. G.; Guido R. V.; Boechat N.; Pinheiro L. C. S. Comparative study between the anti-P. falciparum activity of triazolopyrimidine, pyrazolopyrimidine and quinoline derivatives and the identification of new PfDHODH inhibitors. Eur. J. Med. Chem. 2021, 209, 112941 10.1016/j.ejmech.2020.112941. 33158577

[ref43] Osmaniye D.; Evren A. E.; Karaca Ş.; Özkay Y.; Kaplancıklı Z. A. Novel thiadiazol derivatives; design, synthesis, biological activity, molecular docking and molecular dynamics. J. Mol. Struct. 2023, 1272, 134171 10.1016/j.molstruc.2022.134171.

