# Supplementary material for: Synthesis, Antifungal Activities, Molecular Docking and Molecular Dynamic Studies of Novel Quinoxaline-Triazole Compounds
Source: ACS Omega. 2023 Jun 26;8(27):24573–85. doi: 10.1021/acsomega.3c02797 (PMC10339406; doi:10.1021/acsomega.3c02797)
Supplement: Supplementary file 1 — ao3c02797_si_001.pdf [file ao3c02797_si_001.pdf]

Synthesis, Antifungal Activities, Molecular Docking and Molecular Dynamic Studies of Novel Quinoxaline-Triazole Compounds

Derya Osmaniye[a,b*], Nurnehir Baltacı Bozkurt[c], Serkan Levent[a,b], Gamze Benli Yardımcı[c], Begüm Nurpelin Sağlık[a,b], Yusuf Ozkay[a,b], Zafer Asım Kaplancıklı[a]

[a]D*epartment of Pharmaceutical Chemistry, Faculty of Pharmacy, Anadolu University, 26470 Eskişehir, Turkey*

[b]Central Research Laboratory (MERLAB)*, Faculty of Pharmacy, Anadolu University, 26470 Eskişehir, Turkey*

[c]D*epartment of Pharmaceutical Microbiology, Faculty of Pharmacy, Afyonkarahisar Health Sciences University,03030 Afyonkarahisar, Turkey*

* Corresponding author.

E-mail address: dosmaniye@anadolu.edu.tr (D.Osmaniye).

Tel: +90-222-3350580/3779 *Fax*: +90-222-3350750.

Address: Anadolu University, Faculty of Pharmacy, Department of Pharmaceutical Chemistry, 26470, Eskişehir, Turkey.

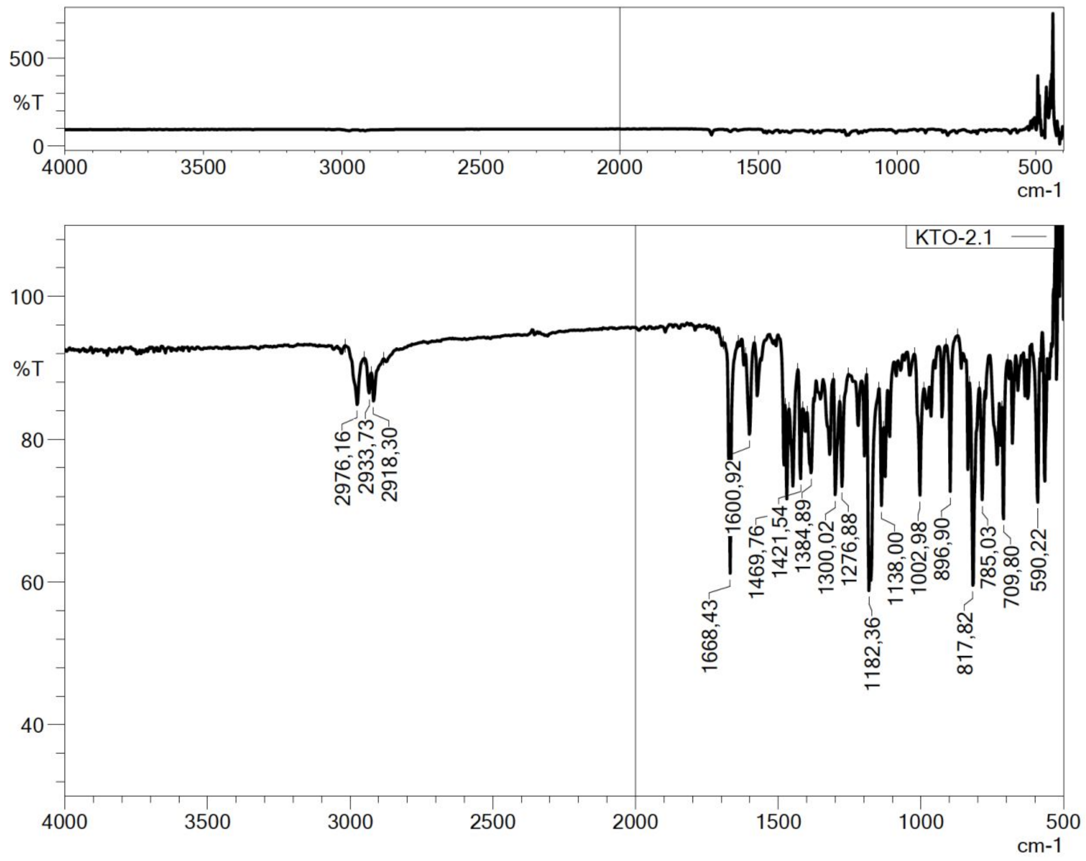

Figure S1. Compound **5a** IR spectrum

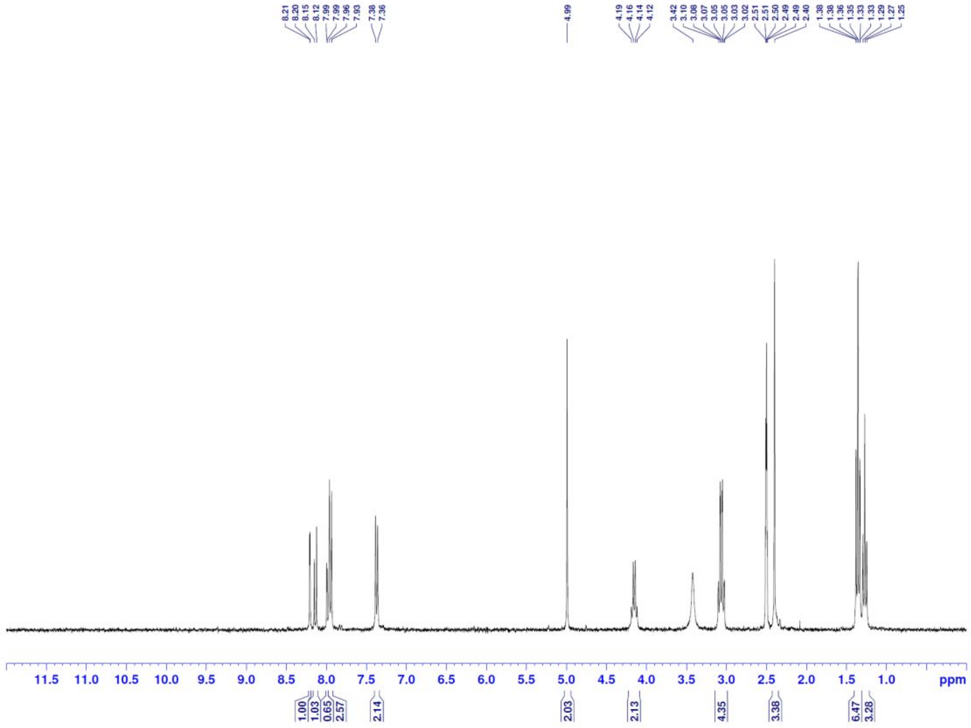

Figure S2. Compound **5a** ^{1}H-NMR spectrum

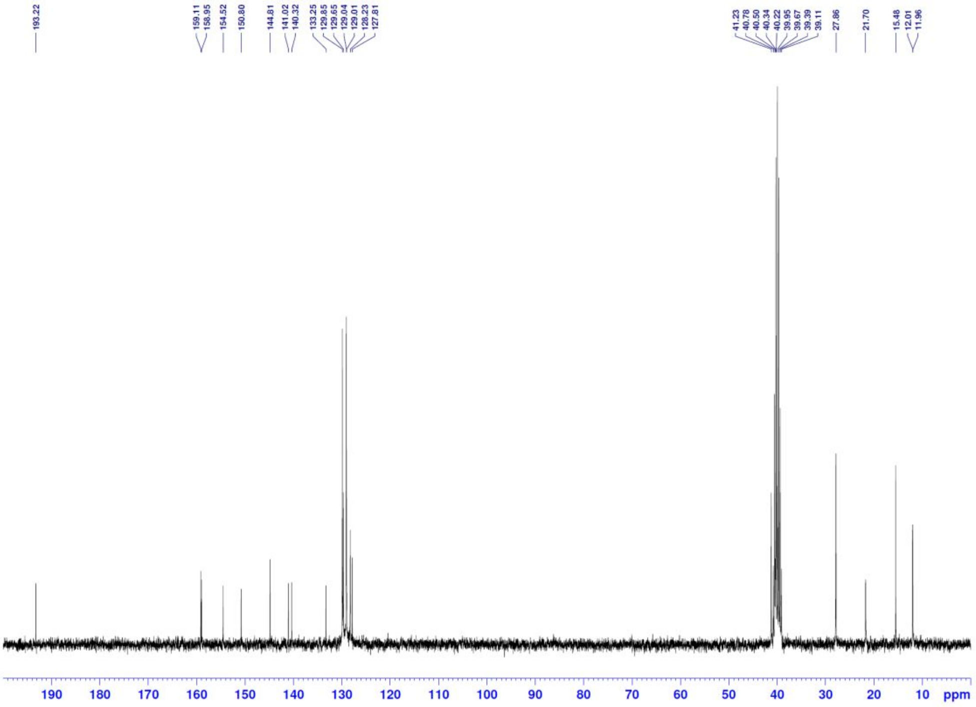

Figure S3. Compound **5a** ^{13}C-NMR spectrum

Formula Predictor Report - KTO-2_186.lcd

Page 1 of 1

Data File: C:\LabSolutions\Data\Analiz\derya\KTO-2_186.lcd

Elmt	Val.	Min	Max	Elmt	Val.	Min	Max	Elmt	Val.	Min	Max	Elmt	Val.	Min	Max	Use Adduct
H	1	8	40	O	2	0	6	S	2	1	1	Ru	2	0	0	H
C	4	9	40	F	1	0	0	Cl	1	0	0	Pd	2	0	0	
N	3	2	6	P	3	0	0	Br	1	0	0	I	3	0	0	

Error Margin (ppm): 5
HC Ratio: unlimited
Max Isotopes: 3
MSn Iso RI (%): 10.00

DBE Range: 5.0 - 25.0
Apply N Rule: yes
Isotope RI (%): 1.00
MSn Logic Mode: AND

Electron Ions: both
Use MSn Info: yes
Isotope Res: 9000
Max Results: 200

Event#: 1 MS(E+) Ret. Time : 4.747 -> 4.747 Scan# : 713 -> 713

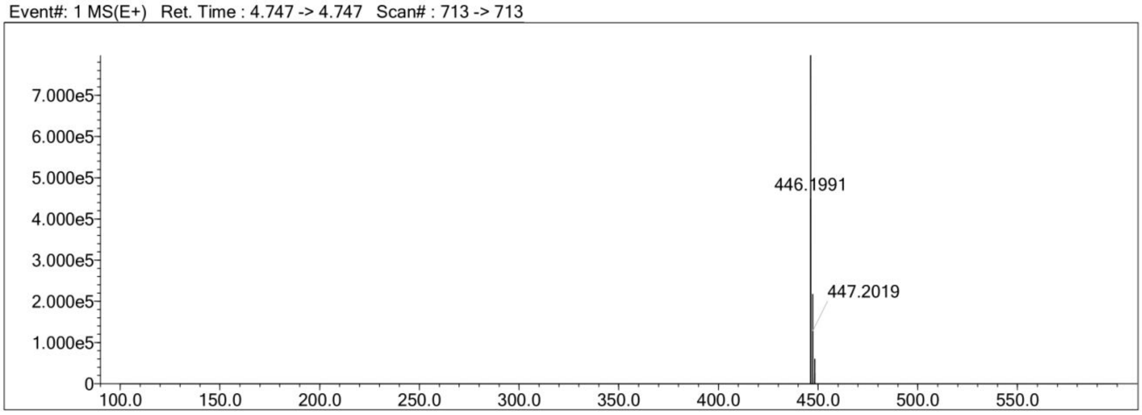

Measured region for 446.1991 m/z

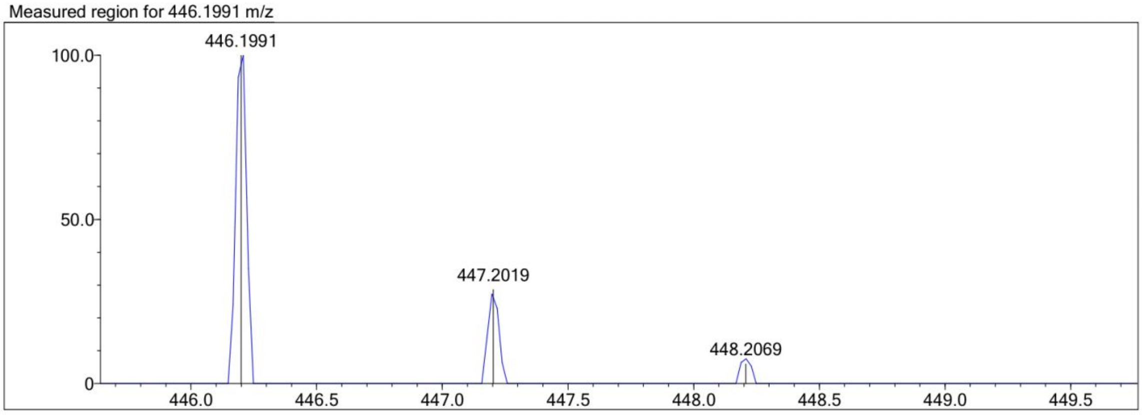

C25 H27 N5 O S [M+H]+ : Predicted region for 446.2009 m/z

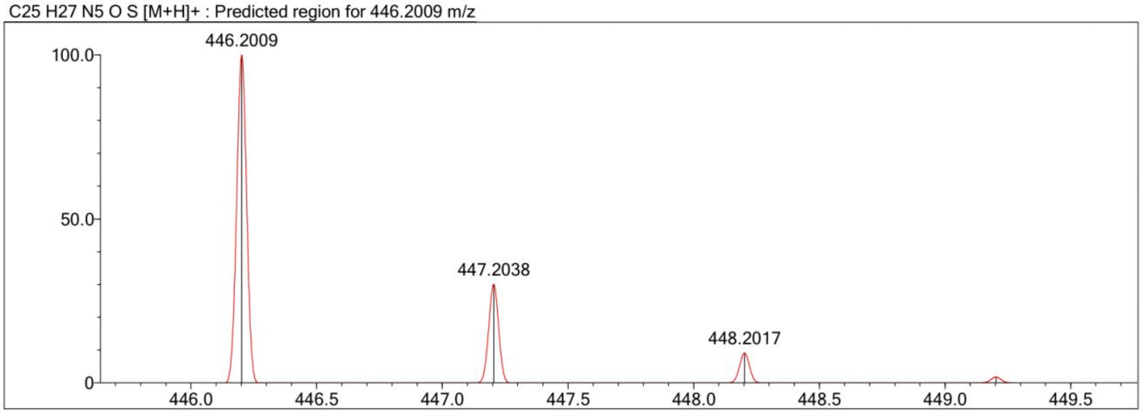

Rank	Score	Formula (M)	Ion	Meas. m/z	Pred. m/z	Df. (mDa)	Df. (ppm)	Iso	DBE
1	73.17	C25 H27 N5 O S	[M+H]+	446.1991	446.2009	-1.8	-4.03	79.17	15.0

Figure S4. Compound **5a** HRMS report

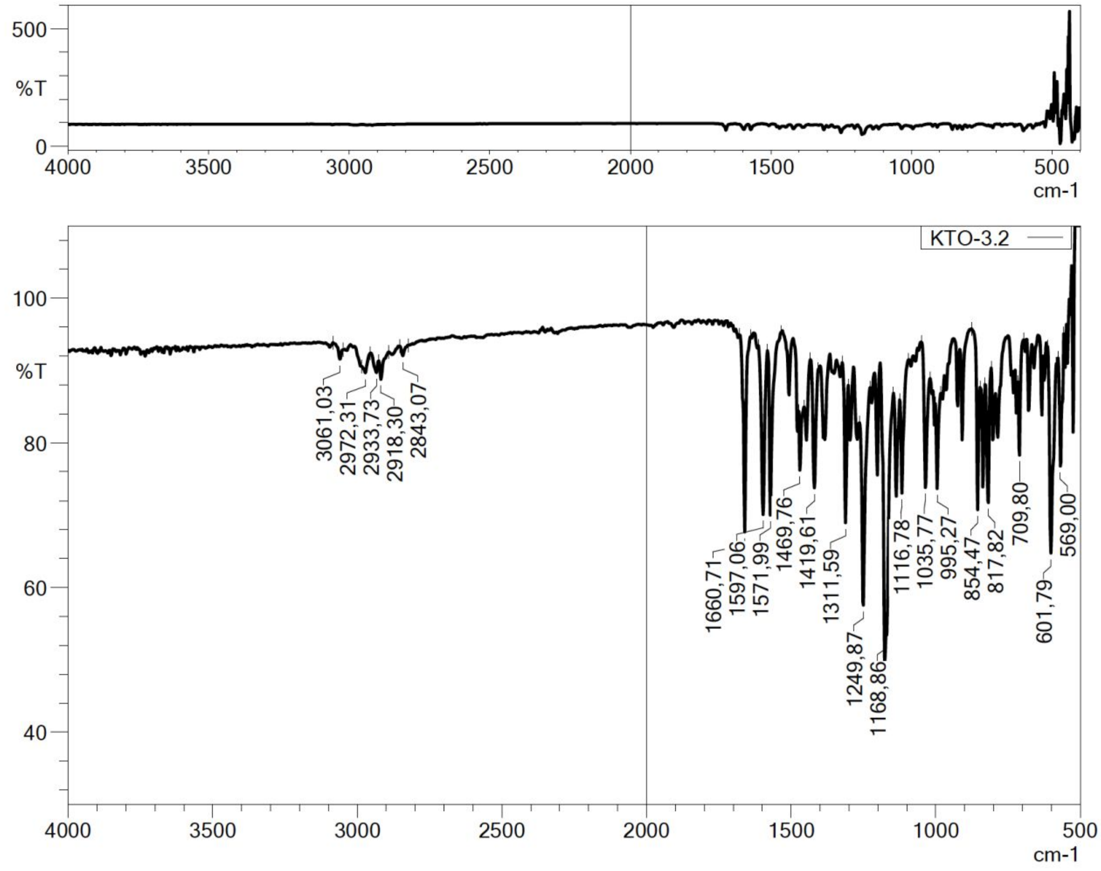

Figure S5. Compound **5b** IR spectrum

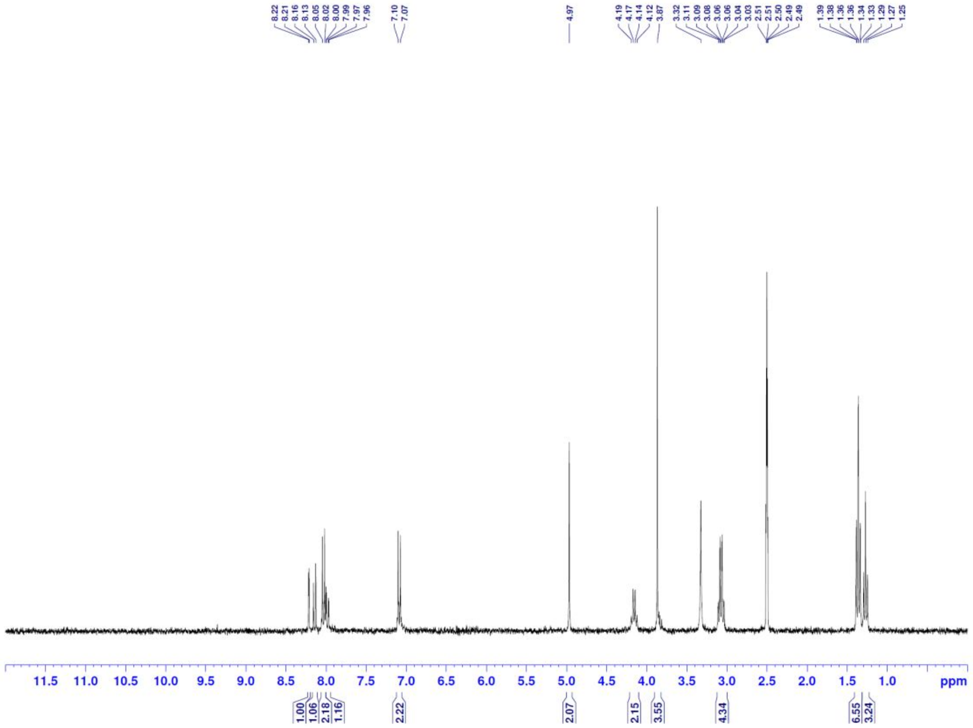

Figure S6. Compound **5b** ^{1}H-NMR spectrum

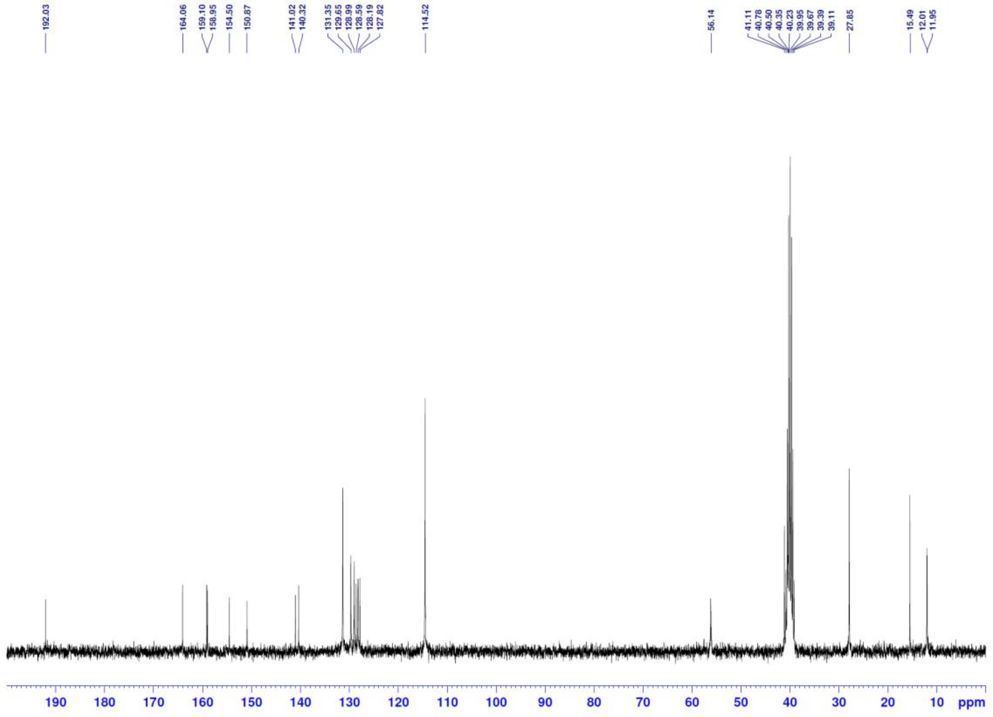

Figure S7. Compound **5b** ^{13}C-NMR spectrum

Formula Predictor Report - KTO-3_187.lcd

Page 1 of 1

Data File: C:\LabSolutions\Data\Analiz\derya\KTO-3_187.lcd

Elmt	Val.	Min	Max	Elmt	Val.	Min	Max	Elmt	Val.	Min	Max	Elmt	Val.	Min	Max	Use Adduct
H	1	8	40	O	2	0	6	S	2	1	1	Ru	2	0	0	H
C	4	9	40	F	1	0	0	Cl	1	0	0	Pd	2	0	0	
N	3	2	6	P	3	0	0	Br	1	0	0	I	3	0	0	

Error Margin (ppm):	5	DBE Range:	5.0 - 25.0	Electron Ions:	both
HC Ratio:	unlimited	Apply N Rule:	yes	Use MSn Info:	yes
Max Isotopes:	3	Isotope RI (%):	1.00	Isotope Res:	9000
MSn Iso RI (%):	10.00	MSn Logic Mode:	AND	Max Results:	200

Event#: 1 MS(E+) Ret. Time : 3.813 Scan# : 573

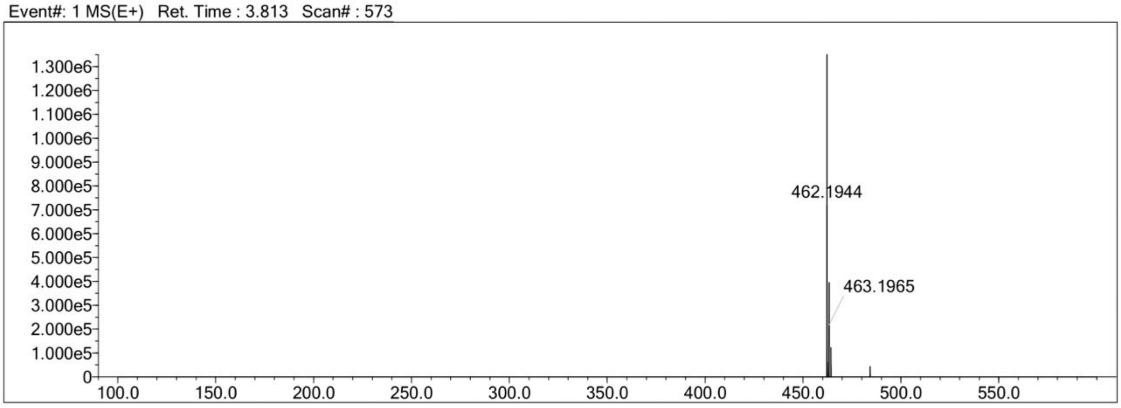

Measured region for 462.1944 m/z

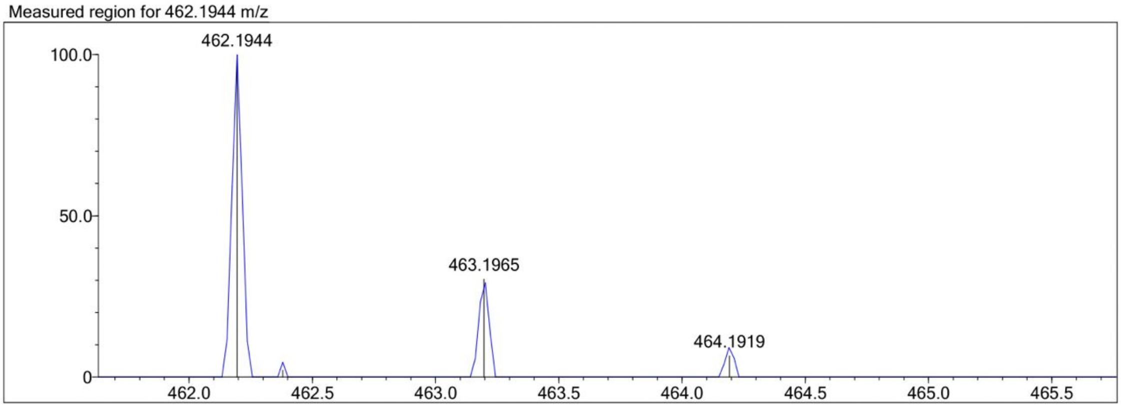

C25 H27 N5 O2 S [M+H]+ : Predicted region for 462.1958 m/z

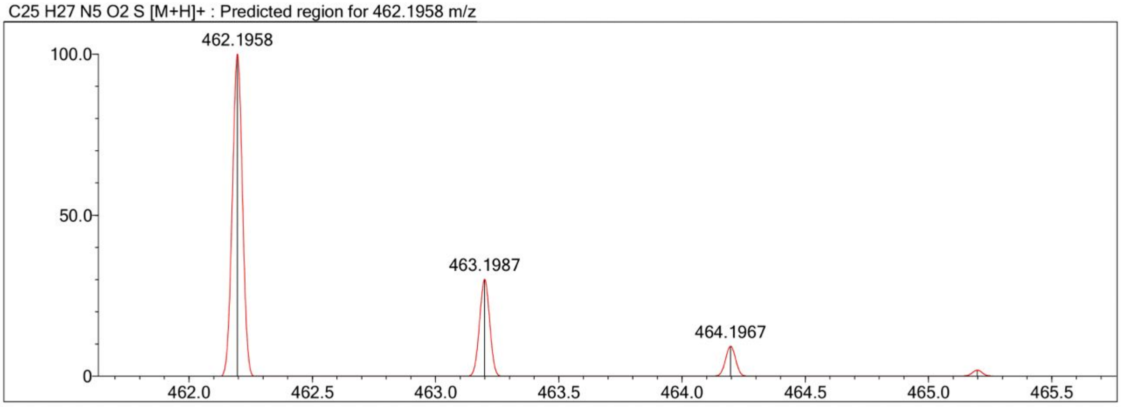

Rank	Score	Formula (M)	Ion	Meas. m/z	Pred. m/z	Df. (mDa)	Df. (ppm)	Iso	DBE
1	84.02	C25 H27 N5 O2 S	[M+H]+	462.1944	462.1958	-1.4	-3.03	88.51	15.0

Figure S8. Compound **5b** HRMS report

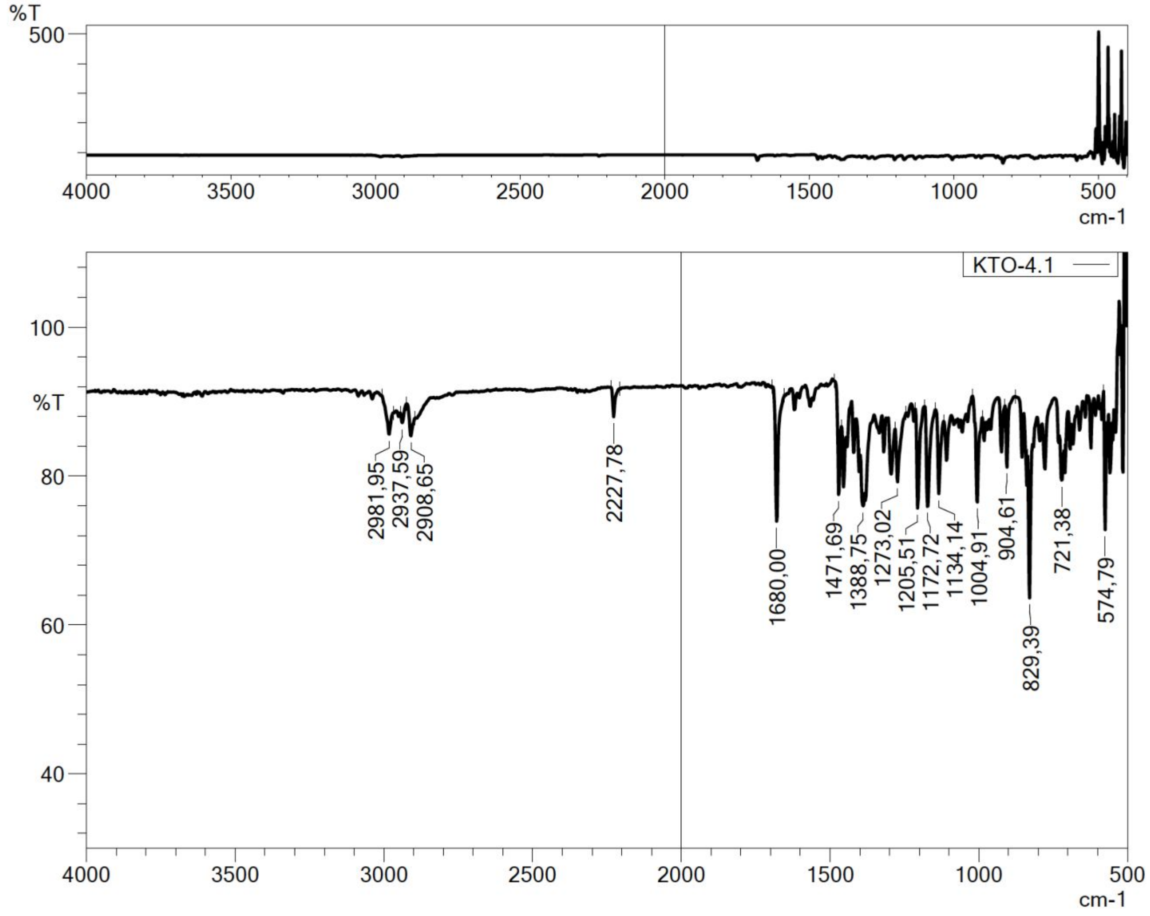

Figure S9. Compound **5c** HRMS report

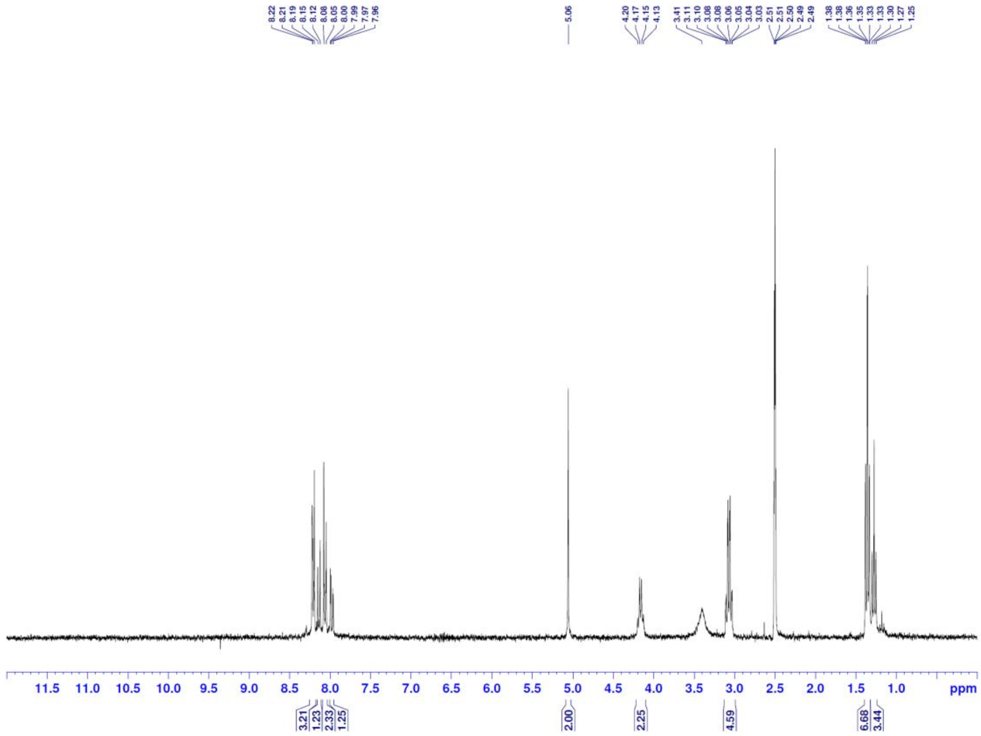

Figure S10. Compound **5c** ^{1}H-NMR spectrum

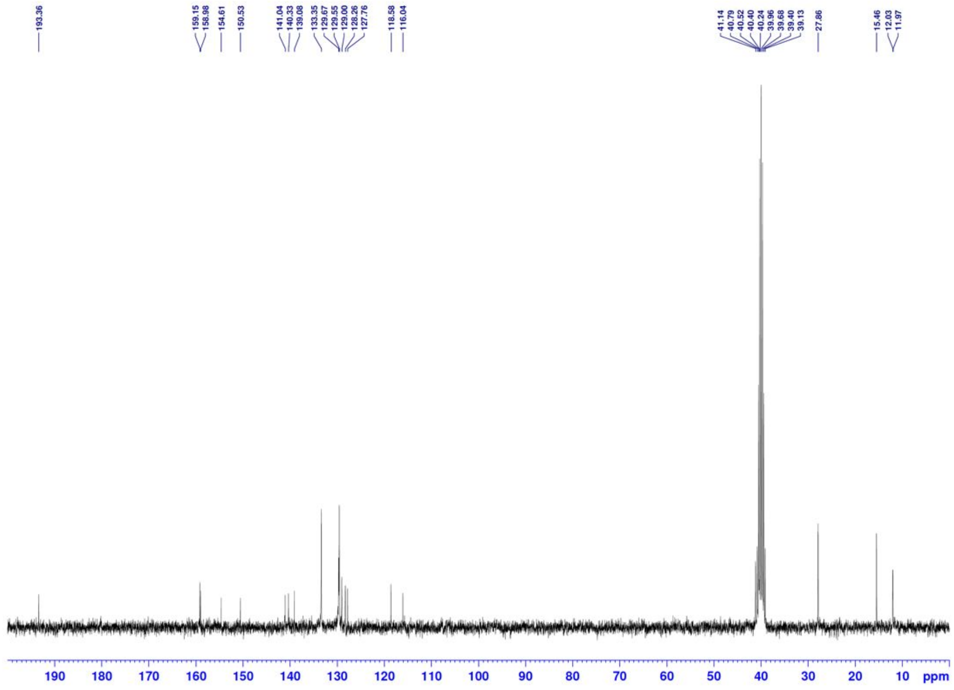

Figure S11. Compound **5c** ^{13}C-NMR spectrum

Data File: C:\LabSolutions\Data\Analiz\derya\KTO-4_188.lcd

Elmt	Val.	Min	Max	Elmt	Val.	Min	Max	Elmt	Val.	Min	Max	Elmt	Val.	Min	Max	Use Adduct
H	1	8	40	O	2	0	4	S	2	1	1	Ru	2	0	0	H
C	4	9	40	F	1	0	0	Cl	1	0	0	Pd	2	0	0	
N	3	2	6	P	3	0	0	Br	1	0	0	I	3	0	0	

Error Margin (ppm):	5	DBE Range:	5.0 - 25.0	Electron Ions:	both
HC Ratio:	unlimited	Apply N Rule:	yes	Use MSn Info:	yes
Max Isotopes:	3	Isotope RI (%):	1.00	Isotope Res:	9000
MSn Iso RI (%):	10.00	MSn Logic Mode:	AND	Max Results:	200

Event#: 1 MS(E+) Ret. Time : 3.547 Scan# : 533

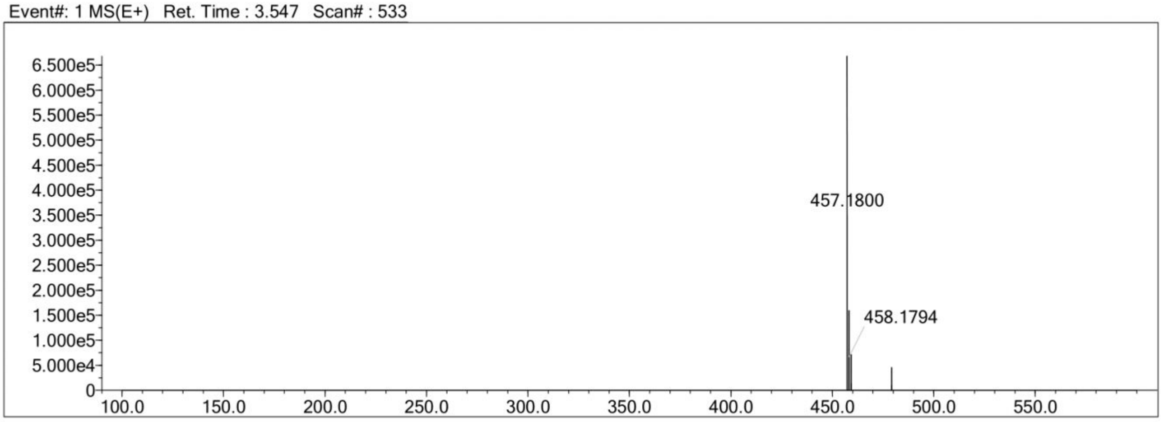

Measured region for 457.1800 m/z

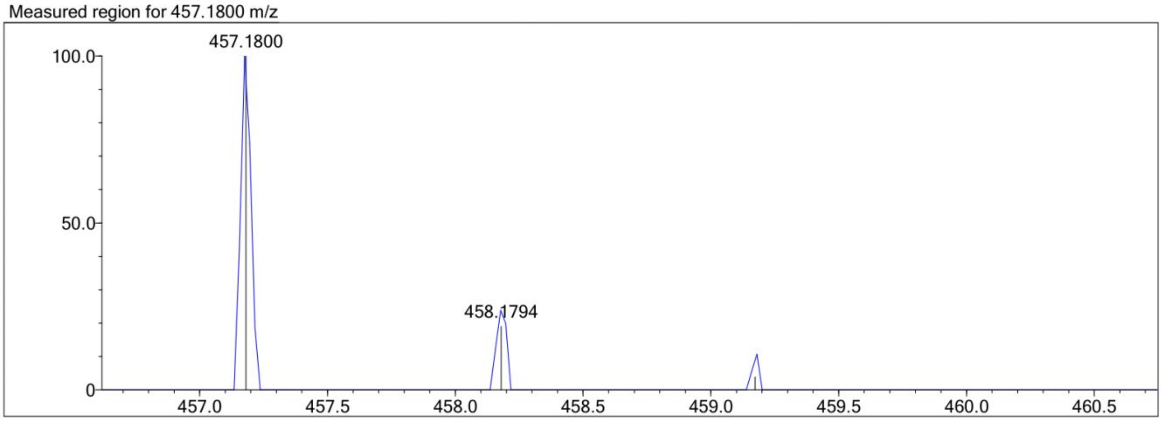

C25 H24 N6 O S [M+H]+ : Predicted region for 457.1805 m/z

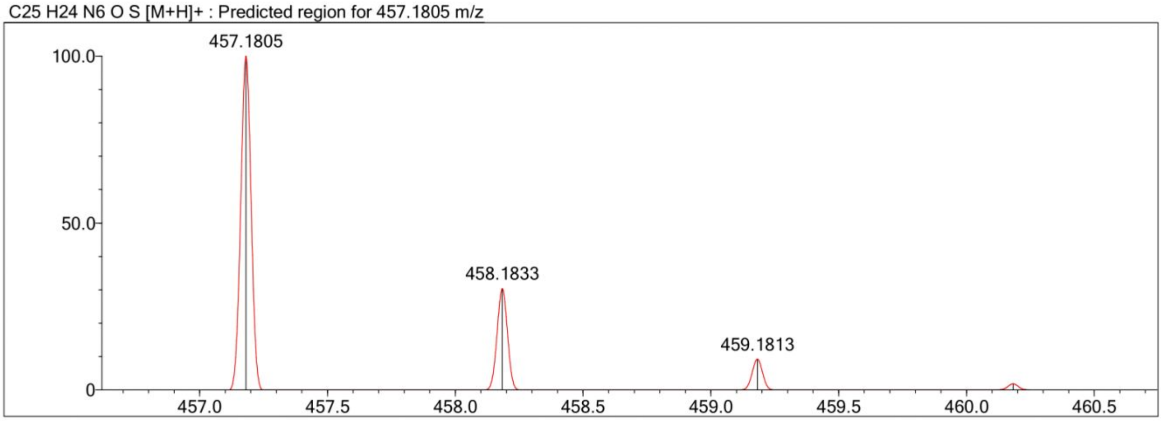

Rank	Score	Formula (M)	Ion	Meas. m/z	Pred. m/z	Df. (mDa)	Df. (ppm)	Iso	DBE
1	53.01	C25 H24 N6 O S	[M+H]+	457.1800	457.1805	-0.5	-1.09	53.13	17.0

Figure S12. Compound **5c** HRMS report

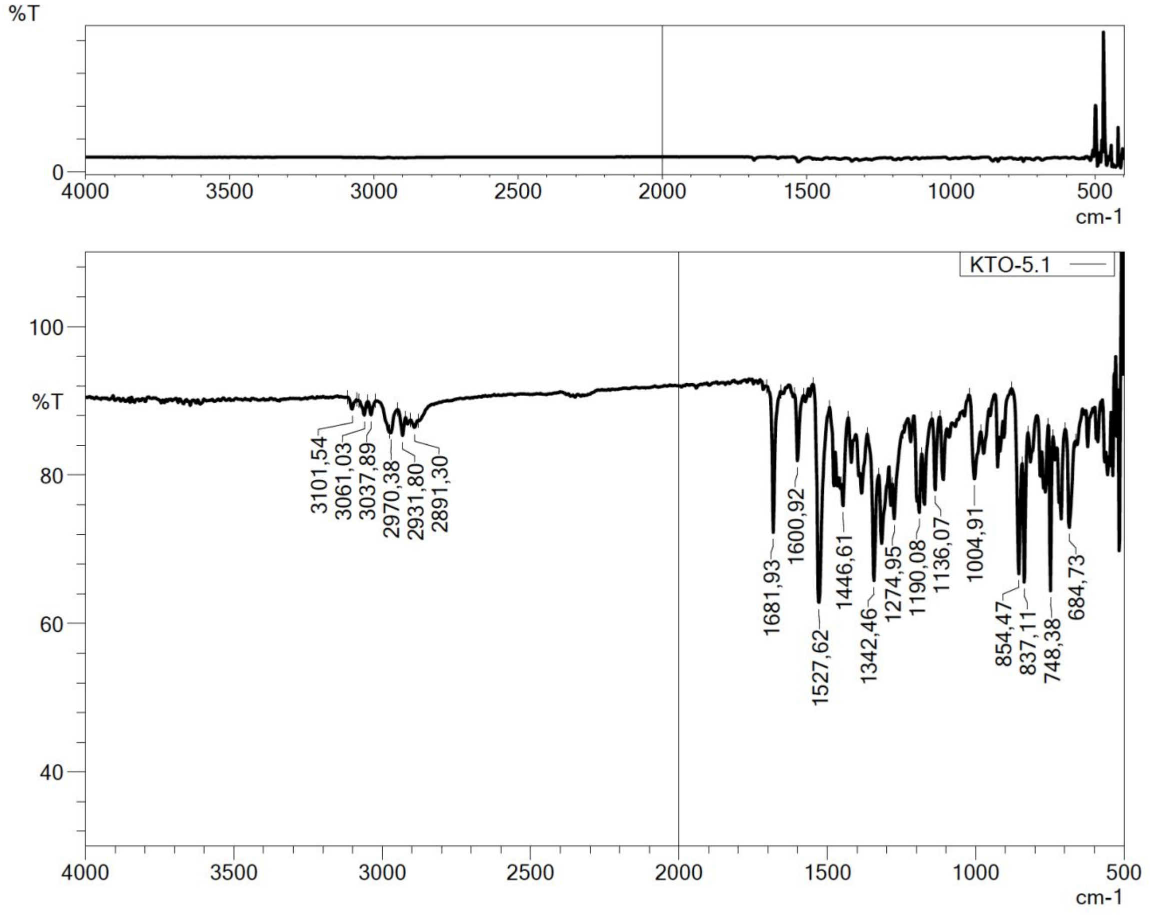

Figure S13. Compound **5d** IR report

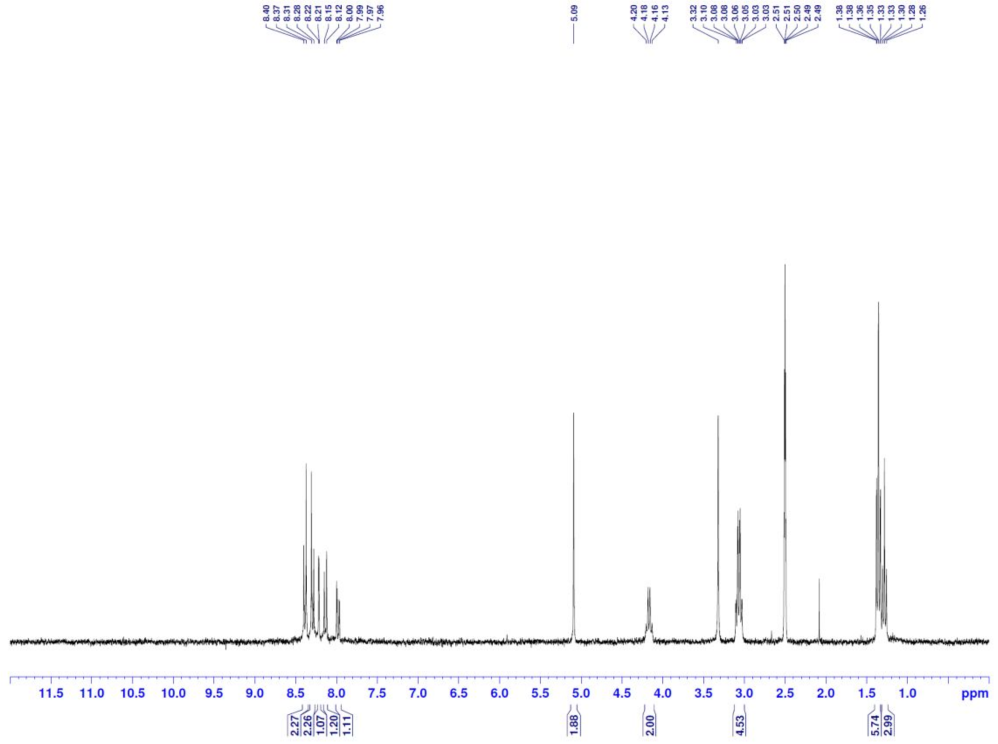

Figure S14. Compound **5d** ^{1}H-NMR spectrum

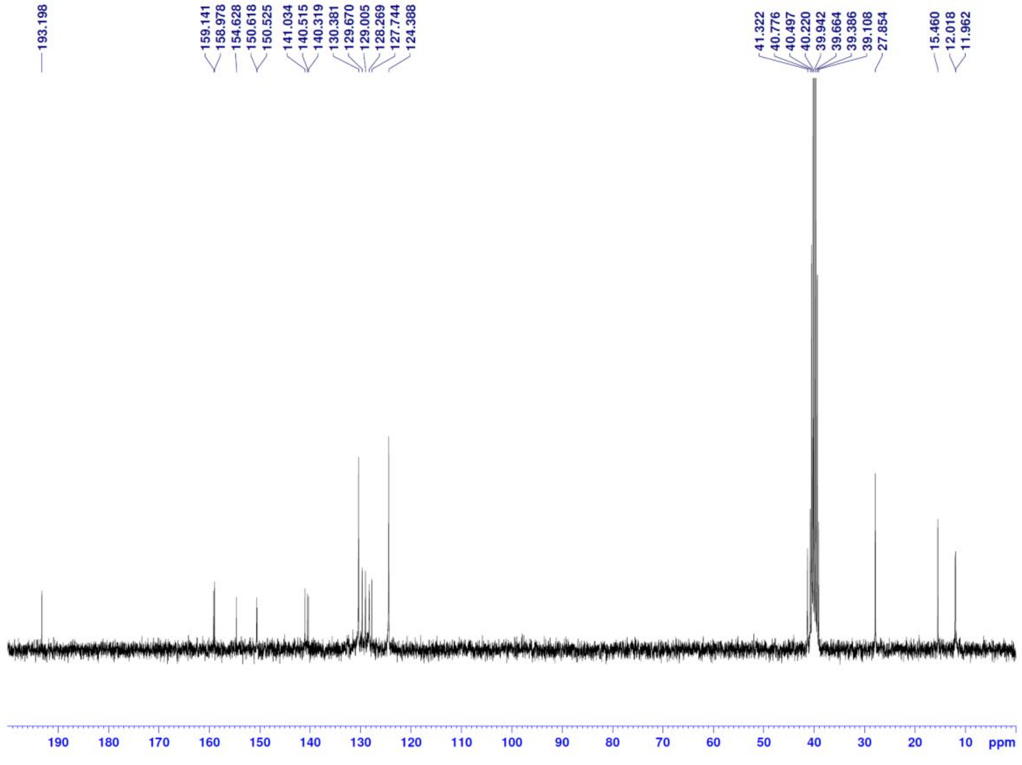

Figure S15. Compound **5d** ^{13}C-NMR spectrum

Formula Predictor Report - KTO-5_189.lcd Page 1 of 1

Data File: C:\LabSolutions\Data\Analiz\derya\KTO-5_189.lcd

Elmt	Val.	Min	Max	Elmt	Val.	Min	Max	Elmt	Val.	Min	Max	Elmt	Val.	Min	Max	Use Adduct
H	1	8	40	O	2	0	4	S	2	1	1	Ru	2	0	0	H
C	4	9	40	F	1	0	0	Cl	1	0	0	Pd	2	0	0	
N	3	2	6	P	3	0	0	Br	1	0	0	I	3	0	0	

Error Margin (ppm):	5	DBE Range:	5.0 - 25.0	Electron Ions:	both
HC Ratio:	unlimited	Apply N Rule:	yes	Use MSn Info:	yes
Max Isotopes:	3	Isotope RI (%):	1.00	Isotope Res:	9000
MSn Iso RI (%):	10.00	MSn Logic Mode:	AND	Max Results:	200

Event#: 1 MS(E+) Ret. Time : 4.000 Scan# : 601

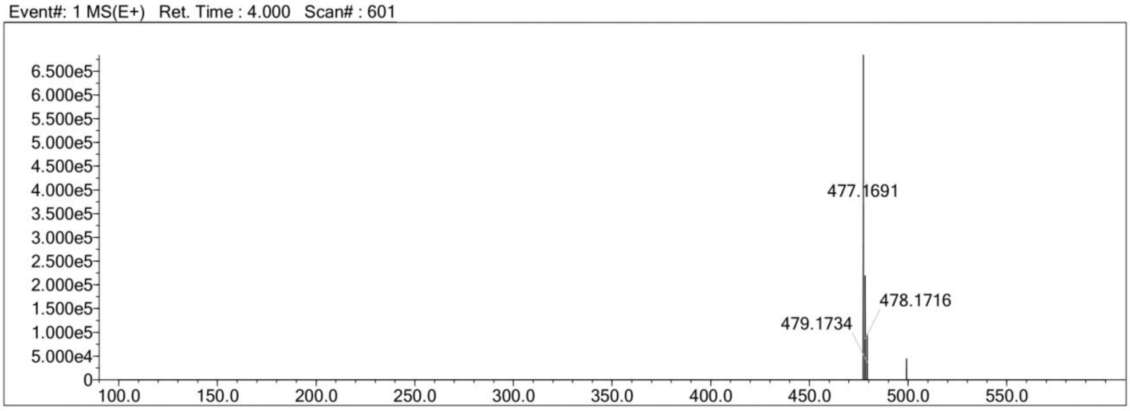

Measured region for 477.1691 m/z

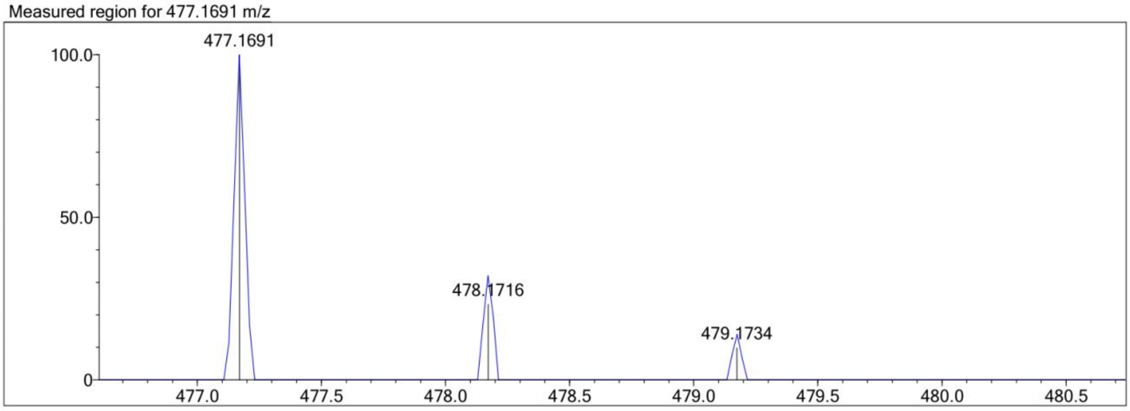

C24 H24 N6 O3 S [M+H]+ : Predicted region for 477.1703 m/z

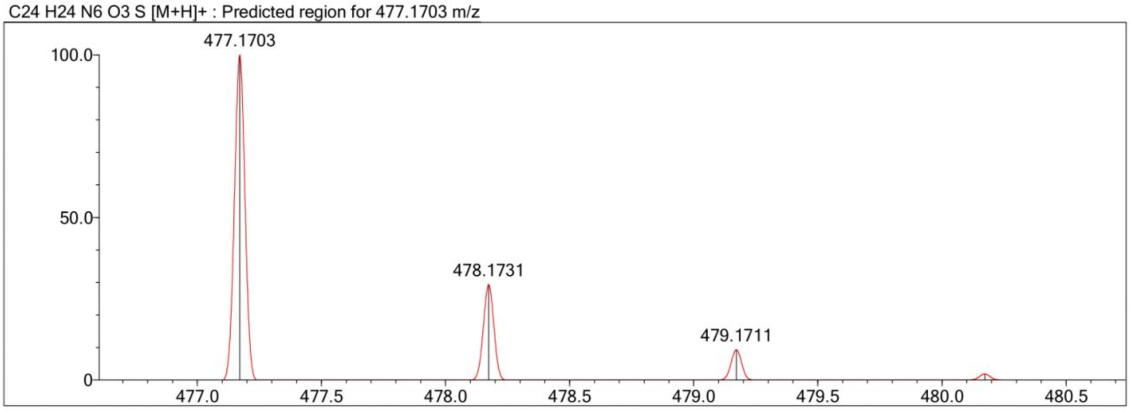

Rank	Score	Formula (M)	Ion	Meas. m/z	Pred. m/z	Df. (mDa)	Df. (ppm)	Iso	DBE
1	81.01	C24 H24 N6 O3 S	[M+H]+	477.1691	477.1703	-1.2	-2.51	84.19	16.0

Figure S16. Compound **5d** HRMS report

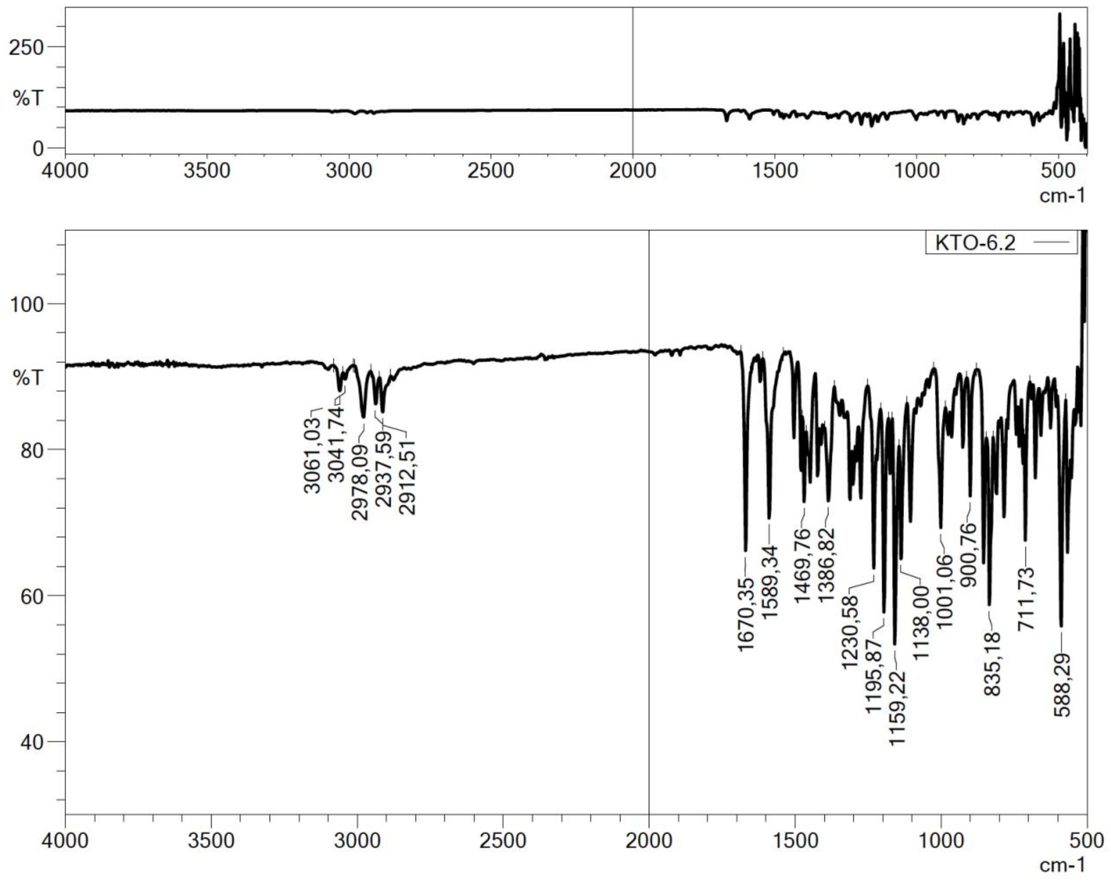

Figure S17. Compound **5e** IR report

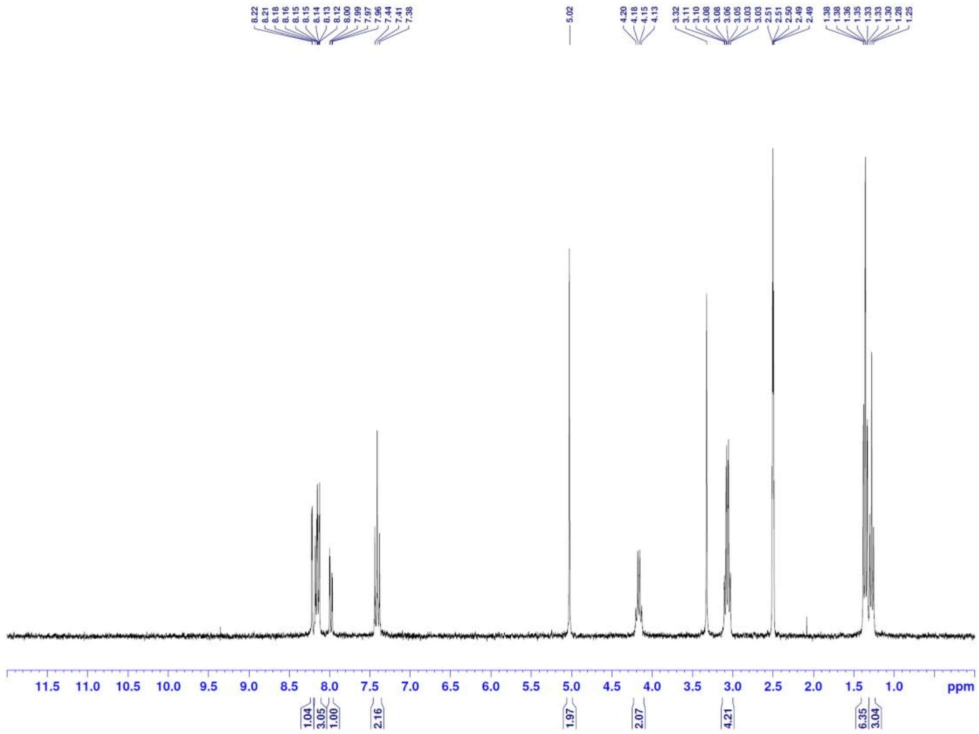

Figure S18. Compound **5e** ^{1}H-NMR spectrum

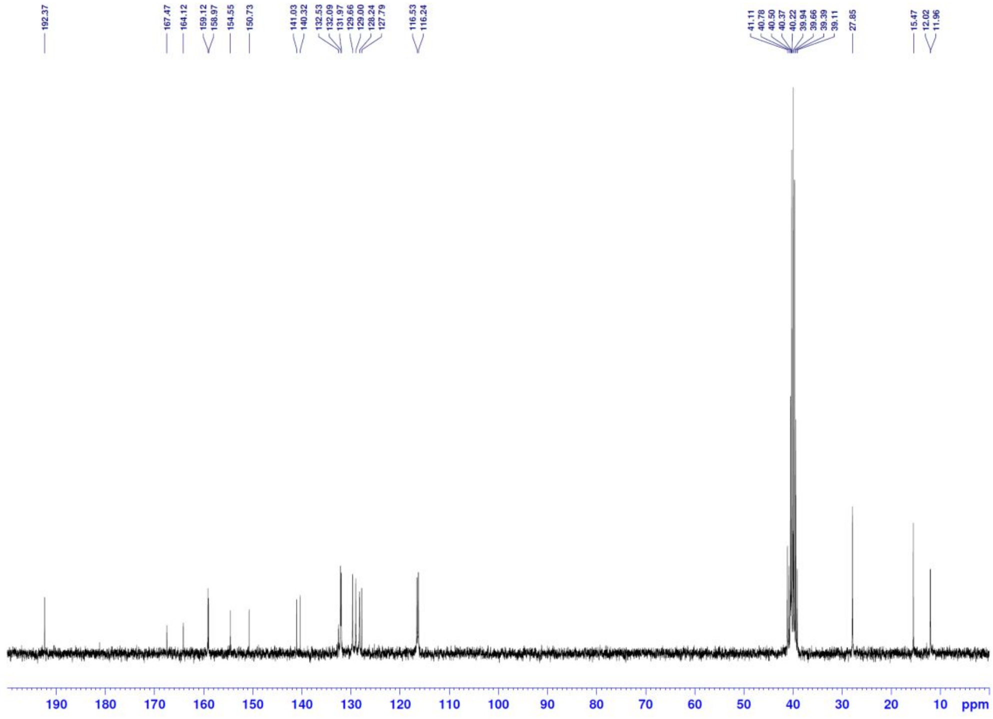

Figure S19. Compound **5e** ^{13}C-NMR spectrum

Formula Predictor Report - KTO-6_190.lcd Page 1 of 1

Data File: C:\LabSolutions\Data\Analiz\derya\KTO-6_190.lcd

Elmt	Val.	Min	Max	Elmt	Val.	Min	Max	Elmt	Val.	Min	Max	Elmt	Val.	Min	Max	Use Adduct
H	1	8	40	O	2	0	4	S	2	1	1	Ru	2	0	0	H
C	4	9	40	F	1	1	1	Cl	1	0	0	Pd	2	0	0	
N	3	2	6	P	3	0	0	Br	1	0	0	I	3	0	0	

Error Margin (ppm):	5	DBE Range:	5.0 - 25.0	Electron Ions:	both
HC Ratio:	unlimited	Apply N Rule:	yes	Use MSn Info:	yes
Max Isotopes:	3	Isotope RI (%):	1.00	Isotope Res:	9000
MSn Iso RI (%):	10.00	MSn Logic Mode:	AND	Max Results:	200

Event#: 1 MS(E+) Ret. Time : 3.840 Scan# : 577

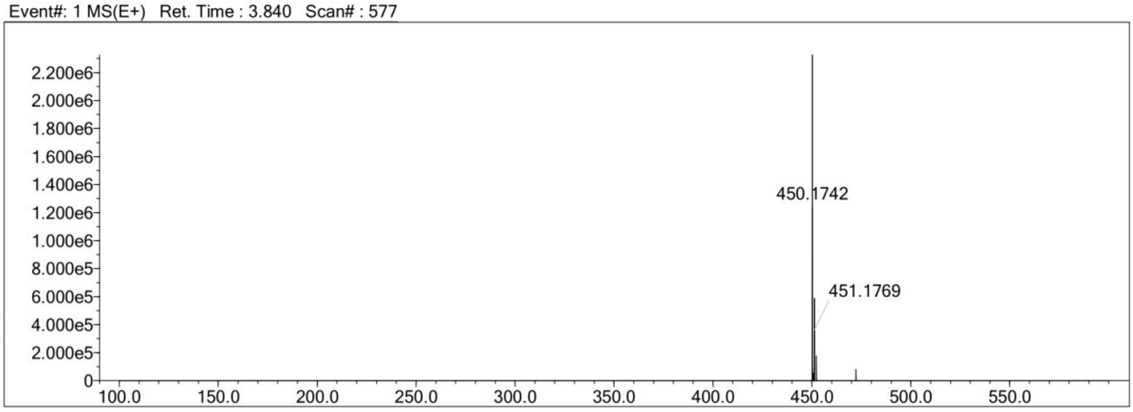

Measured region for 450.1742 m/z

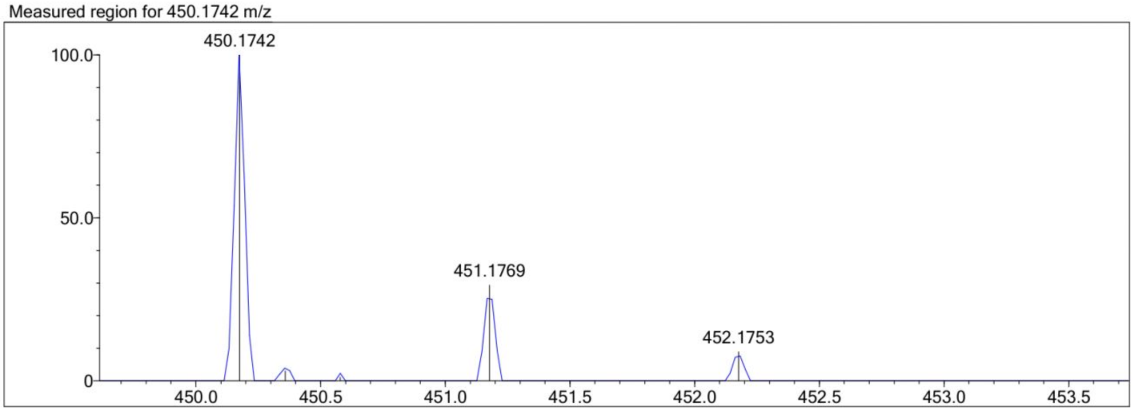

C24 H24 N5 O F S [M+H]+ : Predicted region for 450.1758 m/z

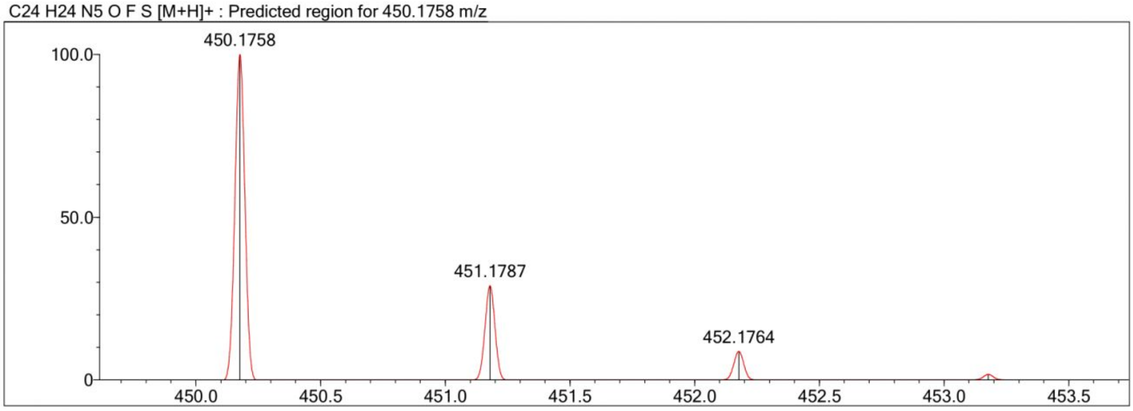

Rank	Score	Formula (M)	Ion	Meas. m/z	Pred. m/z	Df. (mDa)	Df. (ppm)	Iso	DBE
1	74.46	C24 H24 N5 O F S	[M+H]+	450.1742	450.1758	-1.6	-3.55	79.53	15.0

Figure S20. Compound **5e** HRMS report

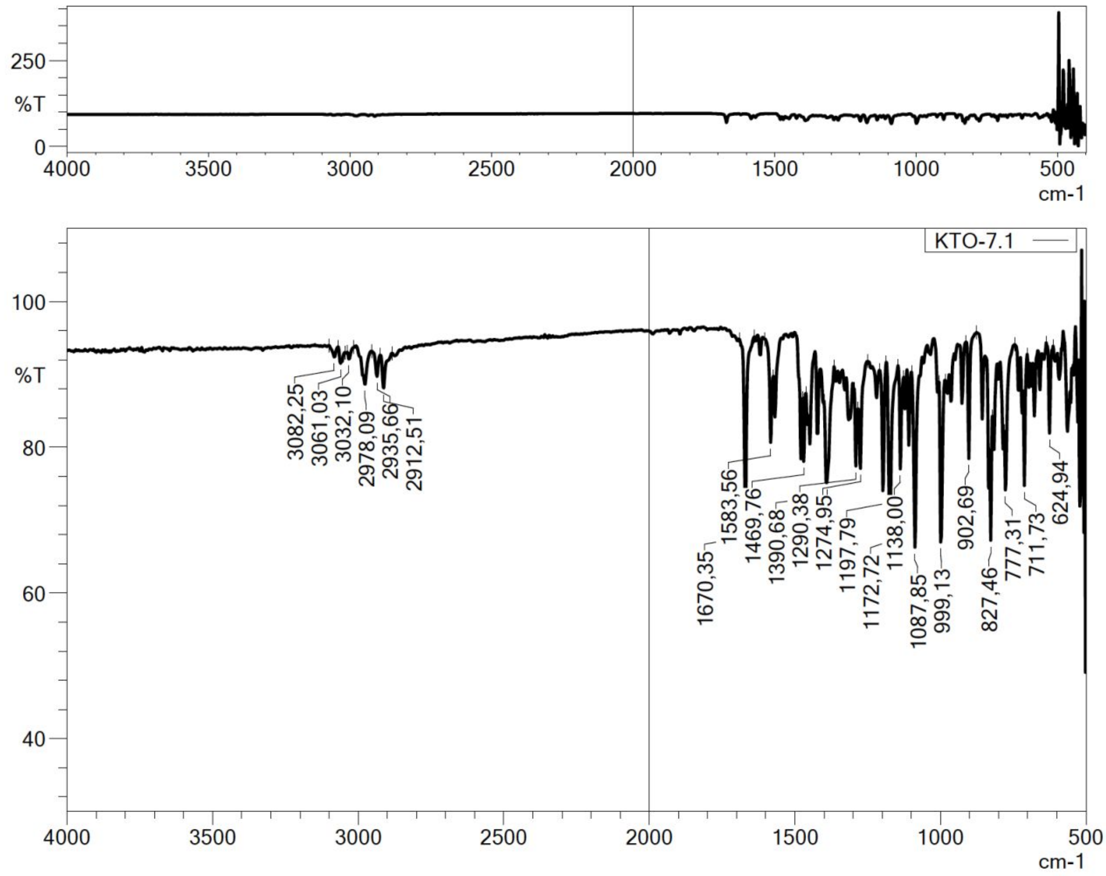

Figure S21. Compound **5f** IR report

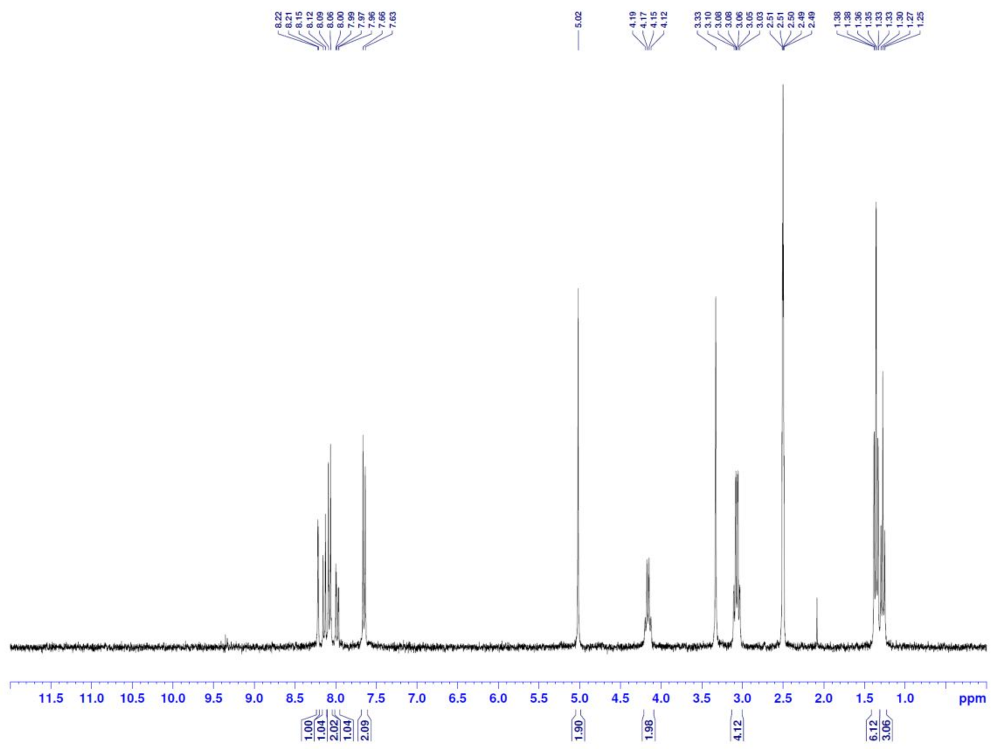

Figure S22. Compound **5f** ^{1}H-NMR spectrum

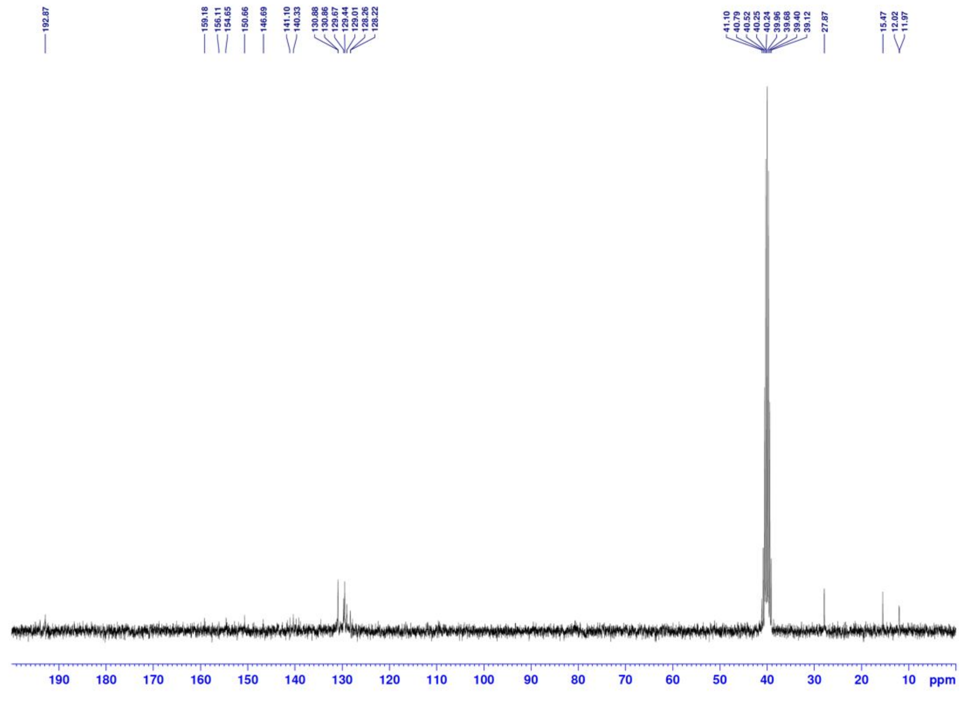

Figure S23. Compound **5f** ^{13}C-NMR spectrum

Data File: C:\LabSolutions\Data\Analiz\derya\KTO-7_191.lcd

Elmt	Val.	Min	Max	Elmt	Val.	Min	Max	Elmt	Val.	Min	Max	Elmt	Val.	Min	Max	Use Adduct
H	1	8	40	O	2	0	4	S	2	1	1	Ru	2	0	0	H
C	4	9	40	F	1	0	0	Cl	1	1	1	Pd	2	0	0	
N	3	2	6	P	3	0	0	Br	1	0	0	I	3	0	0	

Error Margin (ppm): 5
HC Ratio: unlimited
Max Isotopes: 3
MSn Iso RI (%): 10.00
DBE Range: 5.0 - 25.0
Apply N Rule: yes
Isotope RI (%): 1.00
MSn Logic Mode: AND
Electron Ions: both
Use MSn Info: yes
Isotope Res: 9000
Max Results: 200

Event#: 1 MS(E+) Ret. Time : 4.747 Scan# : 713

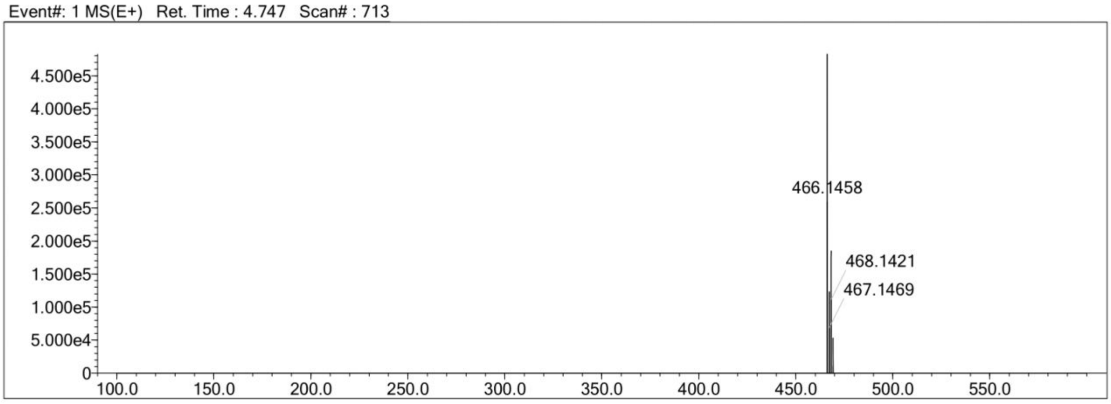

Measured region for 466.1458 m/z

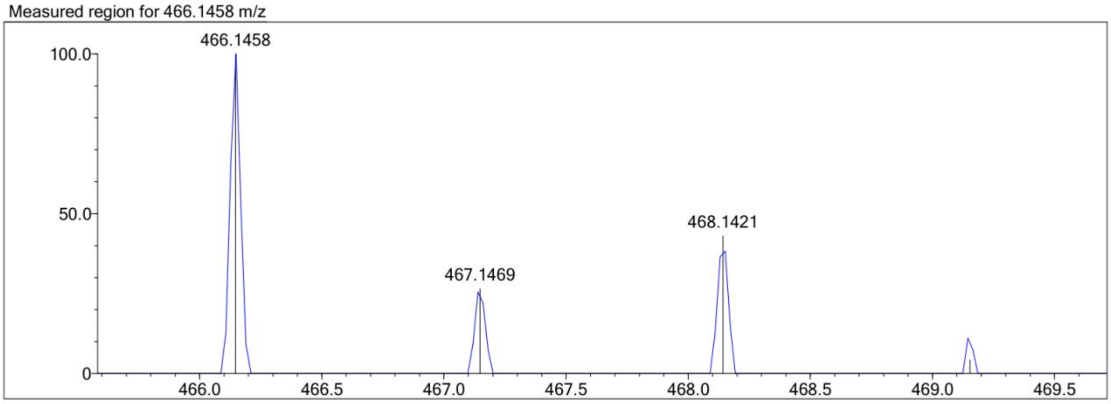

C24 H24 N5 O S Cl [M+H]+ : Predicted region for 466.1463 m/z

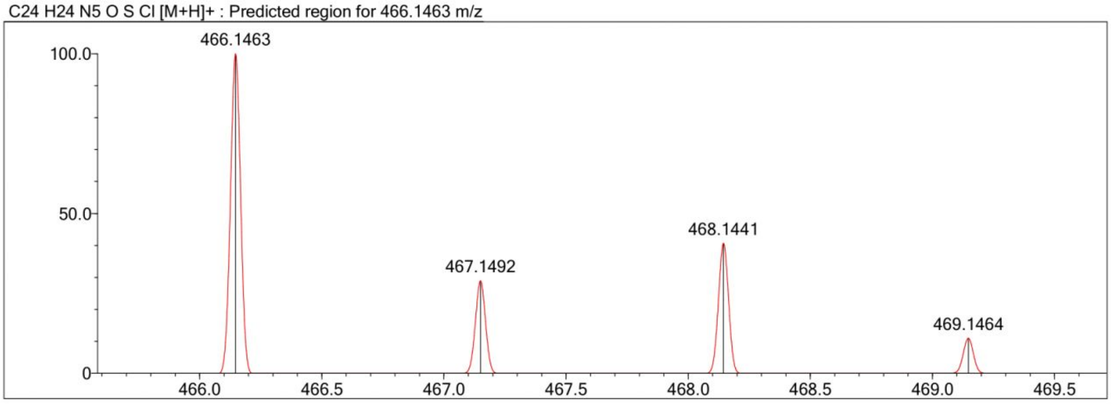

Rank	Score	Formula (M)	Ion	Meas. m/z	Pred. m/z	Df. (mDa)	Df. (ppm)	Iso	DBE
1	83.28	C24 H24 N5 O S Cl	[M+H]+	466.1458	466.1463	-0.5	-1.07	83.42	15.0

Figure S24. Compound **5f** HRMS report

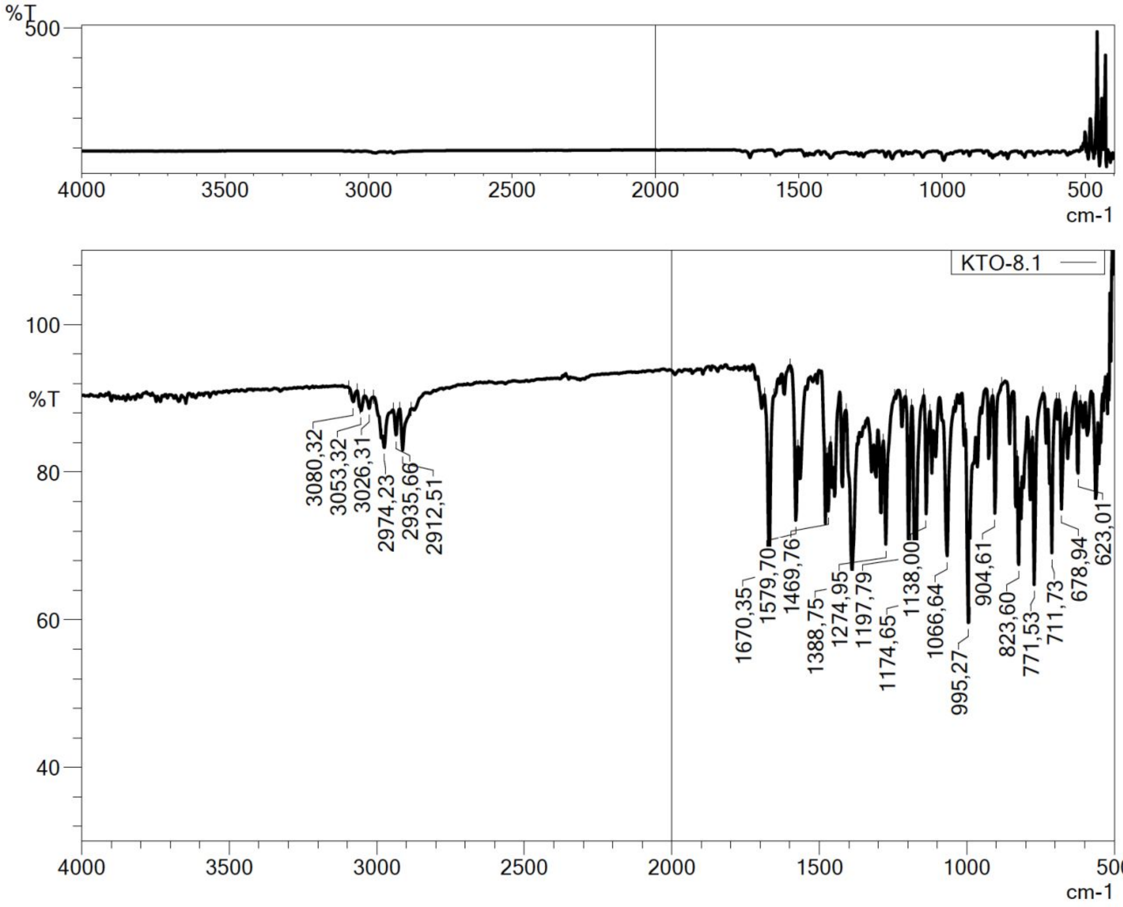

Figure S25. Compound **5g** IR report

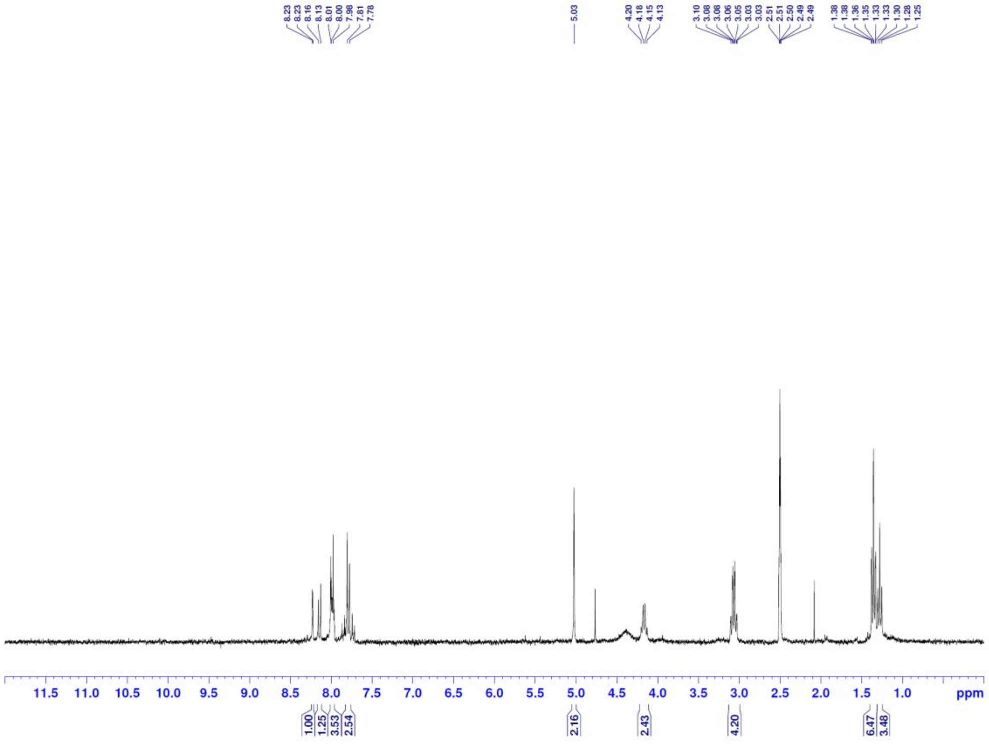

Figure S26. Compound **5g** ^{1}H-NMR spectrum

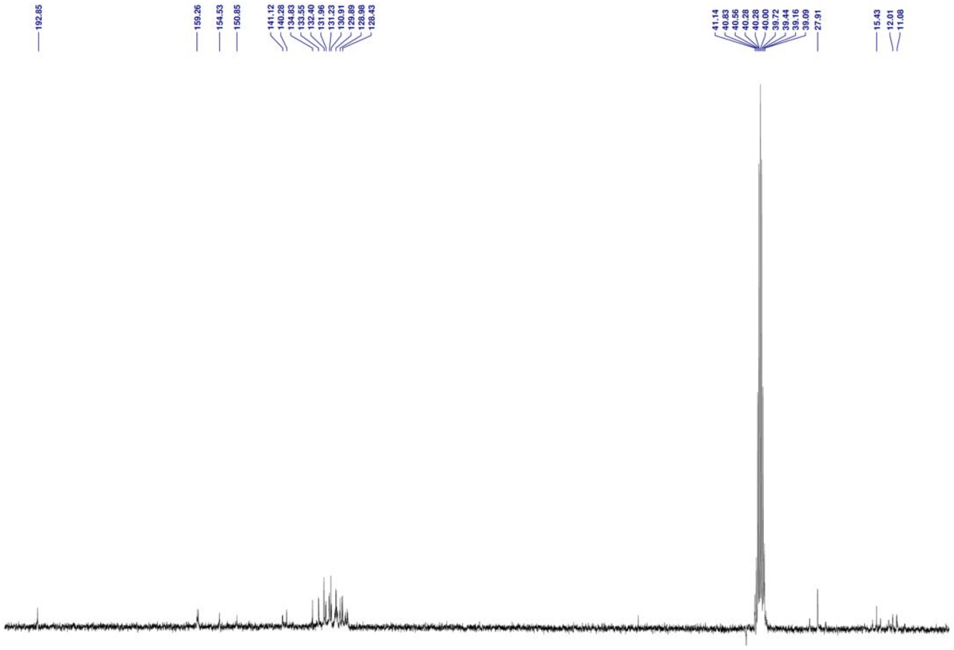

Figure S27. Compound **5g** ^{13}C-NMR spectrum

Formula Predictor Report - KTO-8_192.lcd

Data File: C:\LabSolutions\Data\Analiz\derya\KTO-8_192.lcd

Elmt	Val.	Min	Max	Elmt	Val.	Min	Max	Elmt	Val.	Min	Max	Elmt	Val.	Min	Max	Use Adduct
H	1	8	40	O	2	0	4	S	2	1	1	Ru	2	0	0	H
C	4	9	40	F	1	0	0	Cl	1	0	0	Pd	2	0	0	
N	3	2	6	P	3	0	0	Br	1	1	1	I	3	0	0	

Error Margin (ppm):	5	DBE Range:	5.0 - 25.0	Electron Ions:	both
HC Ratio:	unlimited	Apply N Rule:	yes	Use MSn Info:	yes
Max Isotopes:	3	Isotope RI (%):	1.00	Isotope Res:	9000
MSn Iso RI (%):	10.00	MSn Logic Mode:	AND	Max Results:	200

Event#: 1 MS(E+) Ret. Time : 5.520 Scan# : 829

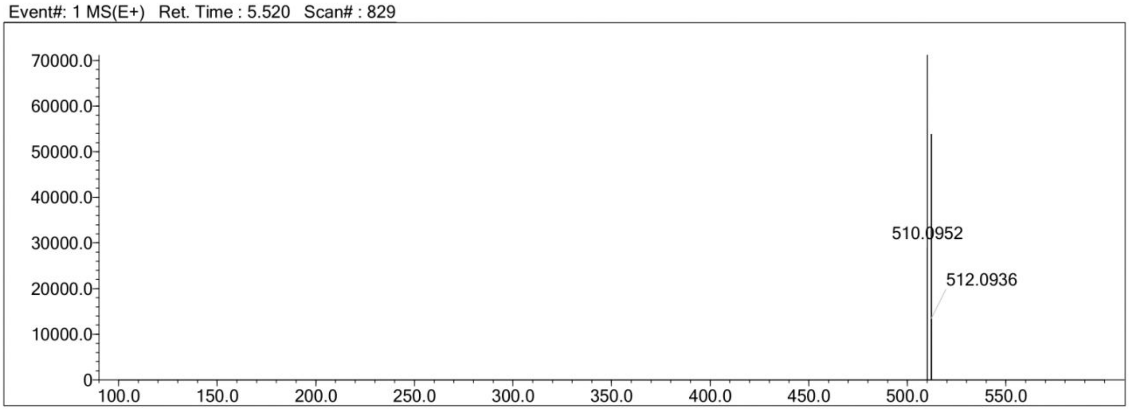

Measured region for 510.0952 m/z

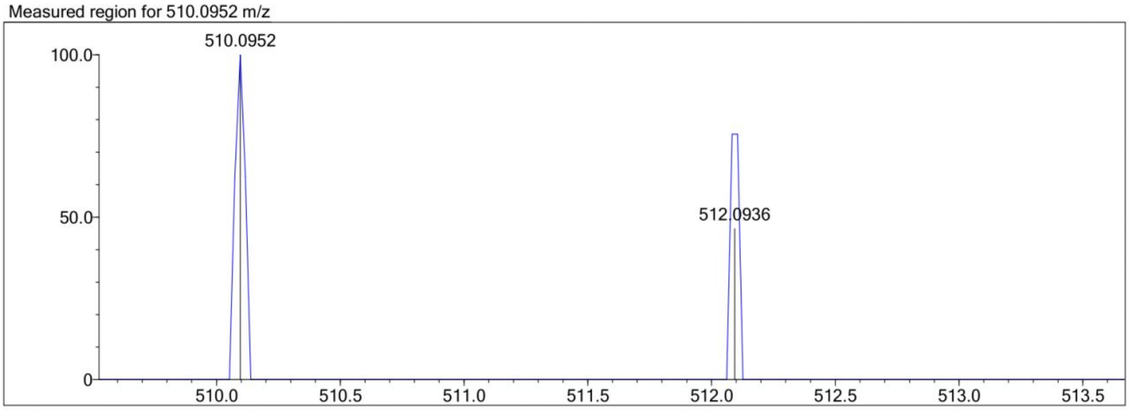

C24 H24 N5 O S Br [M+H]+ : Predicted region for 510.0958 m/z

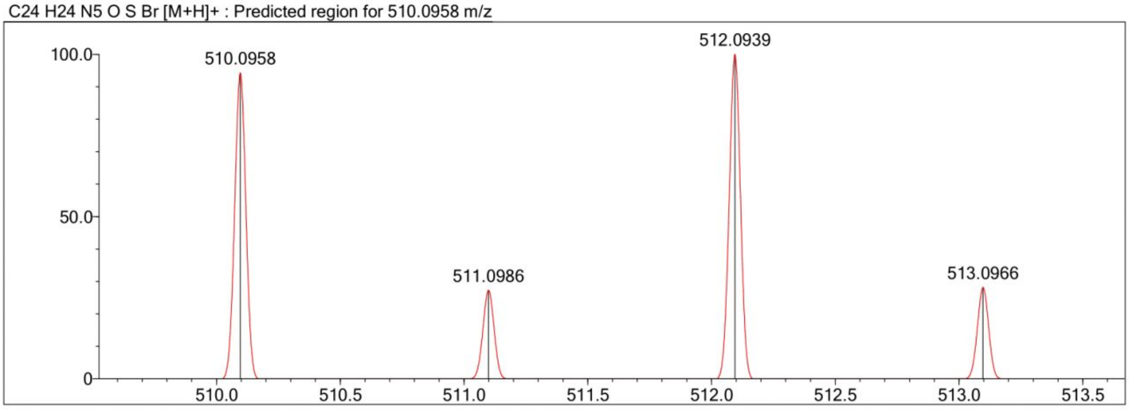

Rank	Score	Formula (M)	Ion	Meas. m/z	Pred. m/z	Df. (mDa)	Df. (ppm)	Iso	DBE
1	49.77	C24 H24 N5 O S Br	[M+H]+	510.0952	510.0958	-0.6	-1.18	50.00	15.0

Figure S28. Compound **5g** HRMS report

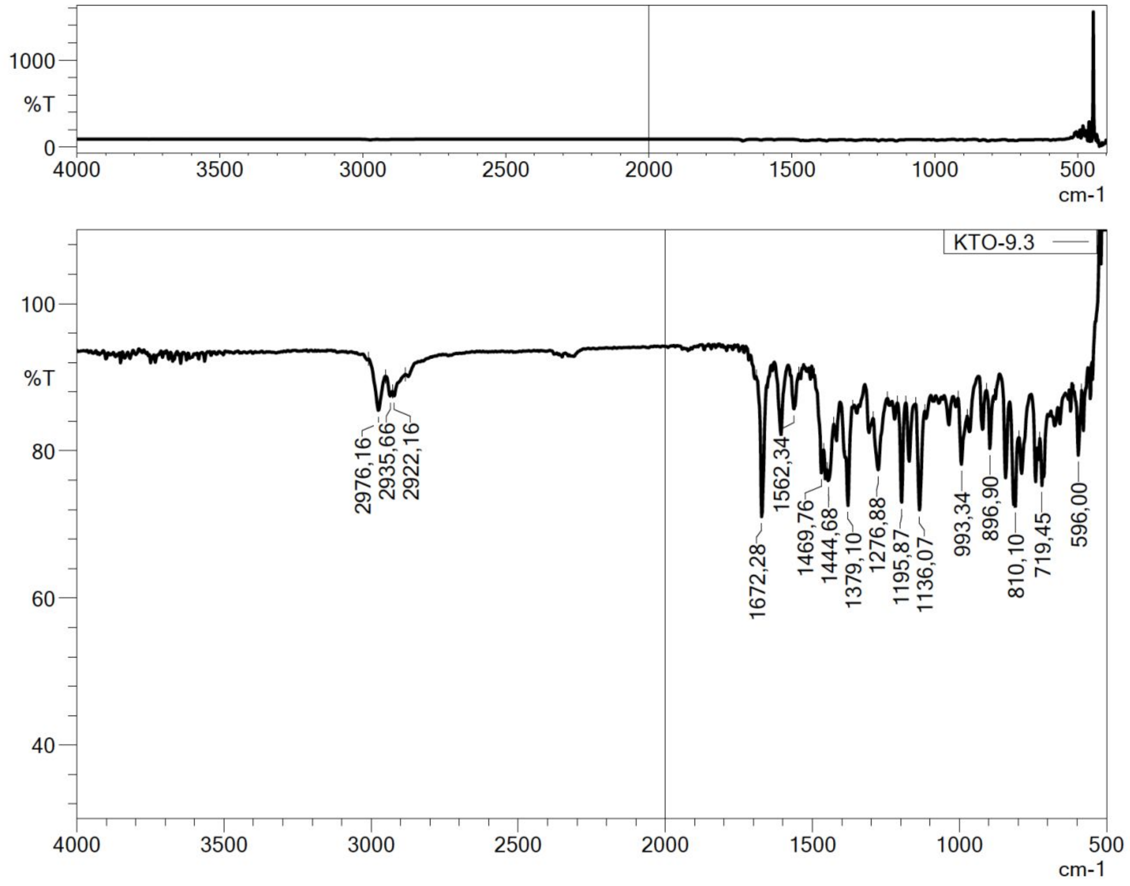

Figure S29. Compound **5h** IR report

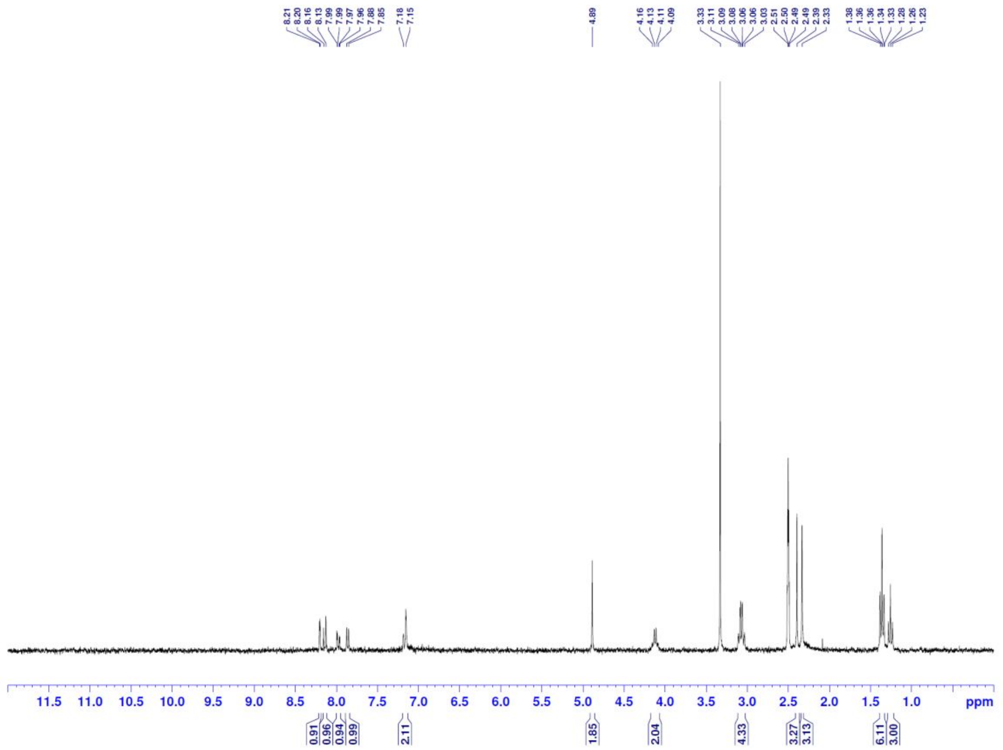

Figure S30. Compound **5h** ^{1}H-NMR spectrum

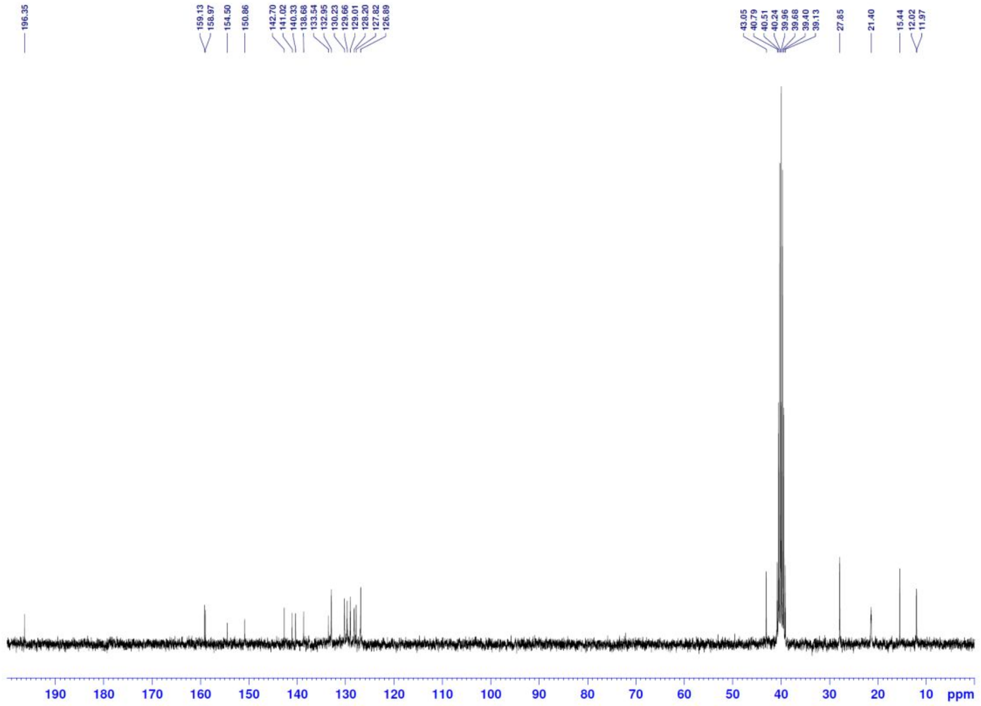

Figure S31. Compound **5h**^{13}C-NMR spectrum

Data File: C:\LabSolutions\Data\Analiz\derya\KTO-9_193.lcd

Elmt	Val.	Min	Max	Elmt	Val.	Min	Max	Elmt	Val.	Min	Max	Elmt	Val.	Min	Max	Use Adduct
H	1	8	40	O	2	0	4	S	2	1	1	Ru	2	0	0	H
C	4	9	40	F	1	0	0	Cl	1	0	0	Pd	2	0	0	
N	3	2	6	P	3	0	0	Br	1	0	0	I	3	0	0	

Error Margin (ppm):	5	DBE Range:	5.0 - 25.0	Electron Ions:	both
HC Ratio:	unlimited	Apply N Rule:	yes	Use MSn Info:	yes
Max Isotopes:	3	Isotope RI (%):	1.00	Isotope Res:	9000
MSn Iso RI (%):	10.00	MSn Logic Mode:	AND	Max Results:	200

Event#: 1 MS(E+) Ret. Time : 5.587 Scan# : 839

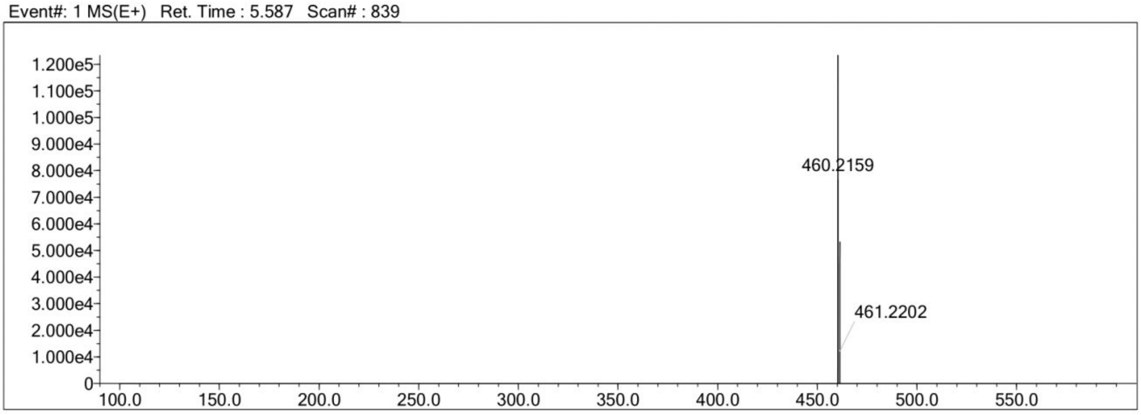

Measured region for 460.2159 m/z

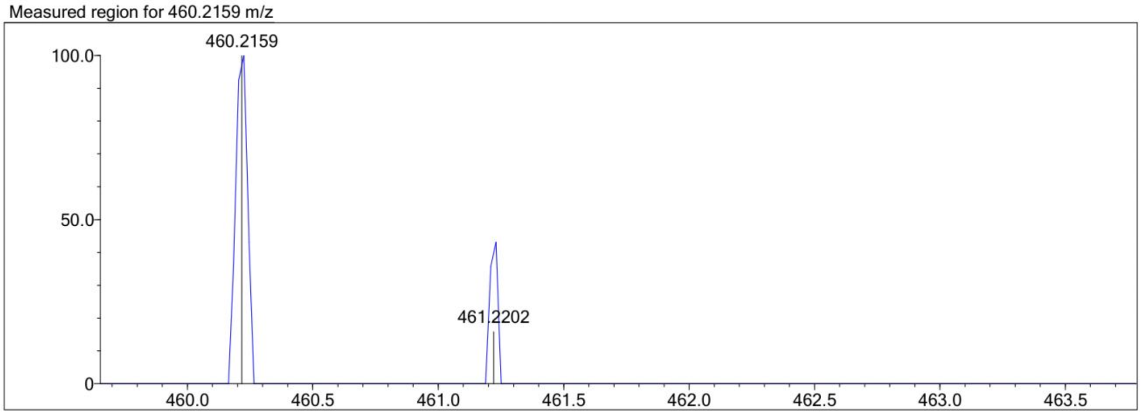

C26 H29 N5 O S [M+H]+ : Predicted region for 460.2166 m/z

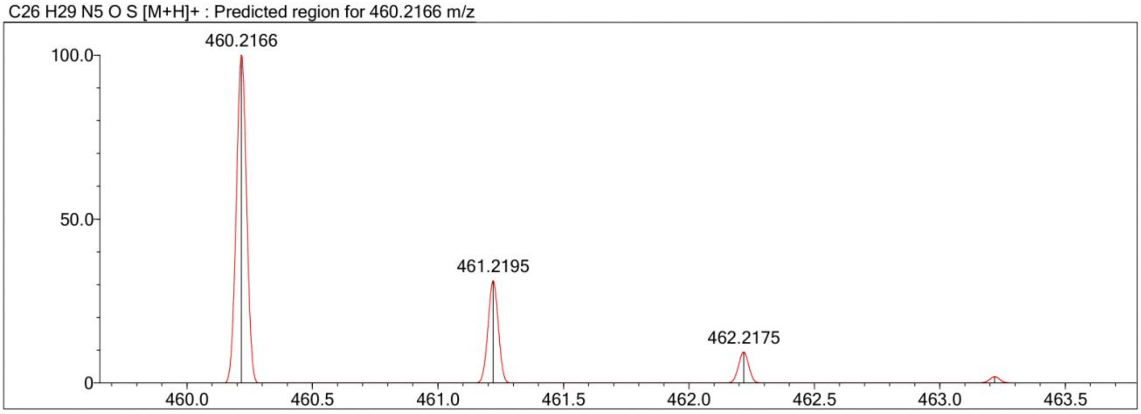

Rank	Score	Formula (M)	Ion	Meas. m/z	Pred. m/z	Df. (mDa)	Df. (ppm)	Iso	DBE
1	0.00	C26 H29 N5 O S	[M+H]+	460.2159	460.2166	-0.7	-1.52	0.00	15.0

Figure S32. Compound **5h** HRMS report

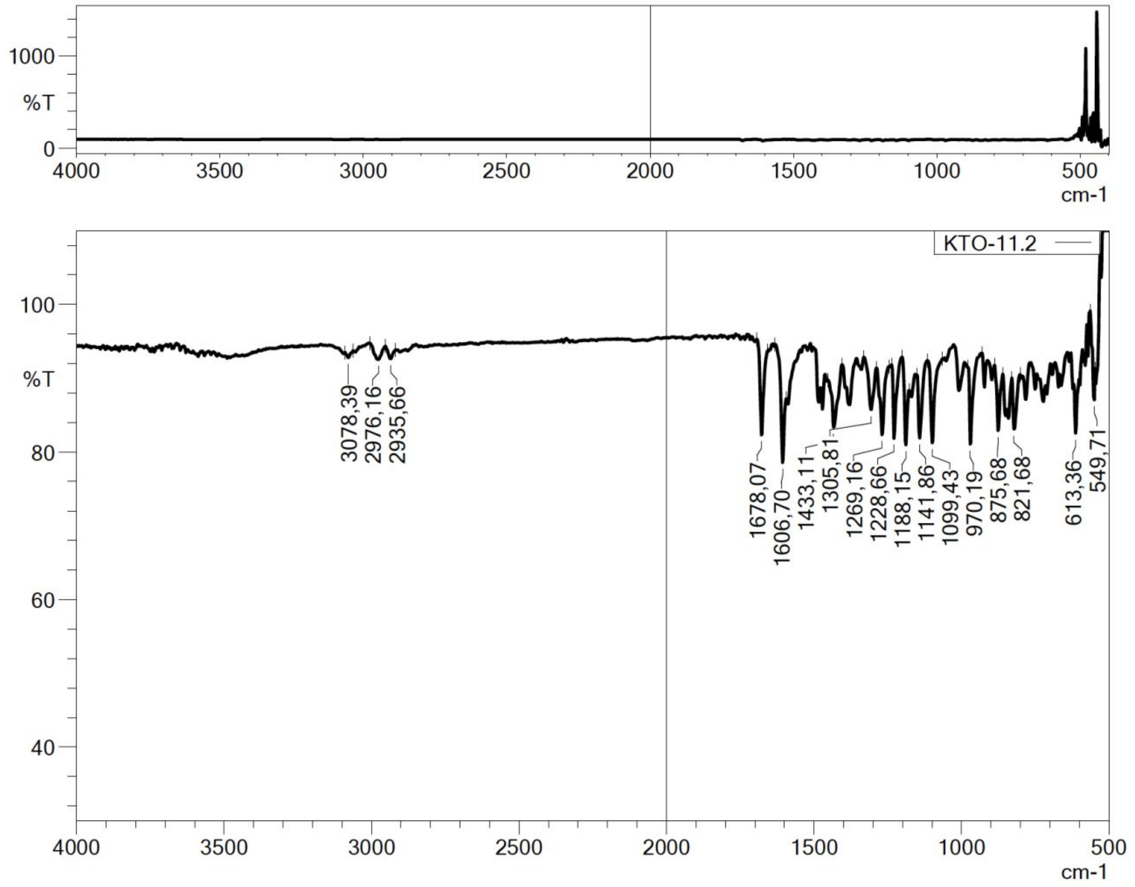

Figure S33. Compound **5i** IR report

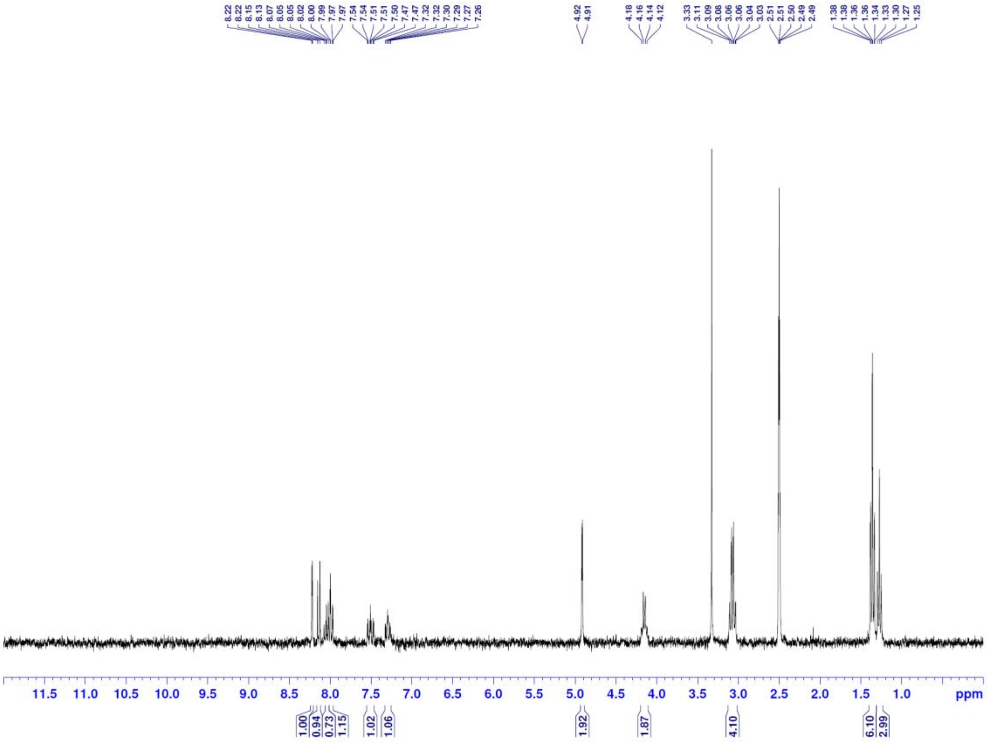

Figure S34. Compound **5i** ^{1}H-NMR spectrum

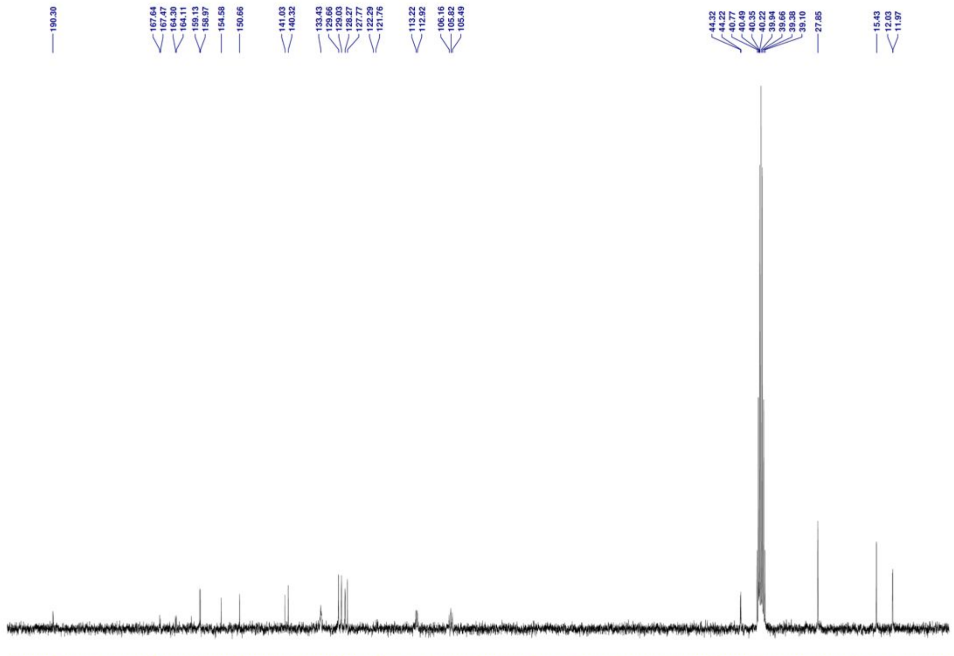

Figure S35. Compound **5i** ^{13}C-NMR spectrum

Data File: C:\LabSolutions\Data\Analiz\derya\KTO-11_194.lcd

Elmt	Val.	Min	Max	Elmt	Val.	Min	Max	Elmt	Val.	Min	Max	Elmt	Val.	Min	Max	Use Adduct
H	1	8	40	O	2	0	4	S	2	1	1	Ru	2	0	0	H
C	4	9	40	F	1	2	2	Cl	1	0	0	Pd	2	0	0	
N	3	2	6	P	3	0	0	Br	1	0	0	I	3	0	0	

Error Margin (ppm):	5	DBE Range:	5.0 - 25.0	Electron Ions:	both
HC Ratio:	unlimited	Apply N Rule:	yes	Use MSn Info:	yes
Max Isotopes:	3	Isotope RI (%):	1.00	Isotope Res:	9000
MSn Iso RI (%):	10.00	MSn Logic Mode:	AND	Max Results:	200

Event#: 1 MS(E+) Ret. Time : 4.133 Scan# : 621

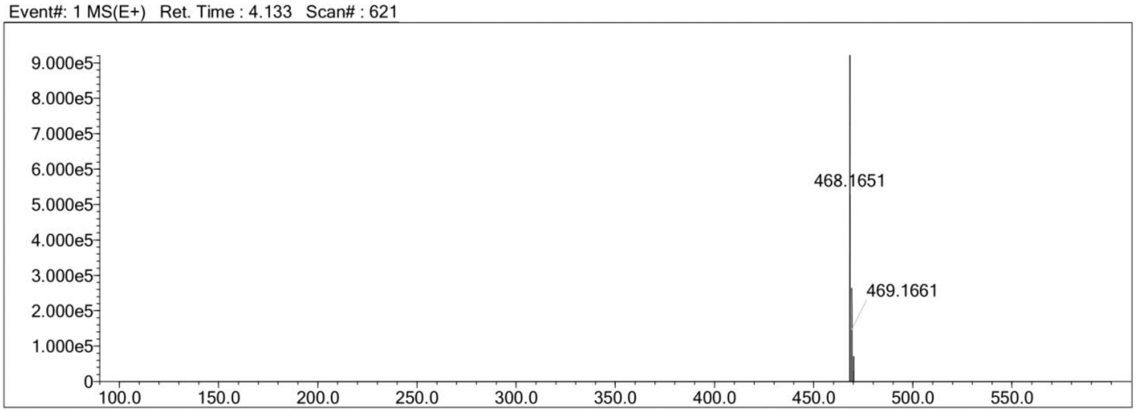

Measured region for 468.1651 m/z

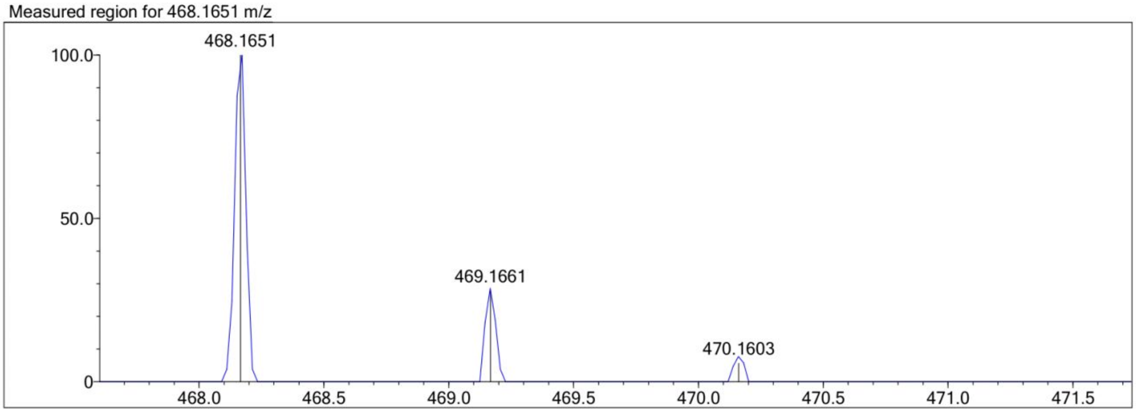

C24 H23 N5 O F2 S [M+H]+ : Predicted region for 468.1664 m/z

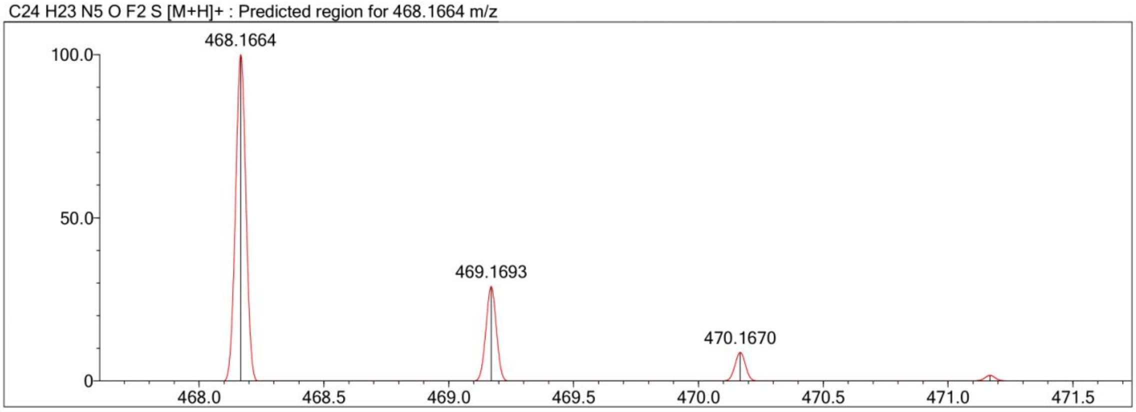

Rank	Score	Formula (M)	Ion	Meas. m/z	Pred. m/z	Df. (mDa)	Df. (ppm)	Iso	DBE
1	72.92	C24 H23 N5 O F2 S	[M+H]+	468.1651	468.1664	-1.3	-2.78	76.31	15.0

Figure S36. Compound **5i** HRMS report

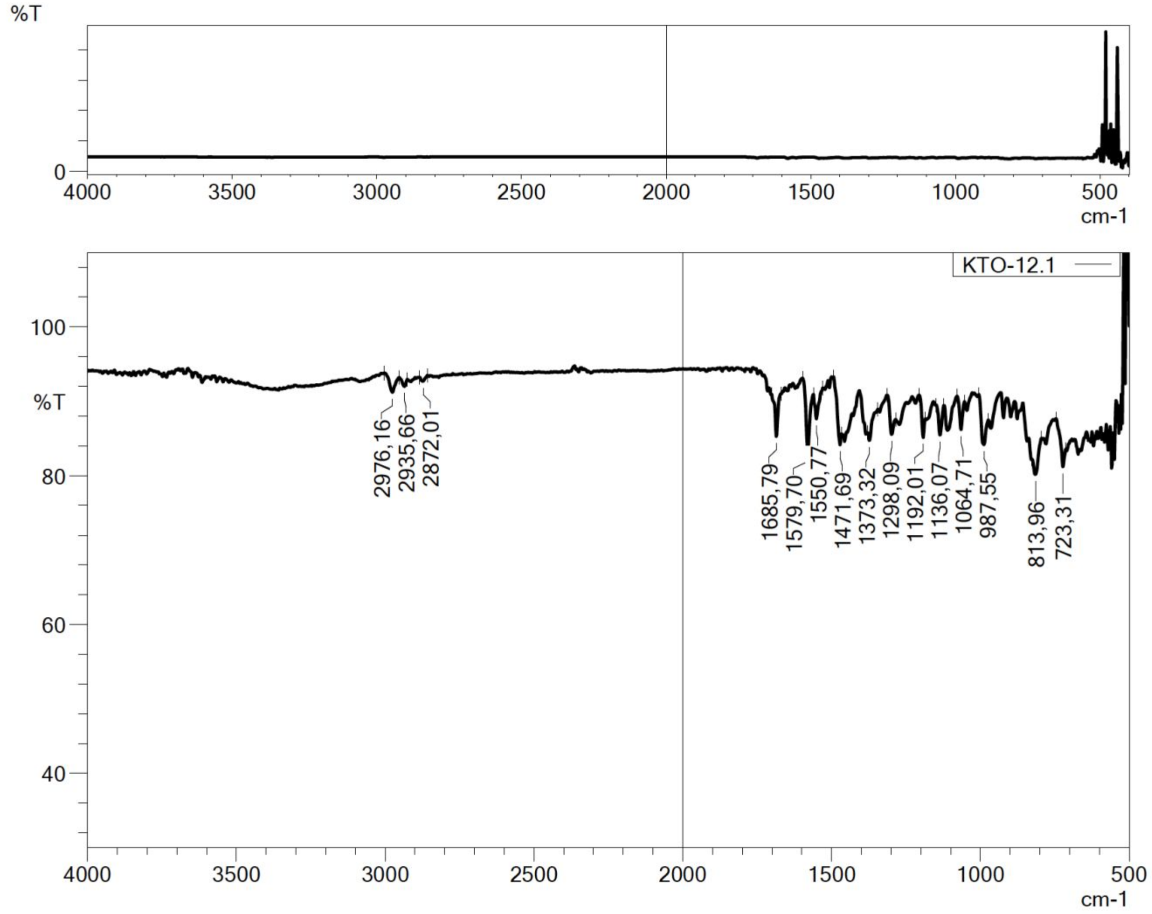

Figure S37. Compound **5j** IR report

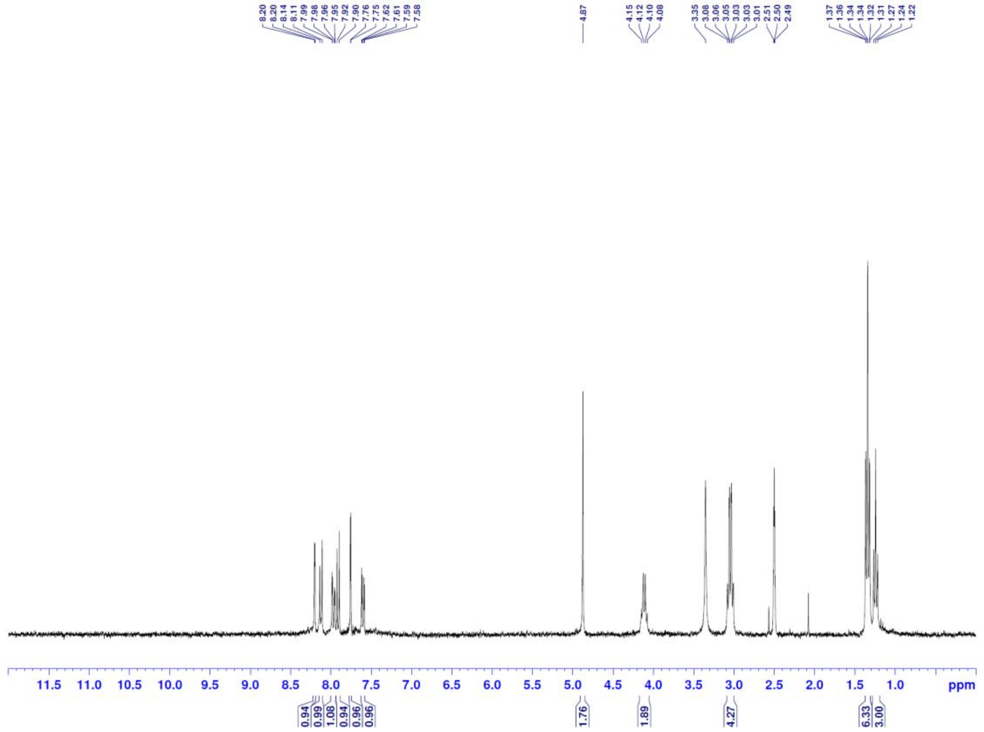

Figure S38. Compound **5j** ^{1}H-NMR spectrum

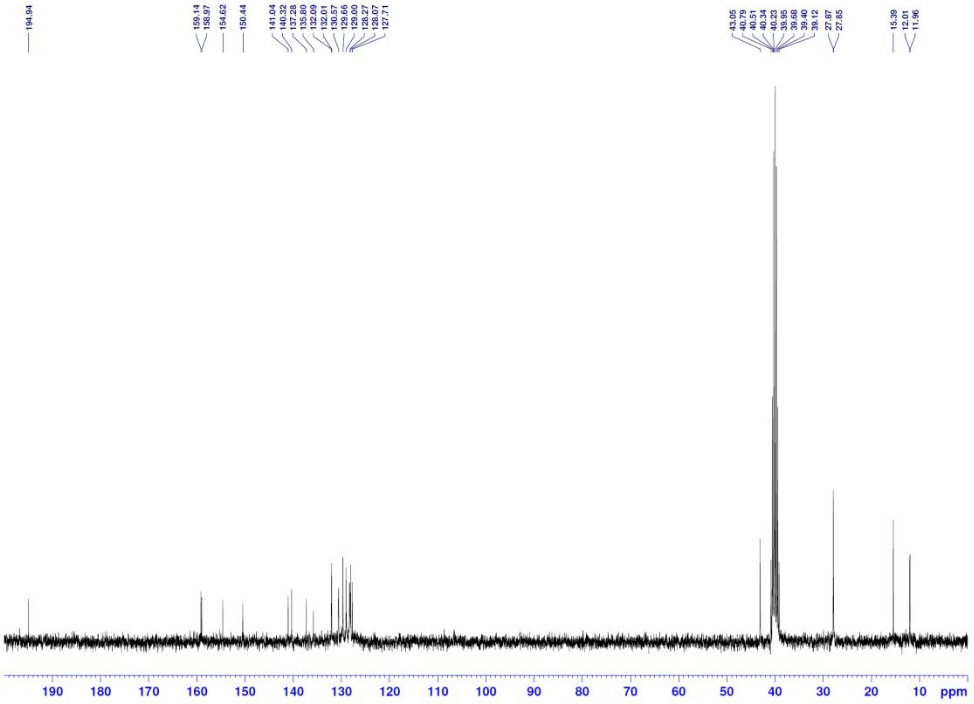

Figure S39. Compound **5j** ^{13}C-NMR spectrum

Data File: C:\LabSolutions\Data\Analiz\derya\KTO-12_195.lcd

Elmt	Val.	Min	Max	Elmt	Val.	Min	Max	Elmt	Val.	Min	Max	Elmt	Val.	Min	Max	Use Adduct
H	1	8	40	O	2	0	4	S	2	1	1	Ru	2	0	0	H
C	4	9	40	F	1	0	0	Cl	1	2	2	Pd	2	0	0	
N	3	2	6	P	3	0	0	Br	1	0	0	I	3	0	0	

Error Margin (ppm):	5	DBE Range:	5.0 - 25.0	Electron Ions:	both
HC Ratio:	unlimited	Apply N Rule:	yes	Use MSn Info:	yes
Max Isotopes:	3	Isotope RI (%):	1.00	Isotope Res:	9000
MSn Iso RI (%):	10.00	MSn Logic Mode:	AND	Max Results:	200

Event#: 1 MS(E+) Ret. Time : 6.453 -> 6.453 Scan# : 969 -> 969

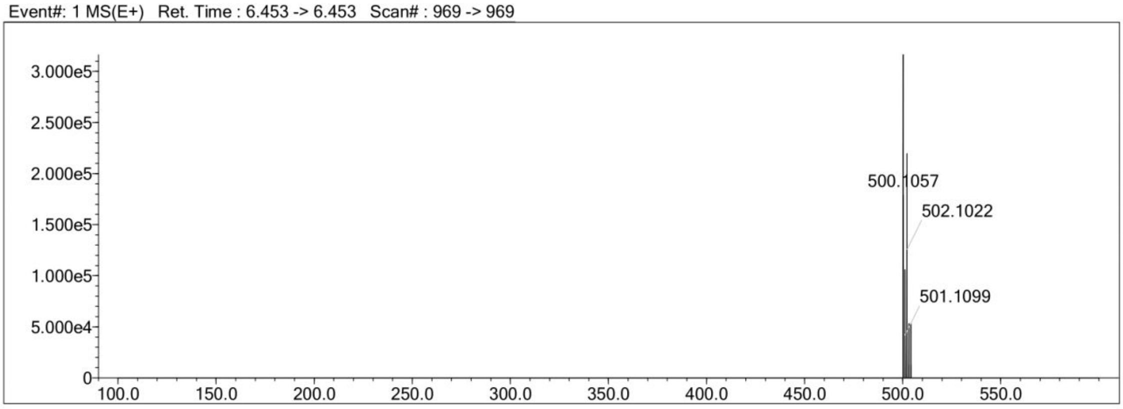

Measured region for 500.1057 m/z

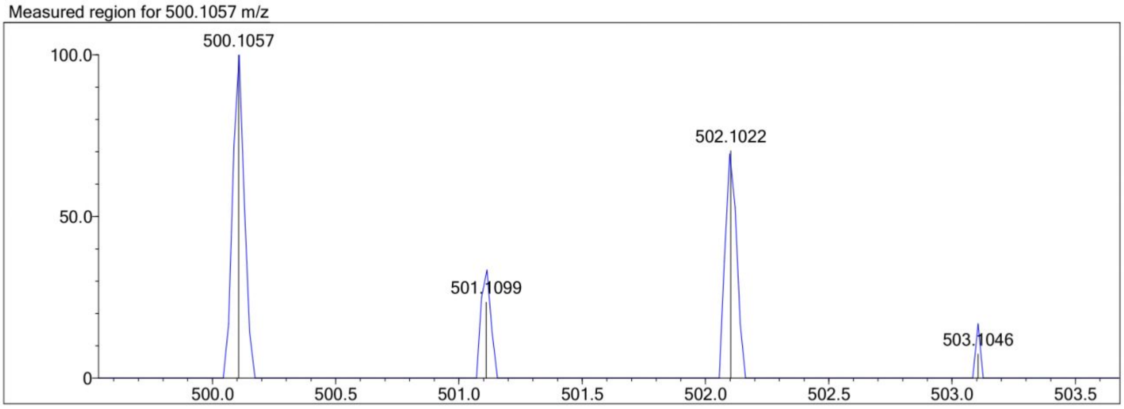

C24 H23 N5 O S Cl2 [M+H]+ : Predicted region for 500.1073 m/z

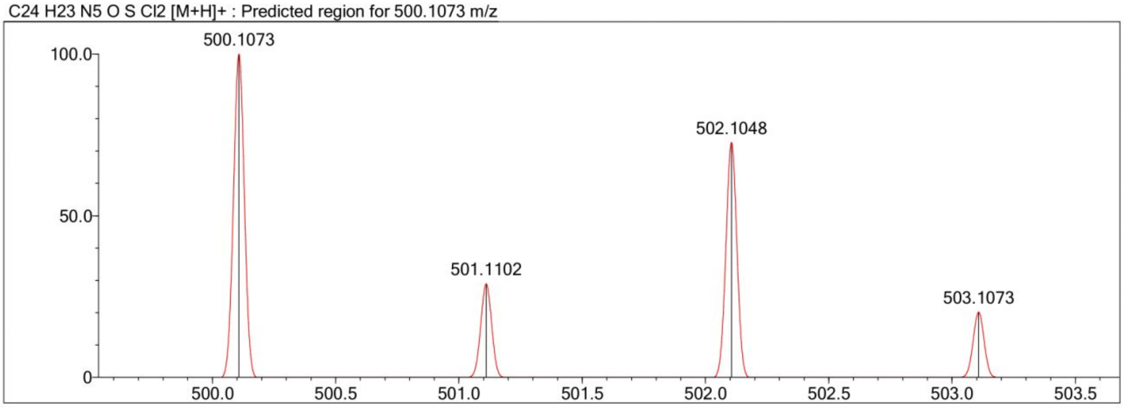

Rank	Score	Formula (M)	Ion	Meas. m/z	Pred. m/z	Df. (mDa)	Df. (ppm)	Iso	DBE
1	69.00	C24 H23 N5 O S Cl2	[M+H]+	500.1057	500.1073	-1.6	-3.20	73.02	15.0

Figure S40. Compound **5j** HRMS report

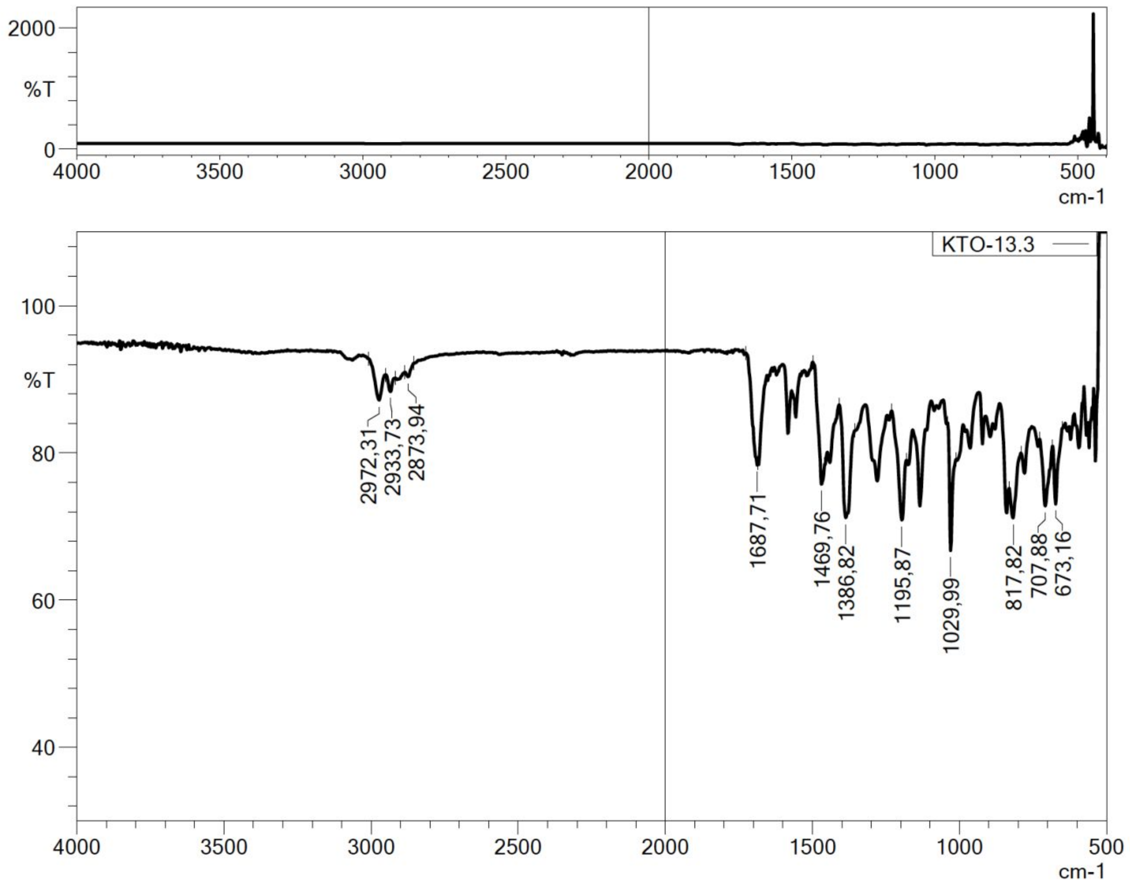

Figure S41. Compound **5k** IR report

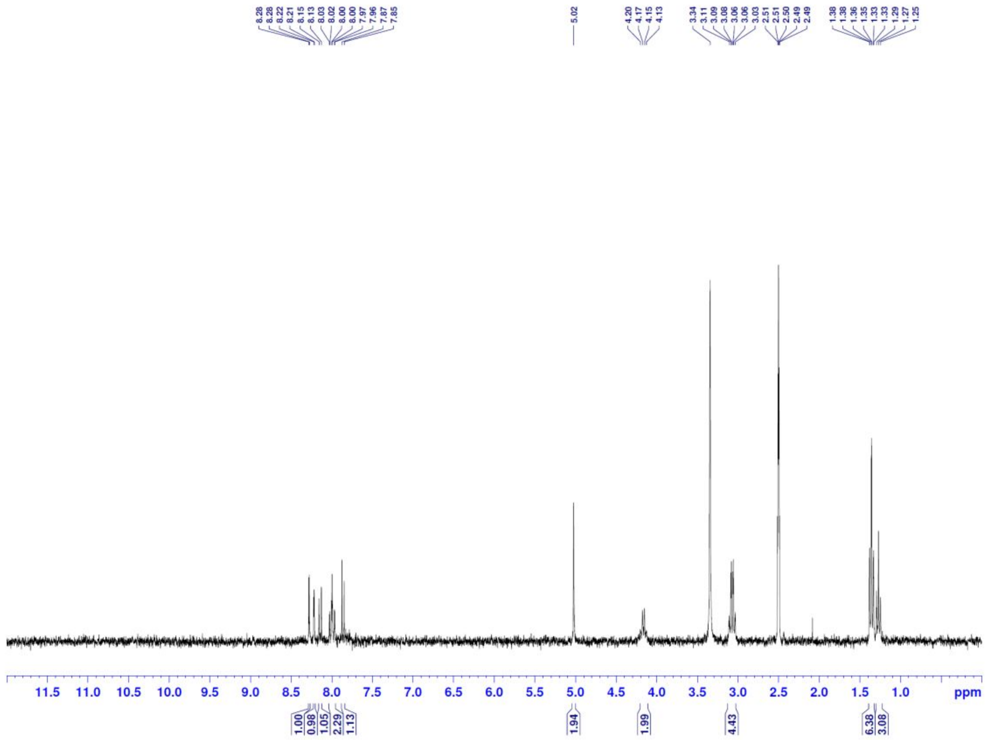

Figure S42. Compound **5k** ^{1}H-NMR spectrum

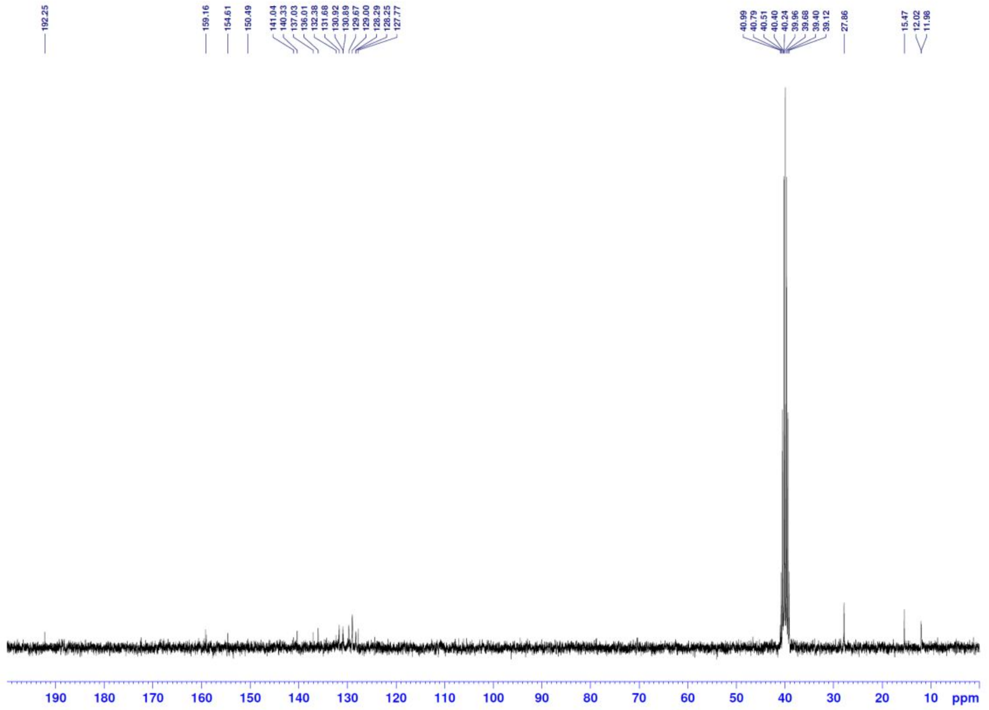

Figure S43. Compound **5k** ^{13}C-NMR spectrum

Formula Predictor Report - KTO-13_196.lcd

Data File: C:\LabSolutions\Data\Analiz\derya\KTO-13_196.lcd

Elmt	Val.	Min	Max	Elmt	Val.	Min	Max	Elmt	Val.	Min	Max	Elmt	Val.	Min	Max	Use Adduct
H	1	8	40	O	2	0	4	S	2	1	1	Ru	2	0	0	H
C	4	9	40	F	1	0	0	Cl	1	2	2	Pd	2	0	0	
N	3	2	6	P	3	0	0	Br	1	0	0	I	3	0	0	

Error Margin (ppm):	5	DBE Range:	5.0 - 25.0	Electron Ions:	both
HC Ratio:	unlimited	Apply N Rule:	yes	Use MSn Info:	yes
Max Isotopes:	3	Isotope RI (%):	1.00	Isotope Res:	9000
MSn Iso RI (%):	10.00	MSn Logic Mode:	AND	Max Results:	200

Event#: 1 MS(E+) Ret. Time : 6.267 Scan# : 941

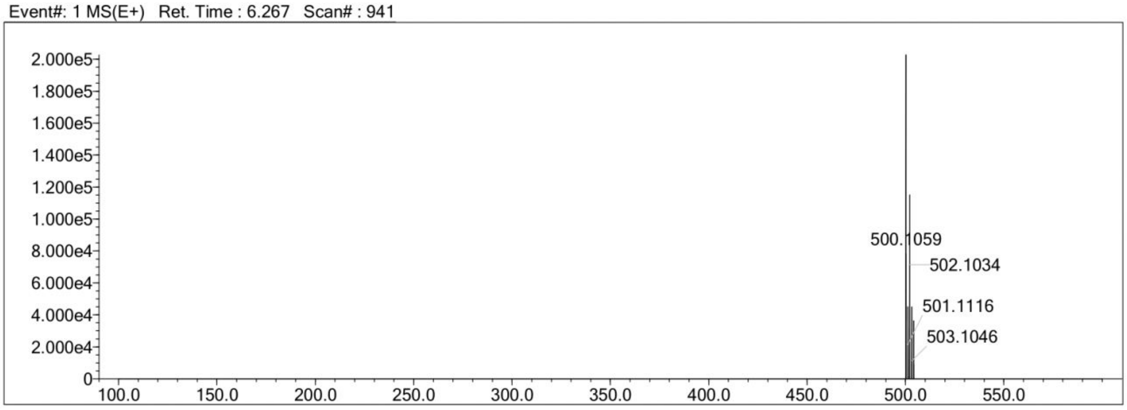

Measured region for 500.1059 m/z

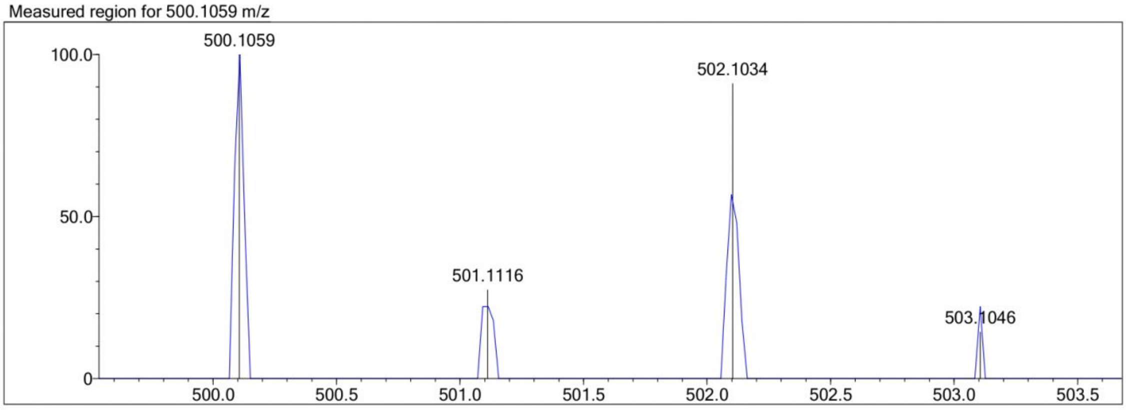

C24 H23 N5 O S Cl2 [M+H]+ : Predicted region for 500.1073 m/z

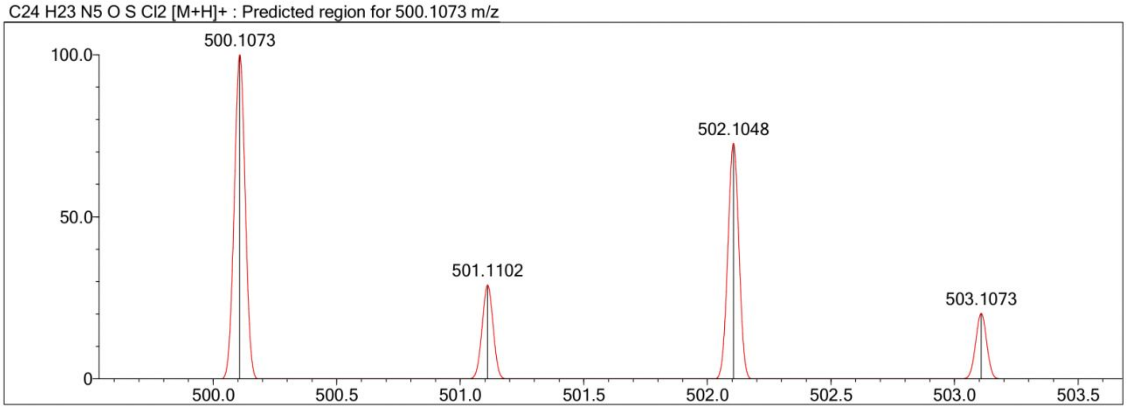

Rank	Score	Formula (M)	Ion	Meas. m/z	Pred. m/z	Df. (mDa)	Df. (ppm)	Iso	DBE
1	62.99	C24 H23 N5 O S Cl2	[M+H]+	500.1059	500.1073	-1.4	-2.80	65.96	15.0

Figure S44. Compound **5k** HRMS report

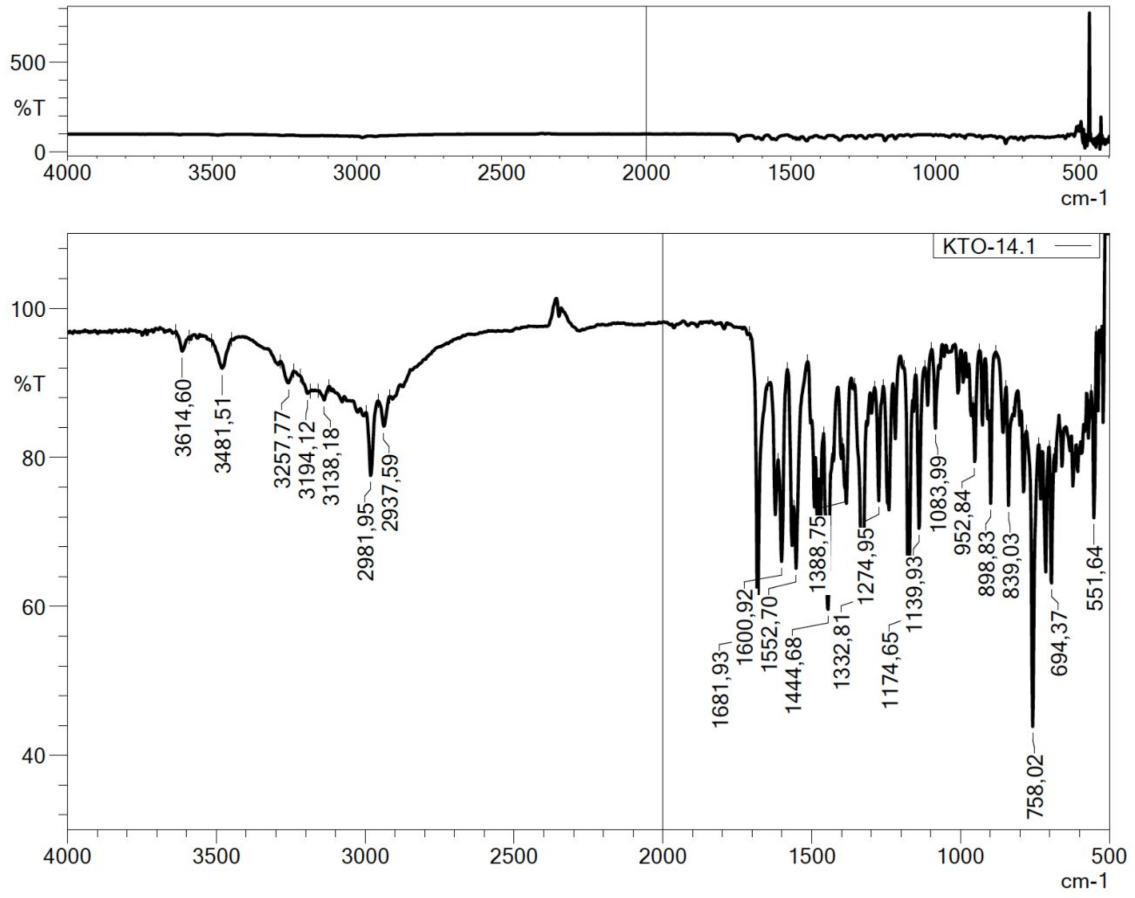

Figure S45. Compound **7a** IR report

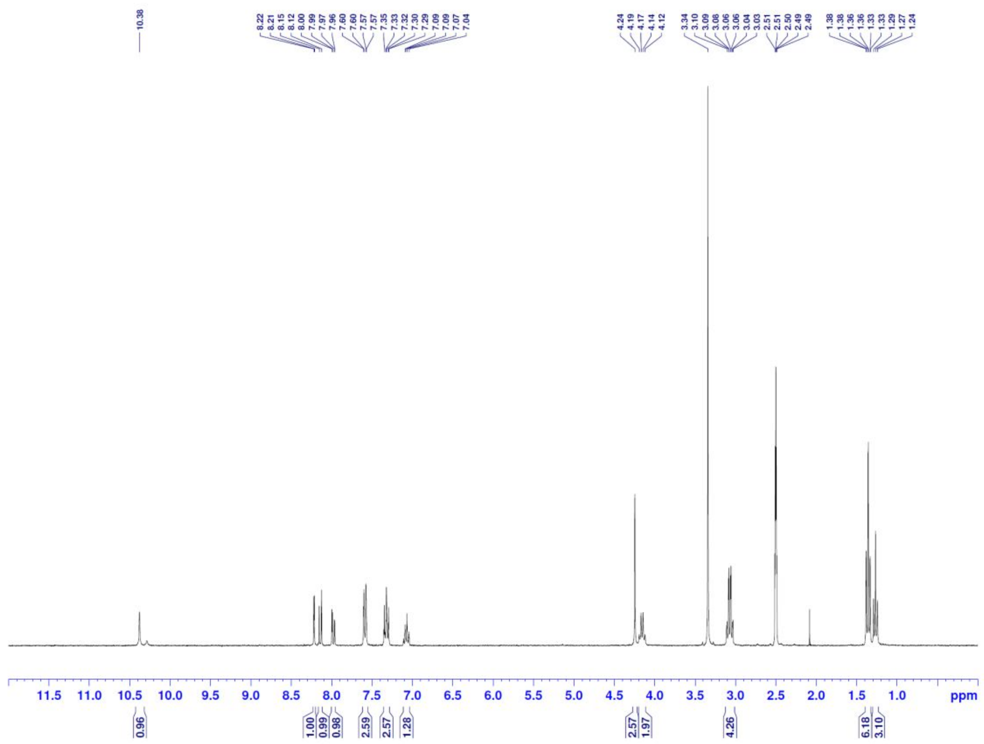

Figure S46. Compound **7a** ^{1}H-NMR spectrum

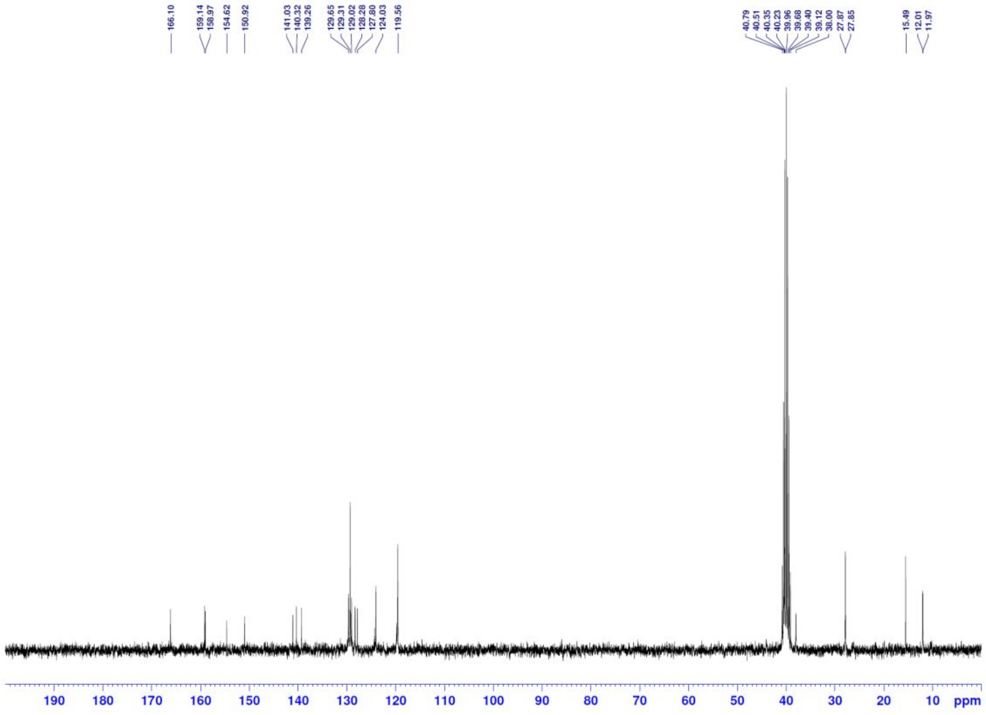

Figure S47. Compound **7a** ^{13}C-NMR spectrum

Data File: C:\LabSolutions\Data\Analiz\derya\KTO-14_197.lcd

Elmt	Val.	Min	Max	Elmt	Val.	Min	Max	Elmt	Val.	Min	Max	Elmt	Val.	Min	Max	Use Adduct
H	1	8	40	O	2	0	4	S	2	1	1	Ru	2	0	0	H
C	4	9	40	F	1	0	0	Cl	1	0	0	Pd	2	0	0	
N	3	2	6	P	3	0	0	Br	1	0	0	I	3	0	0	

Error Margin (ppm):	5	DBE Range:	5.0 - 25.0	Electron Ions:	both
HC Ratio:	unlimited	Apply N Rule:	yes	Use MSn Info:	yes
Max Isotopes:	3	Isotope RI (%):	1.00	Isotope Res:	9000
MSn Iso RI (%):	10.00	MSn Logic Mode:	AND	Max Results:	200

Event#: 1 MS(E+) Ret. Time : 3.427 Scan# : 515

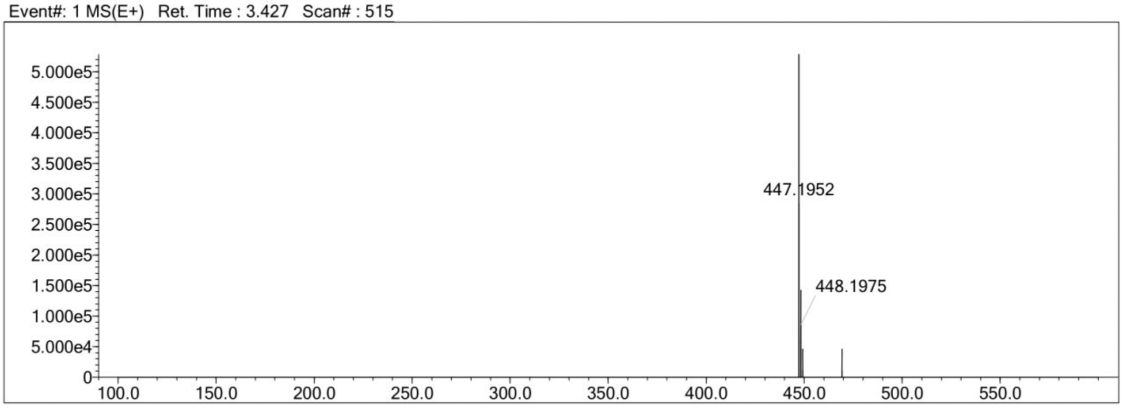

Measured region for 447.1952 m/z

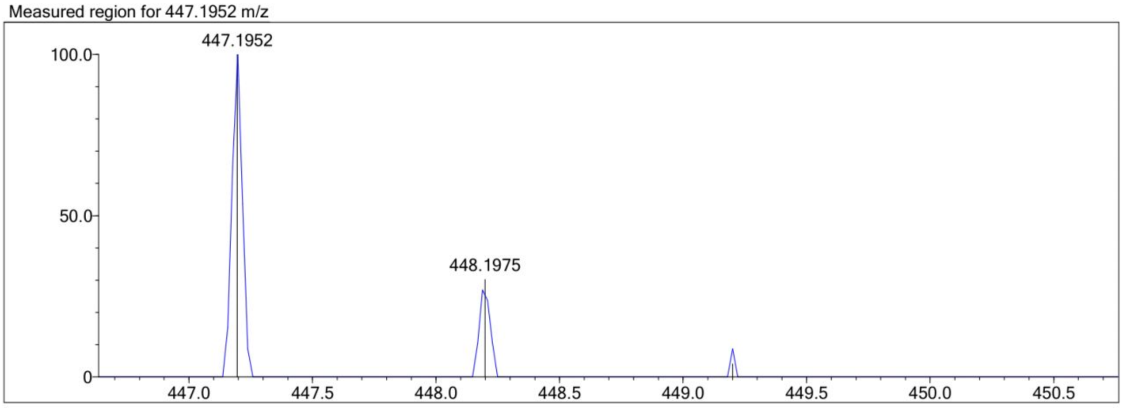

C24 H26 N6 O S [M+H]+ : Predicted region for 447.1962 m/z

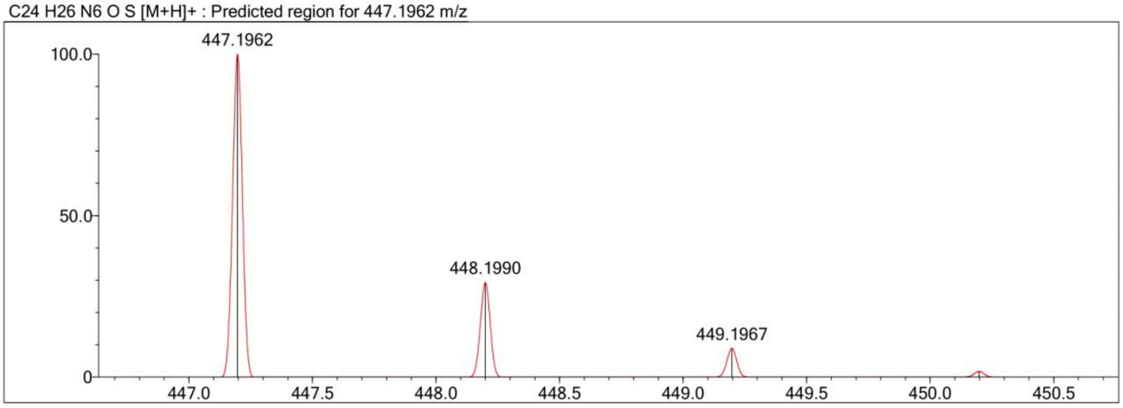

Rank	Score	Formula (M)	Ion	Meas. m/z	Pred. m/z	Df. (mDa)	Df. (ppm)	Iso	DBE
1	83.62	C24 H26 N6 O S	[M+H]+	447.1952	447.1962	-1.0	-2.24	86.30	15.0

Figure S48. Compound **7a** HRMS report

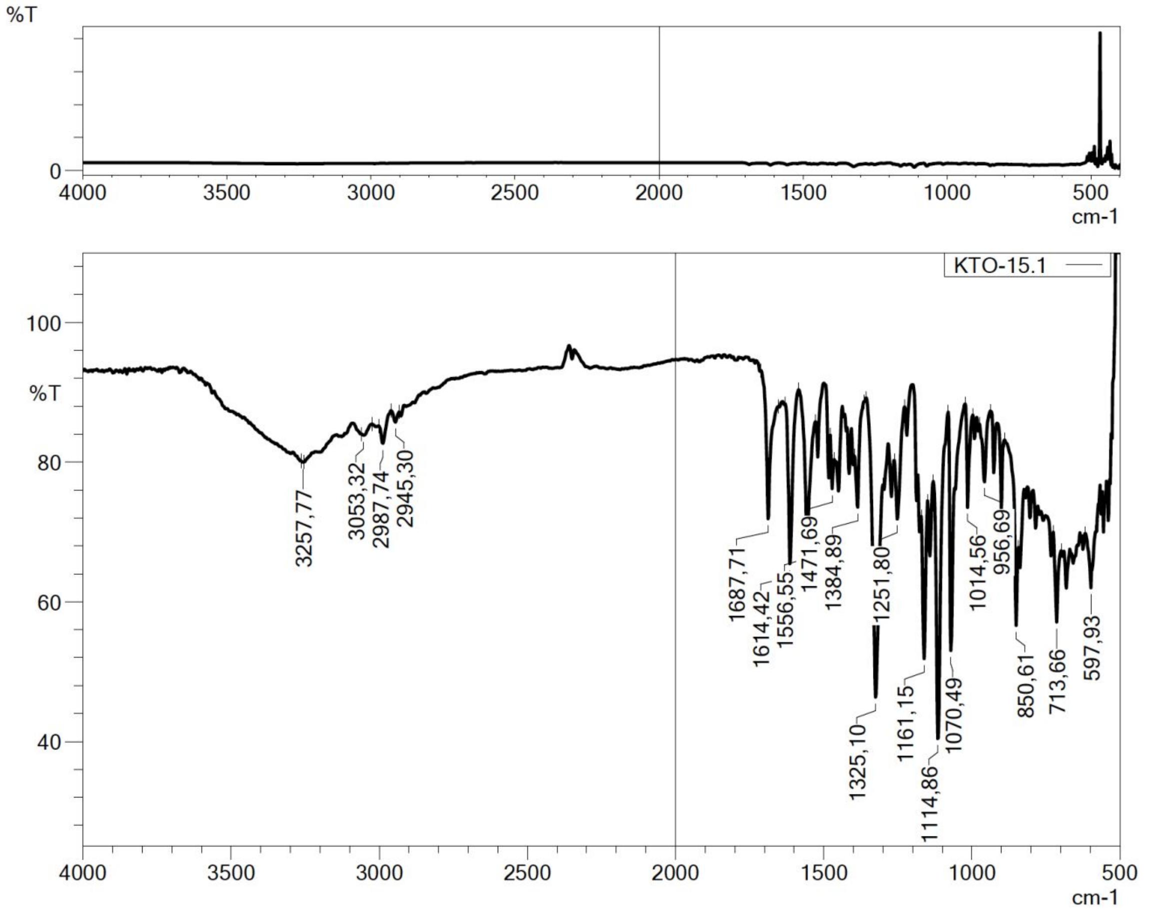

Figure S49. Compound **7b** IR report

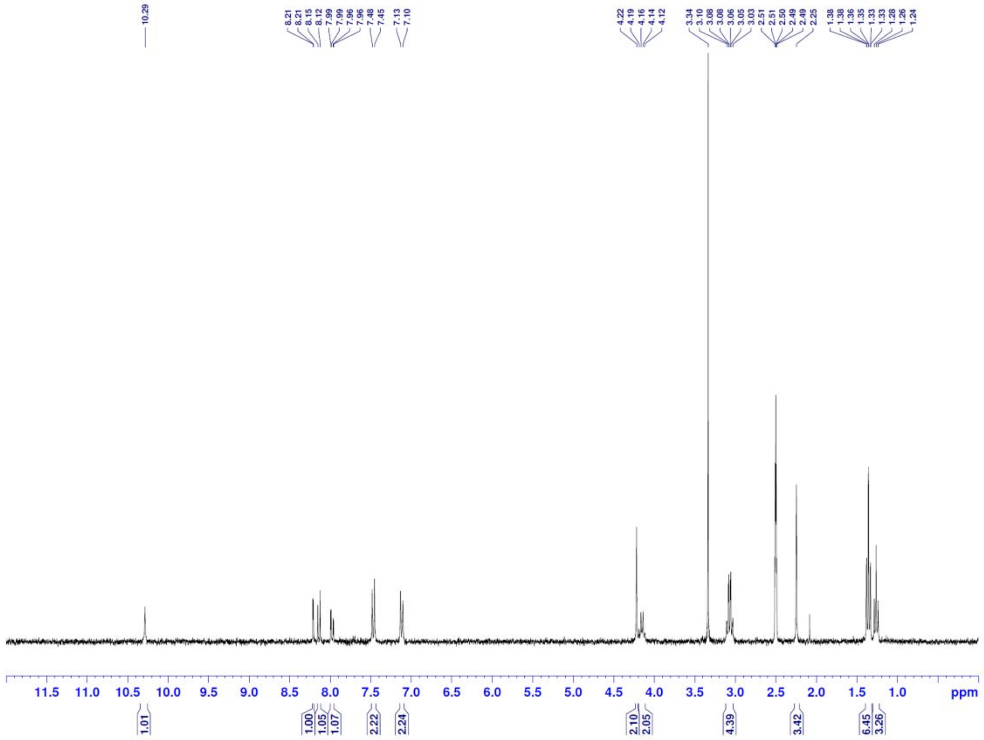

Figure S50. Compound **7b** ^{1}H-NMR spectrum

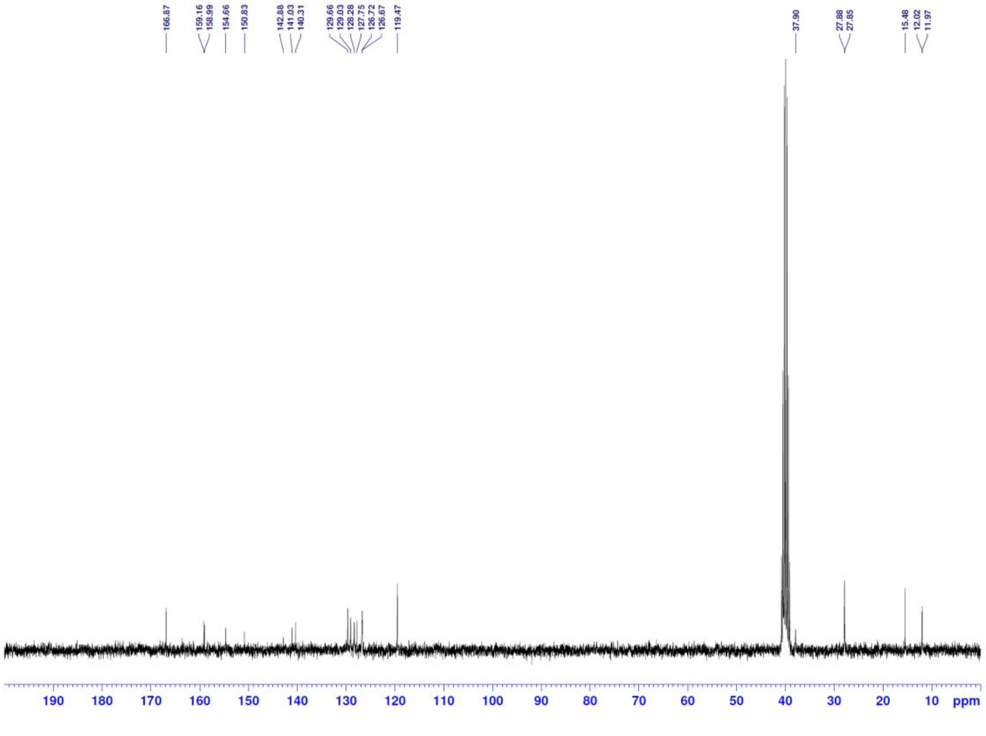

Figure S51. Compound **7b** ^{13}C-NMR spectrum

Data File: C:\LabSolutions\Data\Analiz\derya\KTO-19_202.lcd

Elmt	Val.	Min	Max	Elmt	Val.	Min	Max	Elmt	Val.	Min	Max	Elmt	Val.	Min	Max	Use Adduct
H	1	6	46	O	2	0	6	S	2	1	1	Ru	2	0	0	H
C	4	5	36	F	1	0	0	Cl	1	0	1	Pd	2	0	0	
N	3	0	6	P	3	0	0	Br	1	0	0	I	3	0	0	

Error Margin (ppm):	5	DBE Range:	11.0 - 30.0	Electron Ions:	both
HC Ratio:	unlimited	Apply N Rule:	yes	Use MSn Info:	yes
Max Isotopes:	3	Isotope RI (%):	1.00	Isotope Res:	9000
MSn Iso RI (%):	10.00	MSn Logic Mode:	AND	Max Results:	50

Event#: 1 MS(E+) Ret. Time : 3.613 Scan# : 543

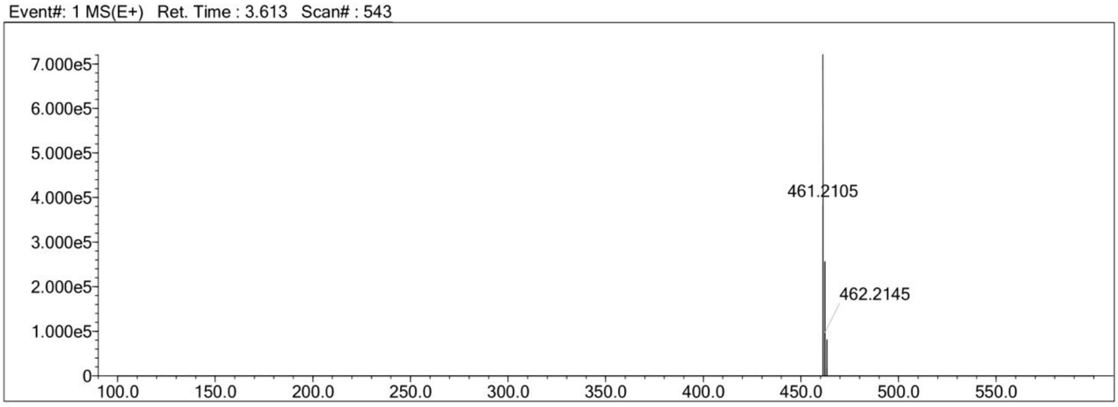

Measured region for 461.2105 m/z

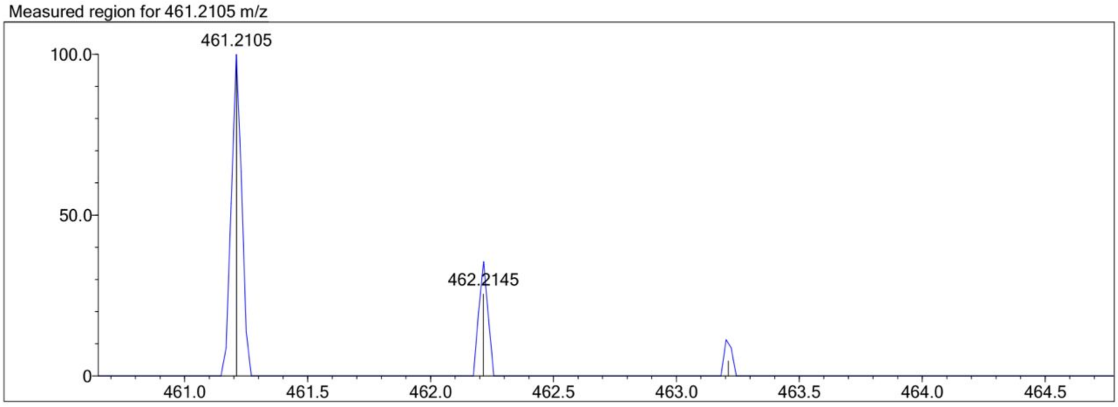

C25 H28 N6 O S [M+H]+ : Predicted region for 461.2118 m/z

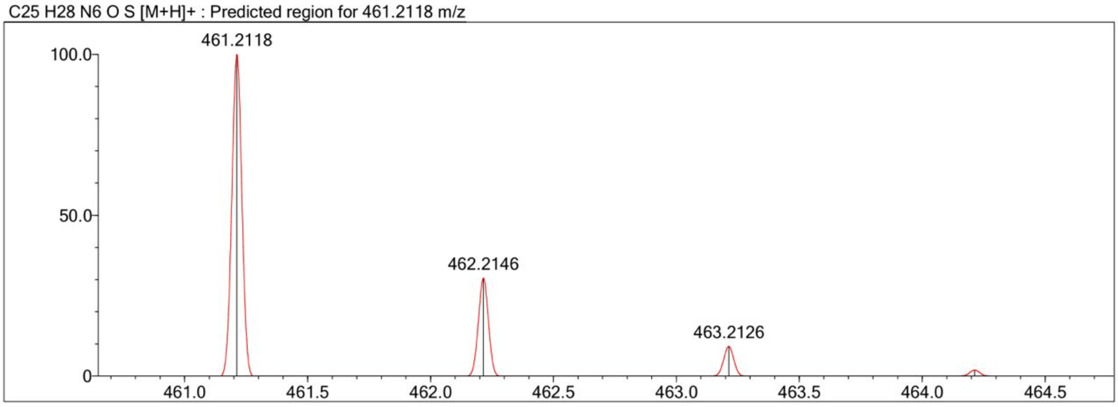

Rank	Score	Formula (M)	Ion	Meas. m/z	Pred. m/z	Df. (mDa)	Df. (ppm)	Iso	DBE
1	75.18	C25 H28 N6 O S	[M+H]+	461.2105	461.2118	-1.3	-2.82	78.77	15.0

Figure S52. Compound **7b** HRMS report

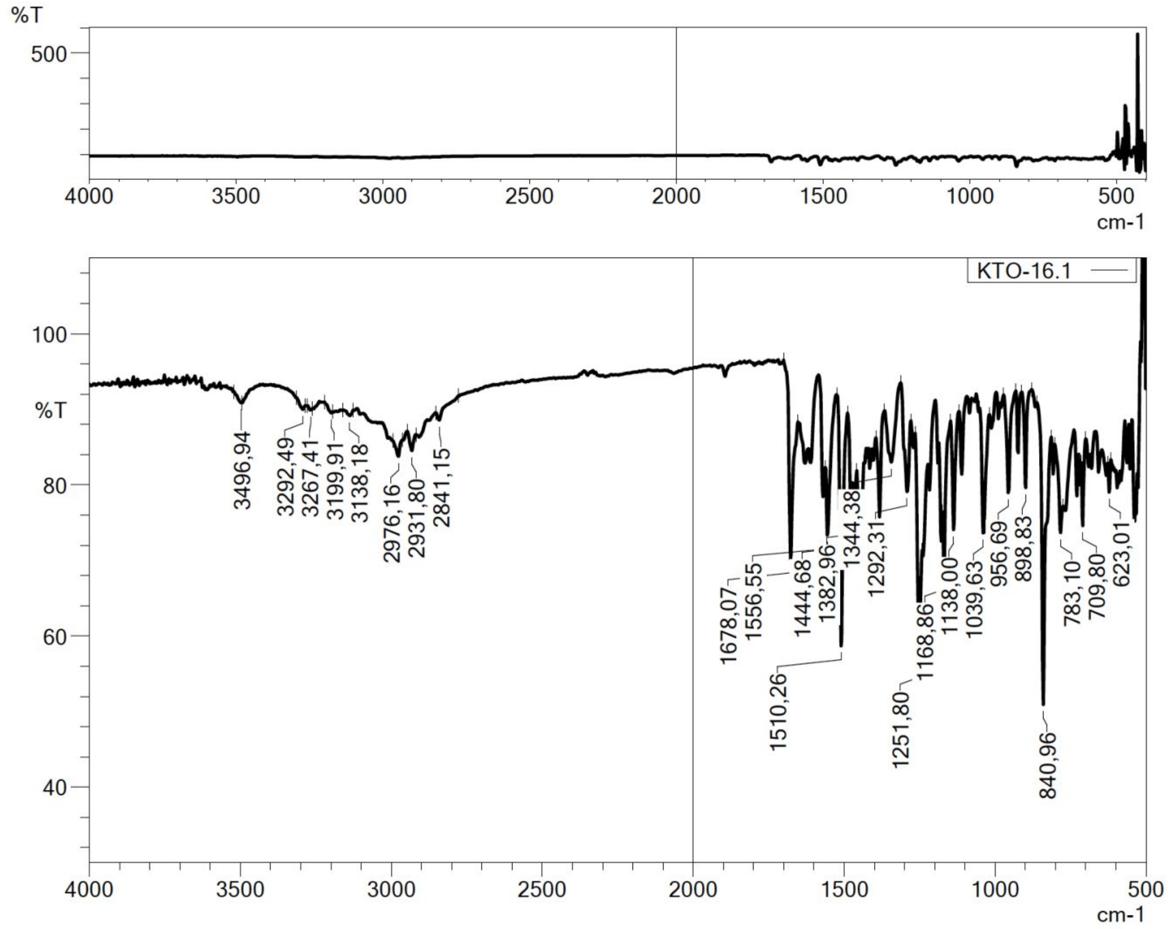

Figure S53. Compound **7c** IR report

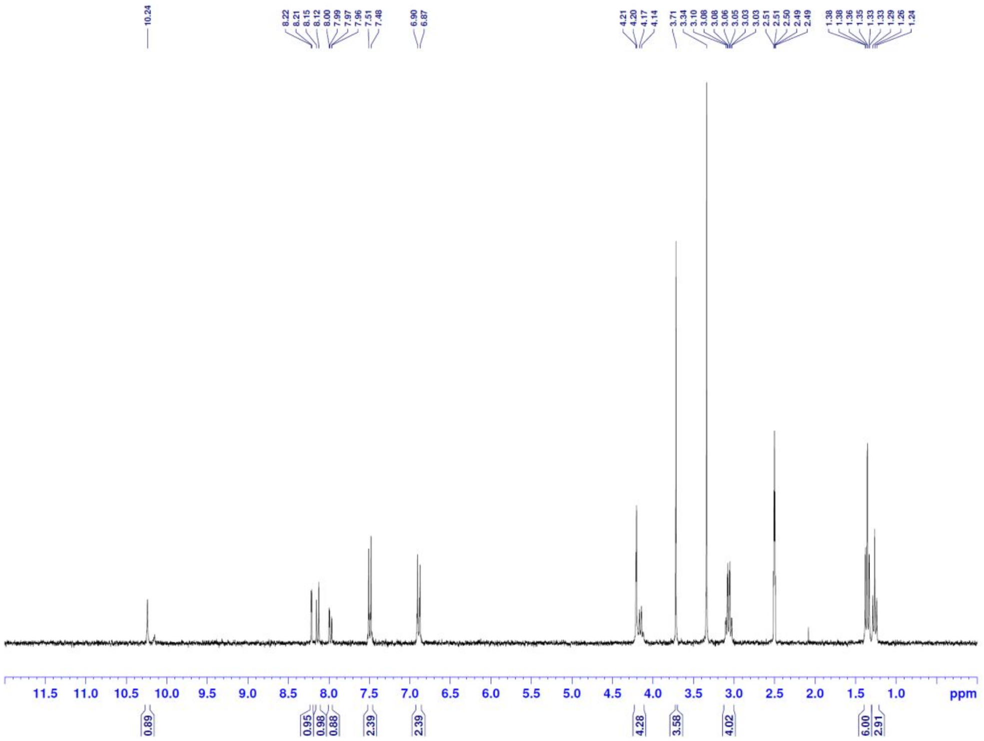

Figure S54. Compound **7c** ^{1}H-NMR spectrum

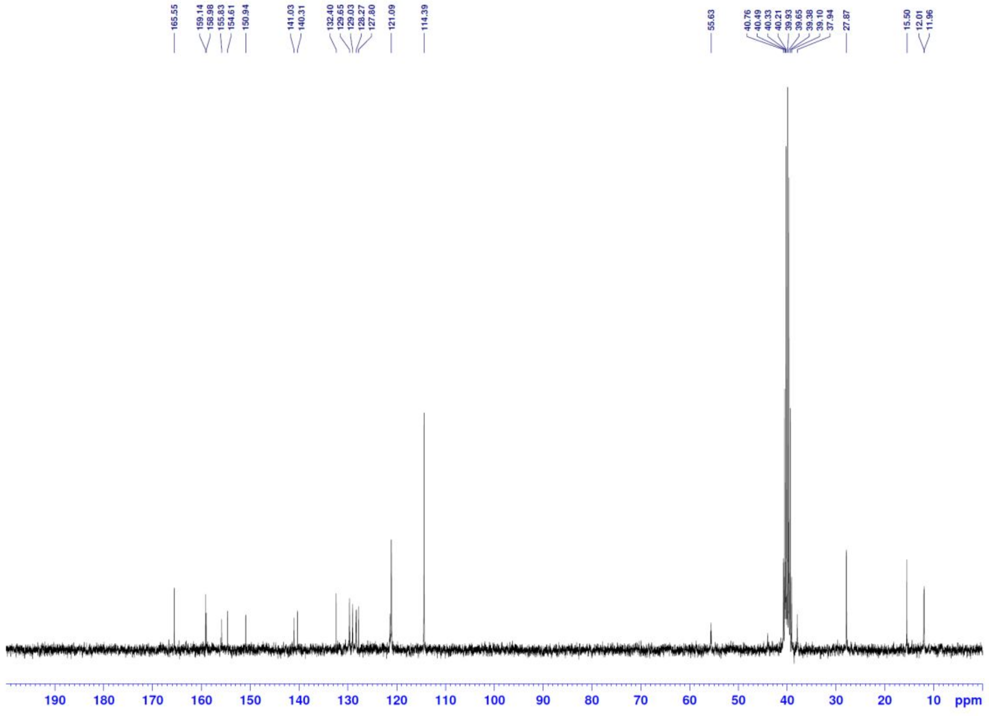

Figure S55. Compound **7c** ^{13}C-NMR spectrum

Formula Predictor Report - KTO-16_199.lcd

Page 1 of 1

Data File: C:\LabSolutions\Data\Analiz\derya\KTO-16_199.lcd

Elmt	Val.	Min	Max	Elmt	Val.	Min	Max	Elmt	Val.	Min	Max	Elmt	Val.	Min	Max	Use Adduct
H	1	8	40	O	2	0	4	S	2	1	1	Ru	2	0	0	H
C	4	9	40	F	1	0	0	Cl	1	0	0	Pd	2	0	0	
N	3	2	6	P	3	0	0	Br	1	0	0	I	3	0	0	

Error Margin (ppm):	5	DBE Range:	5.0 - 25.0	Electron Ions:	both
HC Ratio:	unlimited	Apply N Rule:	yes	Use MSn Info:	yes
Max Isotopes:	3	Isotope RI (%):	1.00	Isotope Res:	9000
MSn Iso RI (%):	10.00	MSn Logic Mode:	AND	Max Results:	200

Event#: 1 MS(E+) Ret. Time : 3.053 Scan# : 459

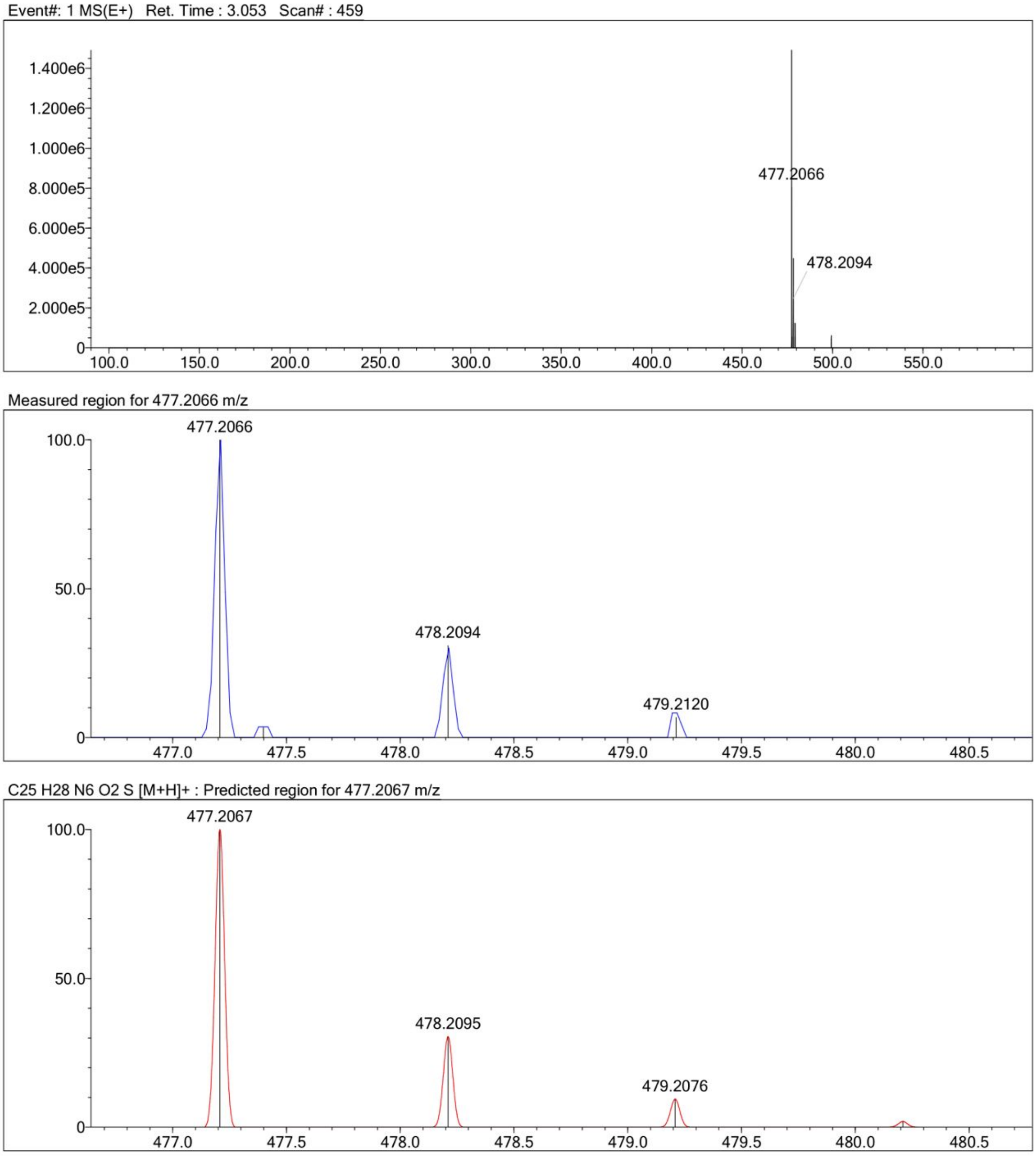

Rank	Score	Formula (M)	Ion	Meas. m/z	Pred. m/z	Df. (mDa)	Df. (ppm)	Iso	DBE
1	81.46	C25 H28 N6 O2 S	[M+H]+	477.2066	477.2067	-0.1	-0.21	81.46	15.0

Figure S56. Compound **7c** HRMS report

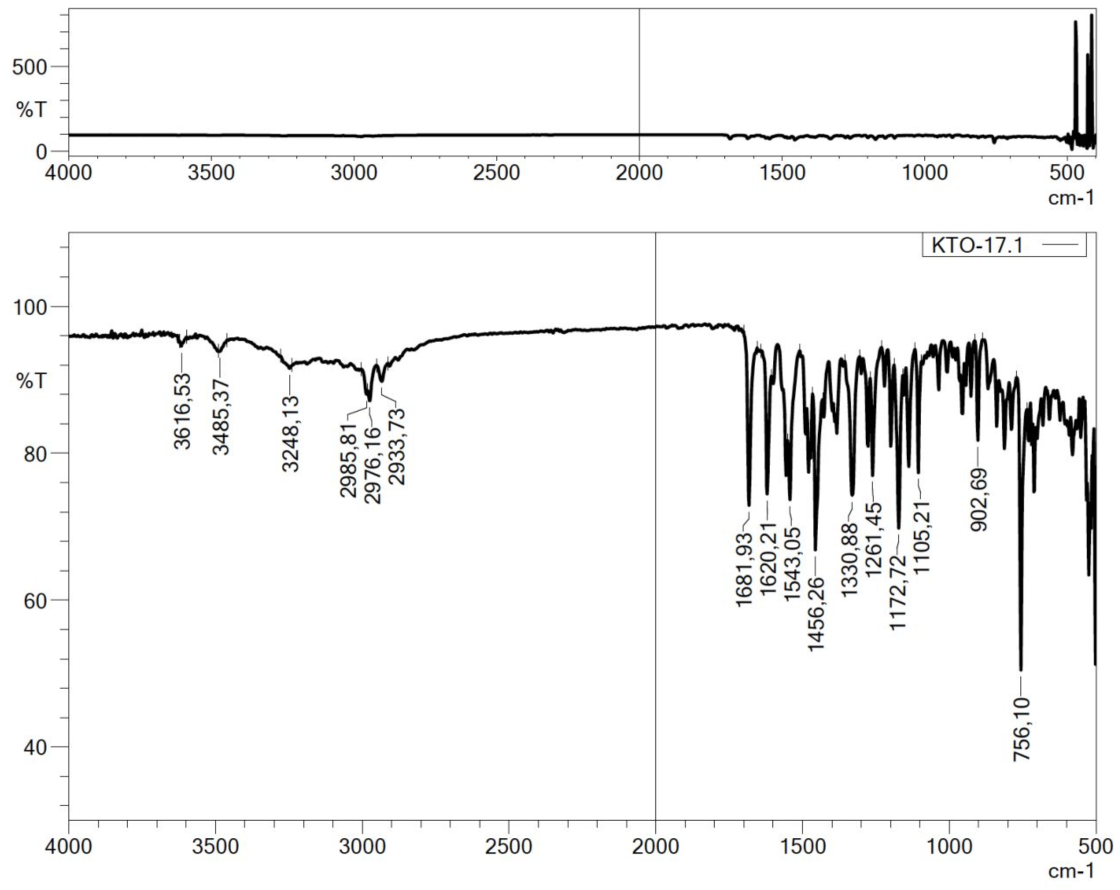

Figure S57. Compound **7d** IR report

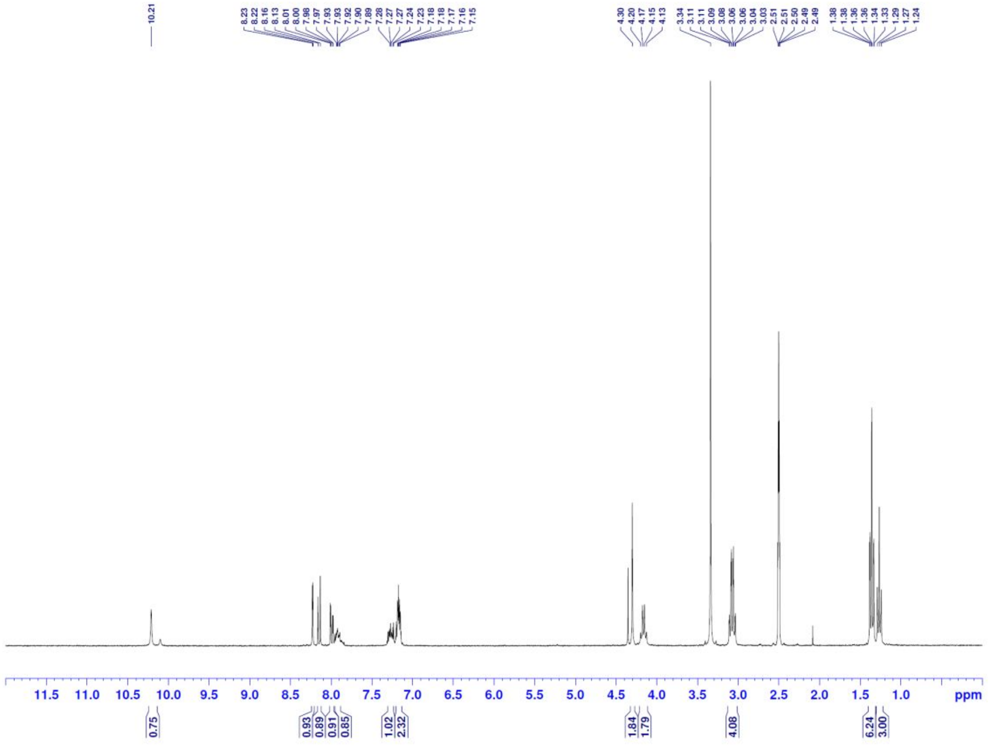

Figure S58. Compound **7d** ^{1}H-NMR spectrum

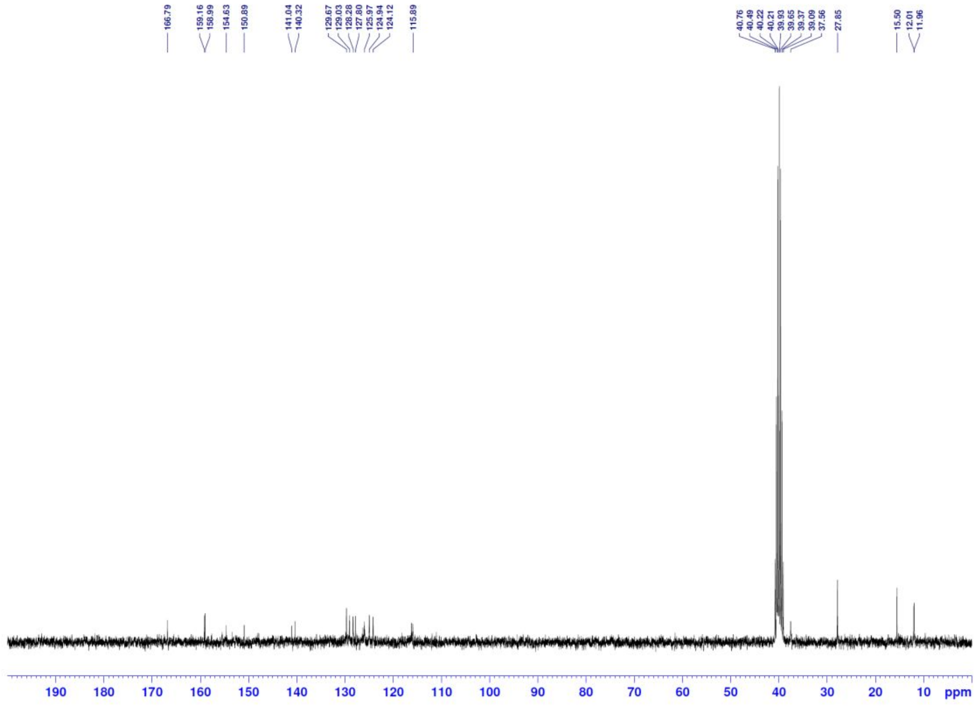

Figure S59. Compound **7d** ^{13}C-NMR spectrum

Data File: C:\LabSolutions\Data\Analiz\derya\KTO-17_200.lcd

Elmt	Val.	Min	Max	Elmt	Val.	Min	Max	Elmt	Val.	Min	Max	Elmt	Val.	Min	Max	Use Adduct
H	1	8	40	O	2	0	4	S	2	1	1	Ru	2	0	0	H
C	4	9	40	F	1	1	1	Cl	1	0	0	Pd	2	0	0	
N	3	2	6	P	3	0	0	Br	1	0	0	I	3	0	0	

Error Margin (ppm):	5	DBE Range:	5.0 - 25.0	Electron Ions:	both
HC Ratio:	unlimited	Apply N Rule:	yes	Use MSn Info:	yes
Max Isotopes:	3	Isotope RI (%):	1.00	Isotope Res:	9000
MSn Iso RI (%):	10.00	MSn Logic Mode:	AND	Max Results:	200

Event#: 1 MS(E+) Ret. Time : 3.427 Scan# : 515

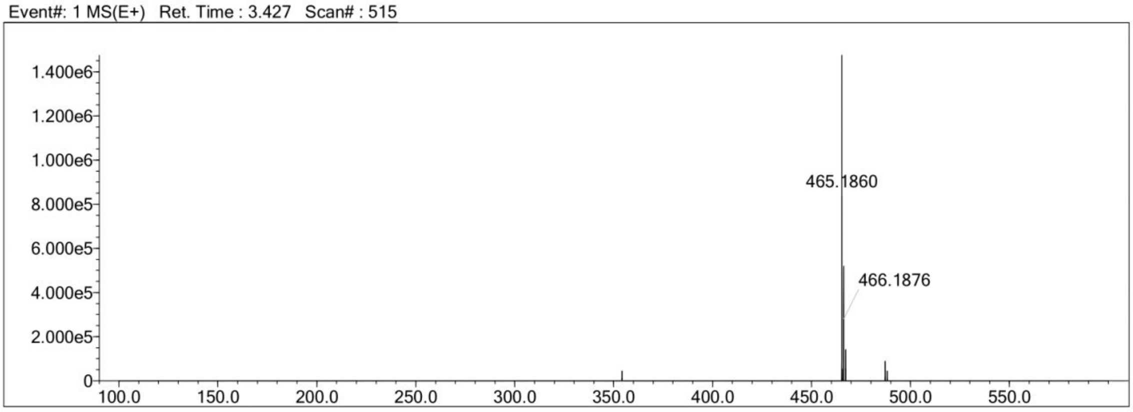

Measured region for 465.1860 m/z

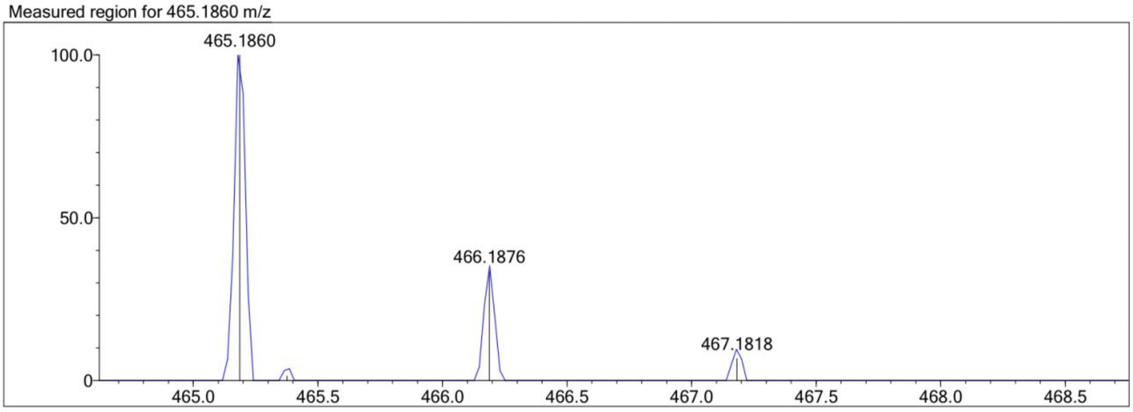

C24 H25 N6 O F S [M+H]+ : Predicted region for 465.1867 m/z

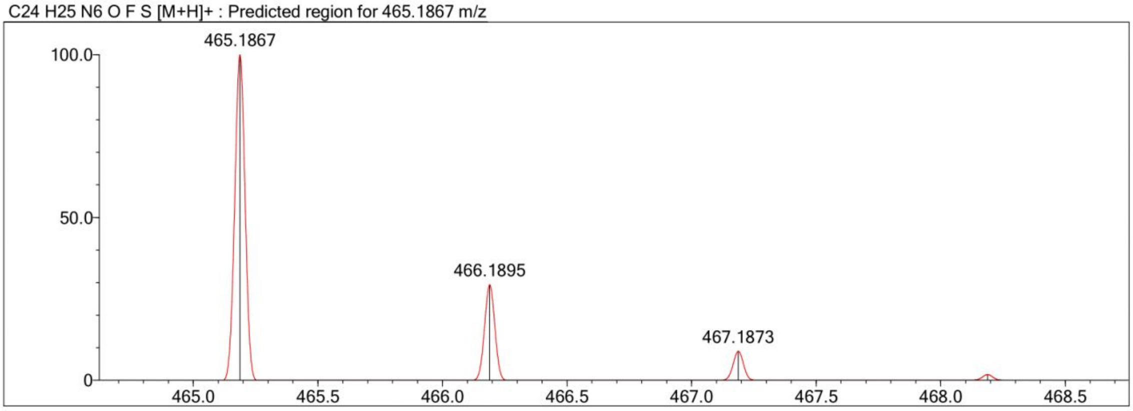

Rank	Score	Formula (M)	Ion	Meas. m/z	Pred. m/z	Df. (mDa)	Df. (ppm)	Iso	DBE
1	69.51	C24 H25 N6 O F S	[M+H]+	465.1860	465.1867	-0.7	-1.50	70.39	15.0

Figure S60. Compound **7d** HRMS report

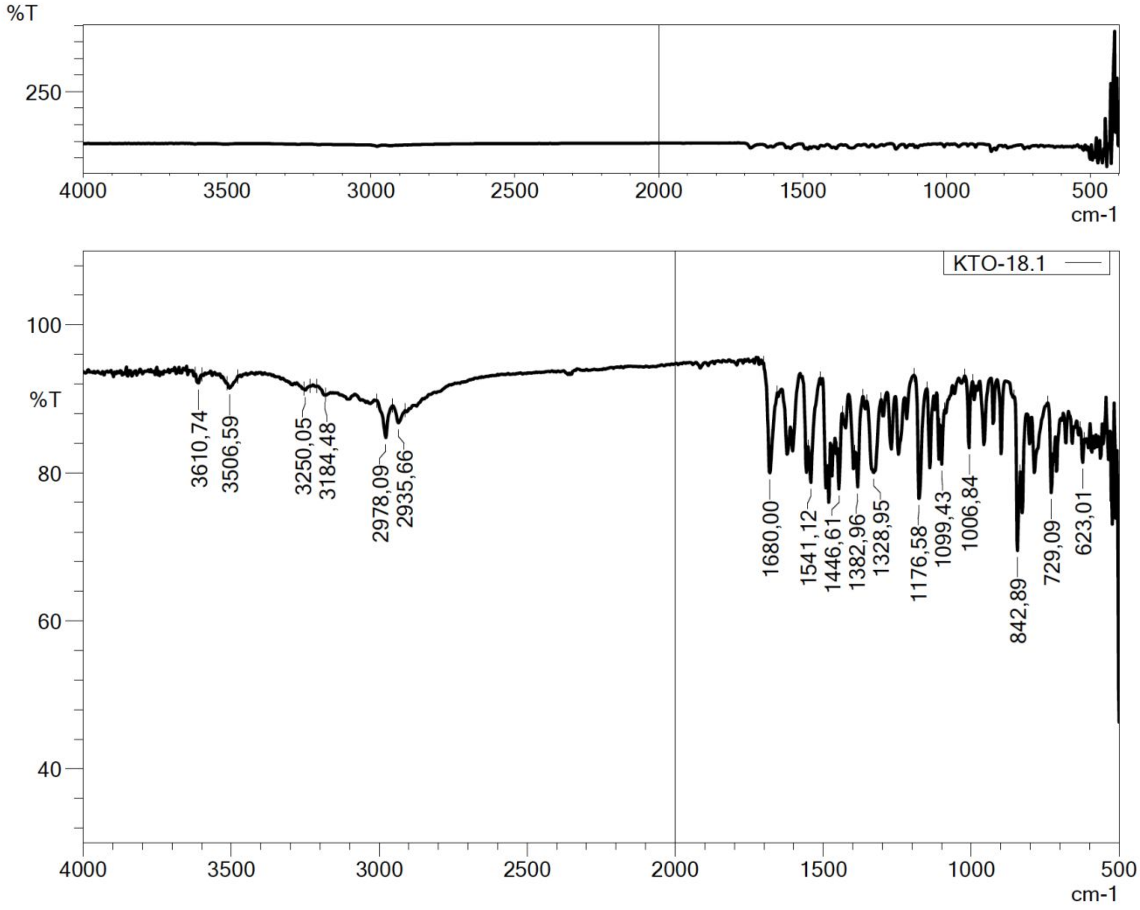

Figure S61. Compound **7e** IR report

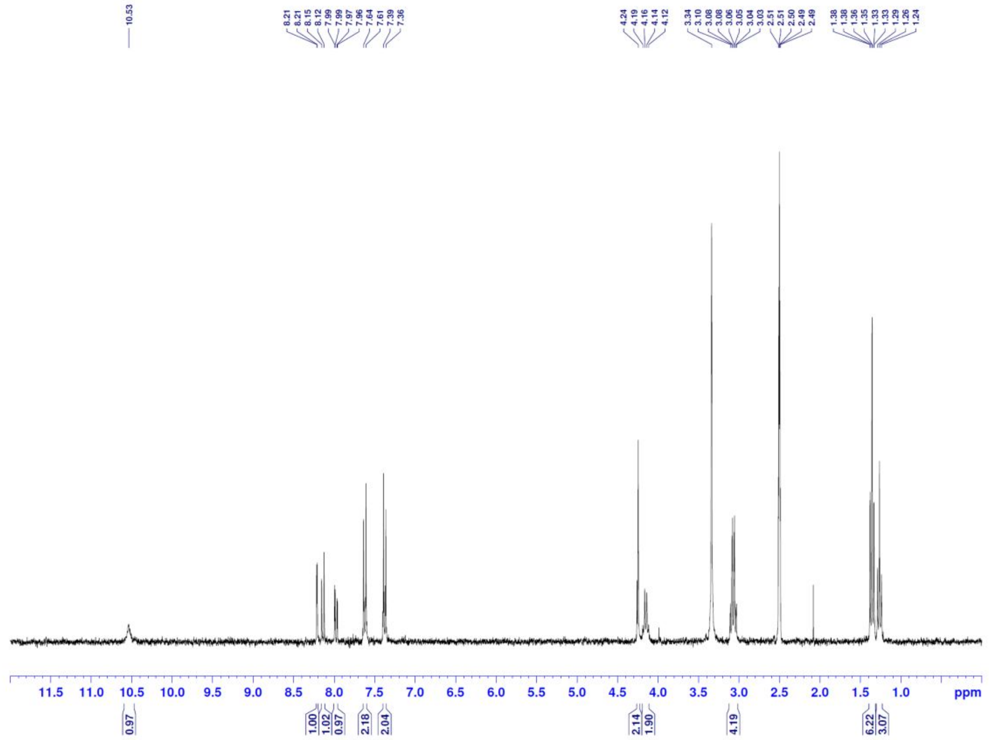

Figure S62. Compound **7e** ^{1}H-NMR spectrum

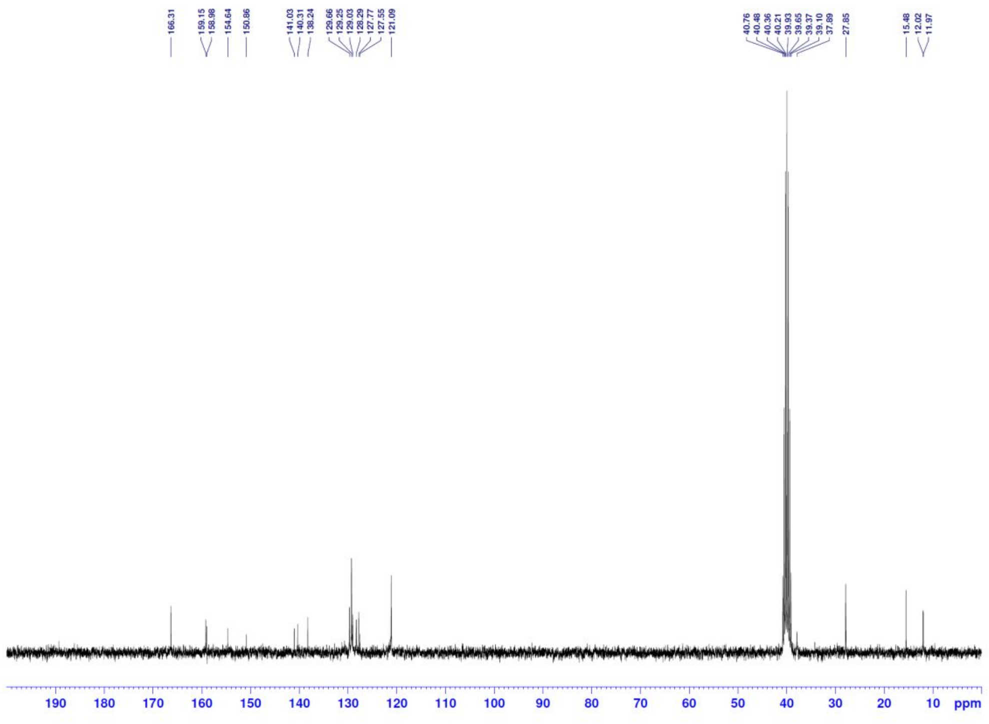

Figure S63. Compound **7e** ^{13}C-NMR spectrum

Formula Predictor Report - KTO-18_201.lcd

Data File: C:\LabSolutions\Data\Analiz\derya\KTO-18_201.lcd

Elmt	Val.	Min	Max	Elmt	Val.	Min	Max	Elmt	Val.	Min	Max	Elmt	Val.	Min	Max	Use Adduct
H	1	8	40	O	2	0	4	S	2	1	1	Ru	2	0	0	H
C	4	9	40	F	1	0	0	Cl	1	1	1	Pd	2	0	0	
N	3	2	6	P	3	0	0	Br	1	0	0	I	3	0	0	

Error Margin (ppm):	5	DBE Range:	5.0 - 25.0	Electron Ions:	both
HC Ratio:	unlimited	Apply N Rule:	yes	Use MSn Info:	yes
Max Isotopes:	3	Isotope RI (%):	1.00	Isotope Res:	9000
MSn Iso RI (%):	10.00	MSn Logic Mode:	AND	Max Results:	200

Event#: 1 MS(E+) Ret. Time : 4.200 Scan# : 631

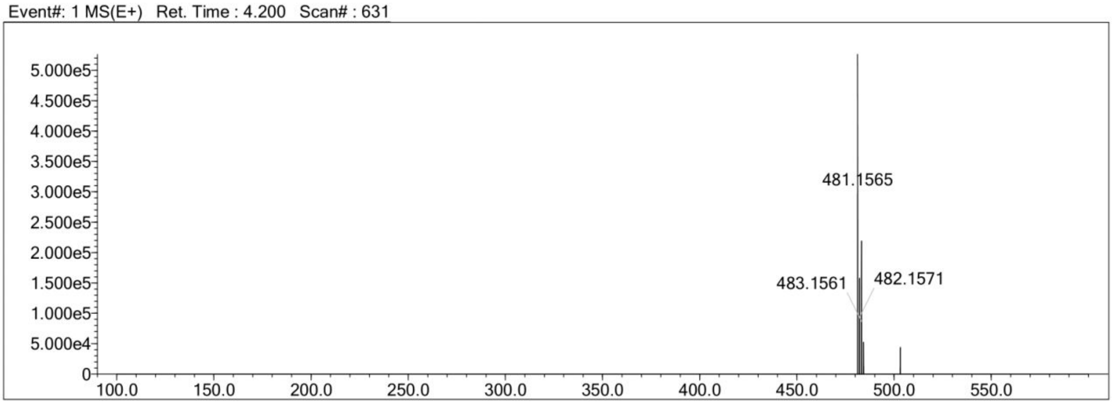

Measured region for 481.1565 m/z

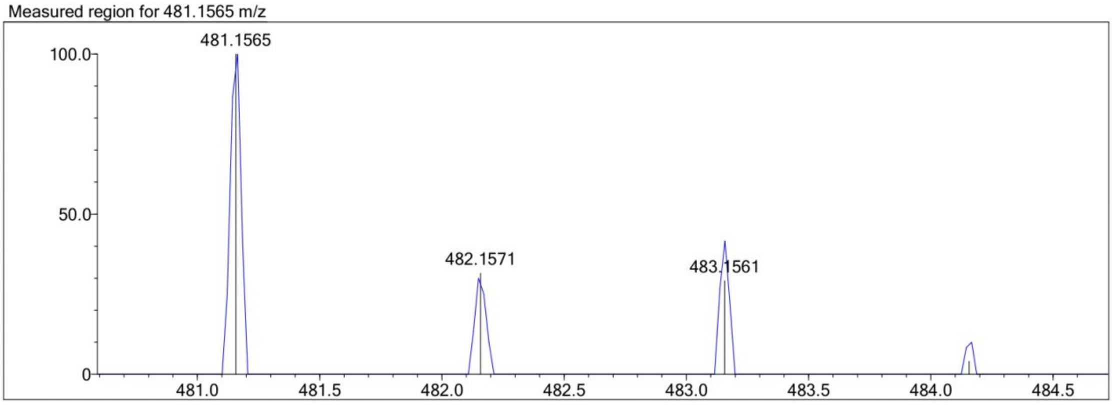

C24 H25 N6 O S Cl [M+H]+ : Predicted region for 481.1572 m/z

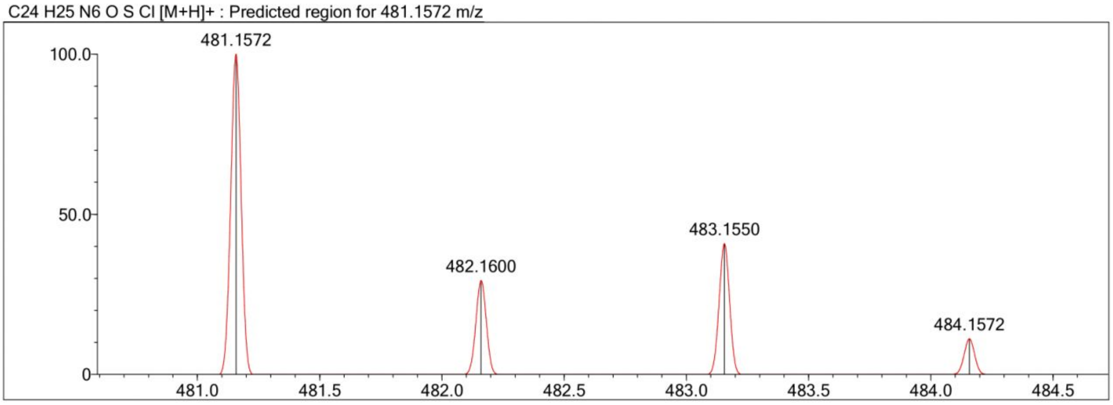

Rank	Score	Formula (M)	Ion	Meas. m/z	Pred. m/z	Df. (mDa)	Df. (ppm)	Iso	DBE
1	91.82	C24 H25 N6 O S Cl	[M+H]+	481.1565	481.1572	-0.7	-1.45	92.86	15.0

Figure S64. Compound **7e** HRMS report

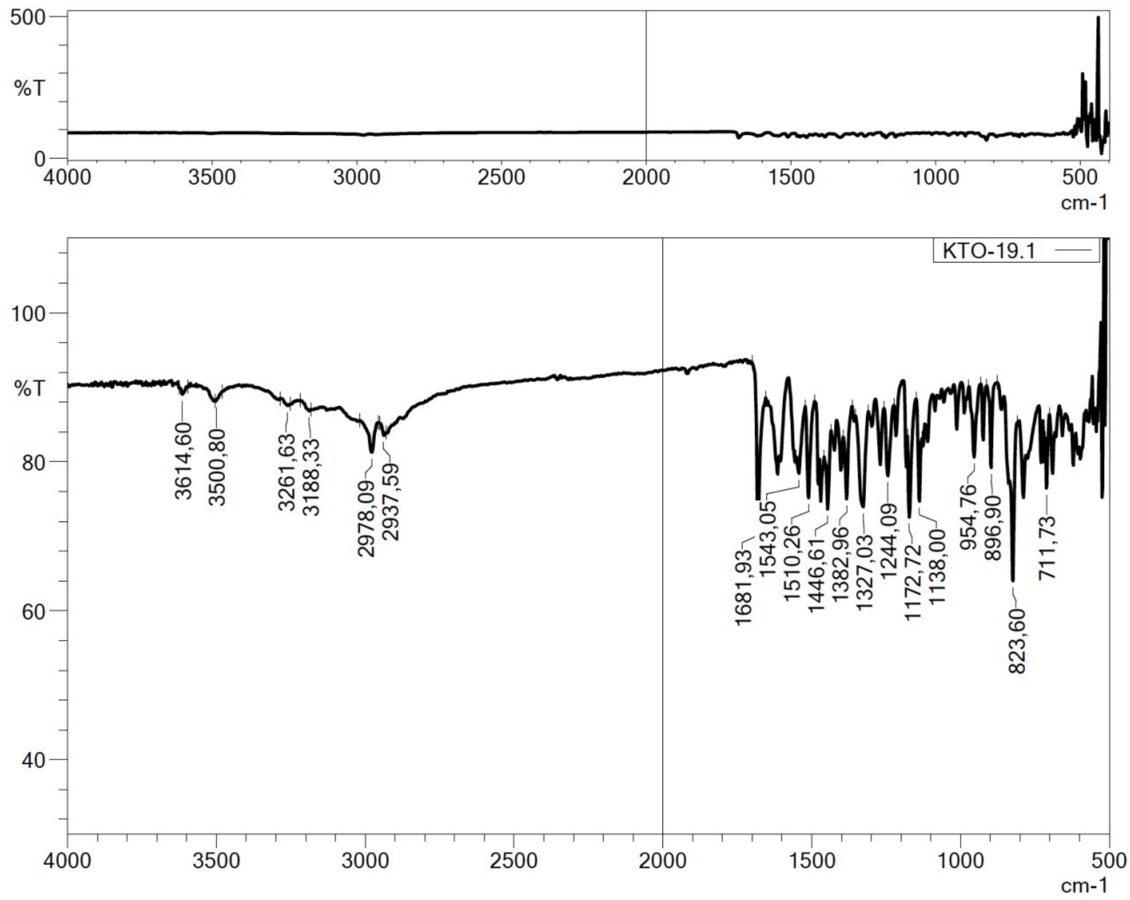

Figure S65. Compound **7f** IR report

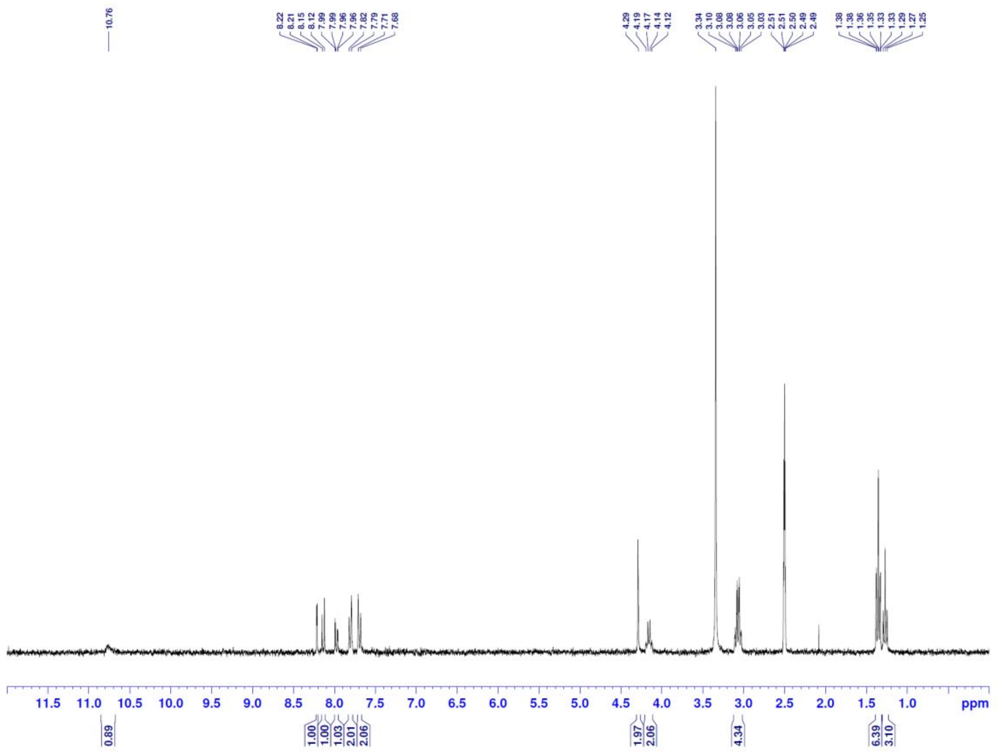

Figure S66. Compound **7f** ^{1}H-NMR spectrum

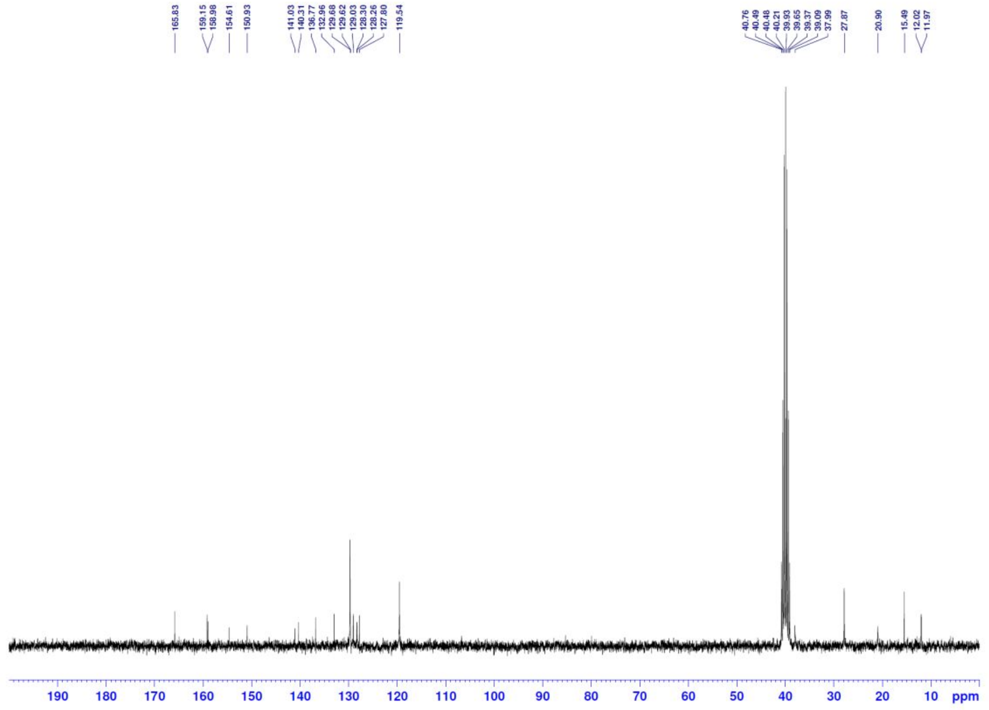

Figure S67. Compound **7f** ^{13}C-NMR spectrum

Data File: C:\LabSolutions\Data\Analiz\derya\KTO-15_198.lcd

Elmt	Val.	Min	Max	Elmt	Val.	Min	Max	Elmt	Val.	Min	Max	Elmt	Val.	Min	Max	Use Adduct
H	1	6	46	O	2	0	6	S	2	1	1	Ru	2	0	0	H
C	4	5	36	F	1	3	3	Cl	1	0	1	Pd	2	0	0	
N	3	0	6	P	3	0	0	Br	1	0	0	I	3	0	0	

Error Margin (ppm):	5	DBE Range:	11.0 - 30.0	Electron Ions:	both
HC Ratio:	unlimited	Apply N Rule:	yes	Use MSn Info:	yes
Max Isotopes:	3	Isotope RI (%):	1.00	Isotope Res:	9000
MSn Iso RI (%):	10.00	MSn Logic Mode:	AND	Max Results:	50

Event#: 1 MS(E+) Ret. Time : 4.733 Scan# : 711

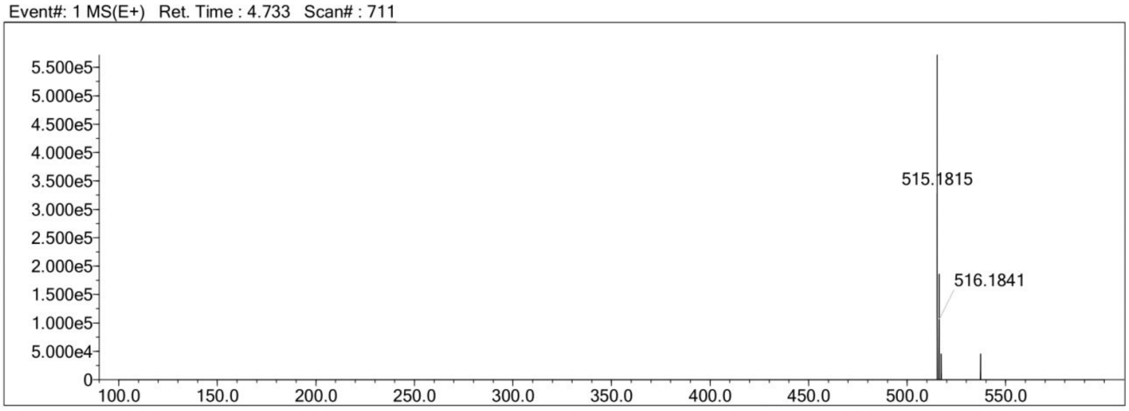

Measured region for 515.1815 m/z

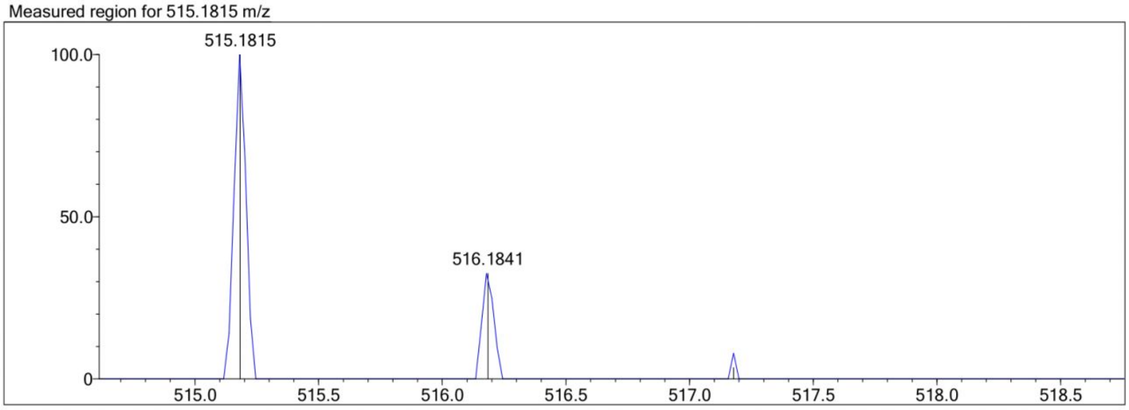

C25 H25 N6 O F3 S [M+H]+ : Predicted region for 515.1835 m/z

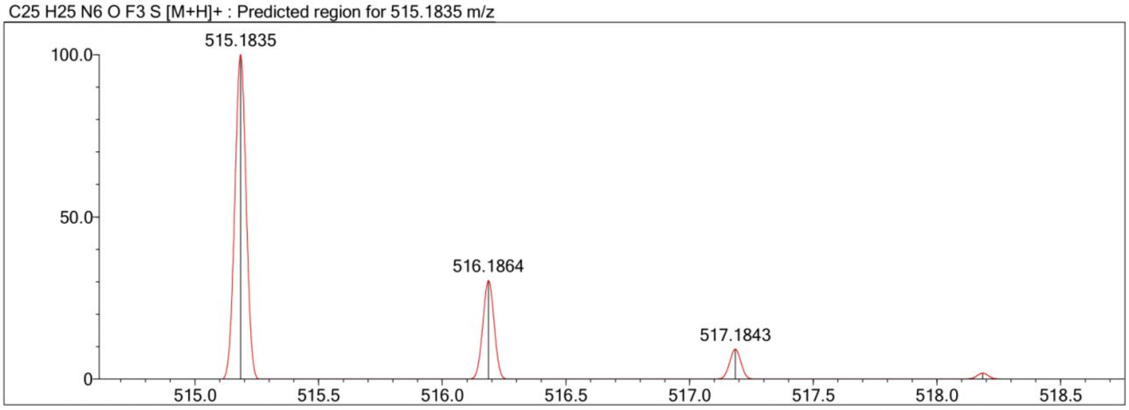

Rank	Score	Formula (M)	Ion	Meas. m/z	Pred. m/z	Df. (mDa)	Df. (ppm)	Iso	DBE
1	64.20	C25 H25 N6 O F3 S	[M+H]+	515.1815	515.1835	-2.0	-3.88	69.18	15.0

Figure S68. Compound **7f** HRMS report
